# Supplementary material for: Antiasthmatic prescriptions in children with and without congenital anomalies: a population-based study
Source: BMJ Open. 2023 Oct 13;13(10):e068885. doi: 10.1136/bmjopen-2022-068885 (PMC10583066; doi:10.1136/bmjopen-2022-068885)
Supplement: Supplementary data [file bmjopen-2022-068885supp001.pdf]

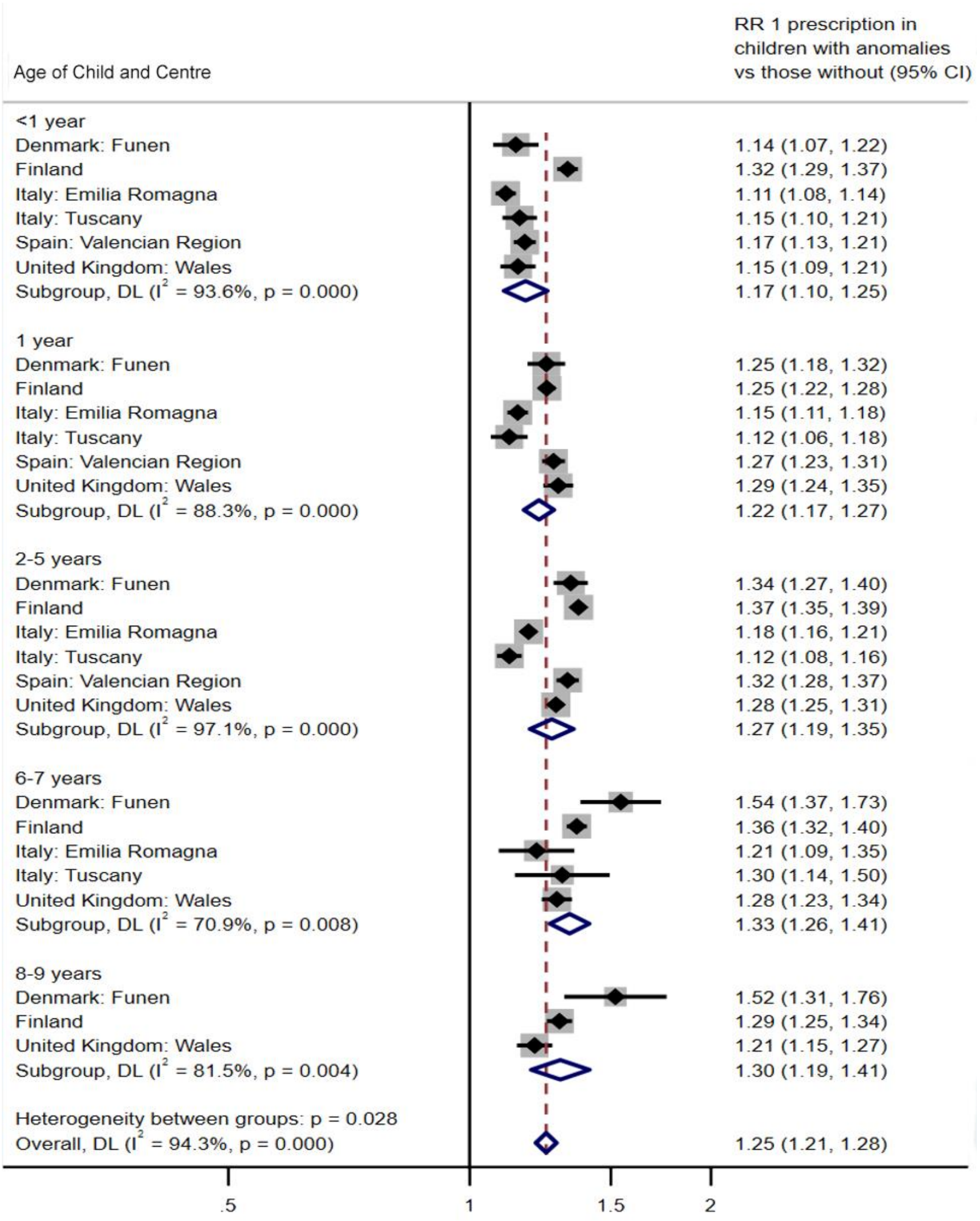

Supplementary Figure 1. Relative risk (RR) of at least 1 prescription for any anti-asthmatic in children with congenital anomalies (CAs) compared to reference children, by registry

CI=Confidence interval,

DL=DerSimonian-Laird

Divin N, *et al. BMJ Open* 2024; 13:e068885. doi: 10.1136/bmjopen-2022-068885
