# Supplementary material for: Antiasthmatic prescriptions in children with and without congenital anomalies: a population-based study
Source: BMJ Open. 2023 Oct 13;13(10):e068885. doi: 10.1136/bmjopen-2022-068885 (PMC10583066; doi:10.1136/bmjopen-2022-068885)
Supplement: Supplementary data [file bmjopen-2022-068885supp002.pdf]

Registry	Birth years included	Average follow-up years (years)	Total number of children in population		Number of children included in the study		Number of children not linked*	
			Children with congenital anomalies	Reference children	Children with congenital anomalies (%)	Reference children (%)	Children with congenital anomalies (%)	Reference children (%)
**Denmark: Funen**	2000-2014	7.3	1,813	72,382	1,789 (98.7)	72,290 (99.9)	24 (1.3)	92 (0.1)
**Finland**	2000-2014	7.2	33,177	762,989	32,926 (99.2)	755,923 (99.1)	251 (0.8)	7,066 (0.9)
**Italy: Emilia Romagna**	2008-2014	4.6	5,801	263,574	5,499 (94.8)	250,829 (95.2)	302 (5.2)	12,745 (4.8)
**Italy: Tuscany**	2008-2014	4.4	3,445	16,844	3,048 (88.5)	16,844 (100)	397 (11.5)	0
**Spain: Valencian Region**	2010-2014	3.6	4,308	223,760	4,281 (99.4)	223,760 (100)	27 (0.6)	0
**UK: Wales**	2000-2014	6.7	15,367	471,882	13,119 (85.4)	403,266 (85.5)	2,248 (14.6)	68,616 (14.5)
**Total**			63,911	1,811,431	60,662 (94.9)	1,722,912 (95.1)	3,249 (5.1)	88,519 (4.9)

*These children were excluded from the study

Supplementary Table 1. Number and percentage of children included in the study by registry
