# Supplementary material for: Antiasthmatic prescriptions in children with and without congenital anomalies: a population-based study
Source: BMJ Open. 2023 Oct 13;13(10):e068885. doi: 10.1136/bmjopen-2022-068885 (PMC10583066; doi:10.1136/bmjopen-2022-068885)
Supplement: Supplementary data [file bmjopen-2022-068885supp004.pdf]

Isolated Anomaly	Any Anti-Asthmatic (RR, 95% CI)	Beta-2 Agonists (RR, 95% CI)	Inhaled Corticosteroids (RR, 95% CI)
**Spina bifida** n = 154	1.68 (1.35-2.09)	2.53 (1.89-3.38)	2.52 (1.97-3.23)
**Hydrocephalus** n = 299	1.59 (1.41-1.80)	2.24 (1.86-2.69)	2.04 (1.71-2.45)
**Severe microcephaly** n = 278	1.53 (1.30-1.79)	2.15 (1.63-2.84)	1.82 (1.44-2.31)
**Anomalies of corpus callosum** n = 471	1.94 (1.70-2.22)	3.44 (2.90-4.08)	2.90 (2.53-3.33)
**Congenital heart defects (CHD)** n = 19,643	1.31 (1.27-1.36)	1.47 (1.40-1.54)	1.48 (1.39-1.57)
**Severe CHD** n = 3,368	1.42 (1.33-1.52)	1.60 (1.47-1.74)	1.83 (1.65-2.02)
**Transposition of great arteries** n = 488	1.46 (1.28-1.66)	1.91 (1.62-2.24)	2.10 (1.81-2.43)
**Ventricular septal defect** n = 11,826	1.21 (1.18-1.24)	1.35 (1.30-1.41)	1.26 (1.20-1.31)
**Atrial septal defect** n = 2,679	1.43 (1.33-1.54)	1.51 (1.37-1.67)	1.53 (1.37-1.70)
**Tetralogy of Fallot** n = 426	1.10 (0.97-1.25)	1.19 (0.94-1.49)	1.52 (1.26-1.84)
**Coarctation of aorta** n = 1,021	1.72 (1.56-1.90)	2.09 (1.85-2.36)	2.12 (1.88-2.38)
**Patent ductus arteriosus** n = 385	1.57 (1.40-1.76)	2.24 (1.81-2.77)	2.23 (1.84-2.71)
**Cleft lip (with or without cleft palate)** n = 1,299	1.27 (1.19-1.36)	1.62 (1.47-1.80)	1.31 (1.18-1.45)
**Cleft palate** n = 1,164	1.49 (1.35-1.65)	1.79 (1.49-2.15)	1.71 (1.49-1.96)
**Oesophageal atresia** n = 239	3.57 (3.14-4.06)	5.75 (4.86-6.81)	5.46 (4.46-6.68)
**Anorectal atresia** n = 236	1.40 (1.20-1.64)	1.92 (1.48-2.49)	1.82 (1.45-2.28)
**Diaphragmatic hernia** n = 211	2.28 (1.72-3.03)	3.70 (2.85-4.81)	3.34 (2.48-4.50)
**Gastroschisis** n = 376	1.45 (1.27-1.67)	1.85 (1.52-2.25)	1.44 (1.15-1.80)

**Multicystic renal dysplasia** n = 621	1.31 (1.18-1.46)	1.83 (1.58-2.11)	1.56 (1.33-1.83)
**Congenital hydronephrosis** n = 3,026	1.21 (1.15-1.28)	1.33 (1.20-1.46)	1.27 (1.18-1.38)
**Hypospadias** n = 2,526	1.30 (1.22-1.39)	1.52 (1.37-1.69)	1.44 (1.32-1.57)
**Limb reduction defects** n = 445	1.16 (1.03-1.32)	1.40 (1.14-1.72)	1.32 (1.06-1.64)
**Club foot** n = 1,997	1.31 (1.24-1.38)	1.46 (1.34-1.59)	1.42 (1.31-1.54)
**Hip dislocation and/or dysplasia** n = 1,026	1.05 (0.96-1.15)	1.03 (0.91-1.17)	1.22 (1.07-1.39)
**Craniosynostosis** n = 765	1.37 (1.26-1.49)	1.70 (1.50-1.93)	1.46 (1.27-1.67)
**All chromosomal** n = 3,608	2.19 (2.00-2.40)	2.86 (2.47-3.31)	2.94 (2.58-3.34)
**Down syndrome** n = 1,993	2.19 (2.00-2.40)	2.77 (2.40-3.20)	2.88 (2.54-3.28)
**Down syndrome (including CHD)** n = 1,131	2.33 (2.09-2.61)	2.93 (2.47-3.48)	3.12 (2.70-3.61)
**Down syndrome (excluding CHD)** n = 862	2.11 (1.93-2.31)	2.76 (2.39-3.19)	2.73 (2.31-3.23)
**Turner syndrome** n = 143	1.48 (1.21-1.82)	2.50 (1.91-3.28)	2.15 (1.64-2.82)
**Di George syndrome** n = 172	3.47 (3.04-3.95)	5.11 (4.35-6.01)	4.88 (4.01-5.94)
**Noonan syndrome** n = 120	2.33 (1.99-2.72)	2.76 (2.09-3.64)	3.68 (2.82-4.82)

Supplementary Table 3. Combined relative risks (RR) across registries for >1 anti-asthmatic prescriptions up to age eight years, by anomaly
