# Supplementary material for: Antiasthmatic prescriptions in children with and without congenital anomalies: a population-based study
Source: BMJ Open. 2023 Oct 13;13(10):e068885. doi: 10.1136/bmjopen-2022-068885 (PMC10583066; doi:10.1136/bmjopen-2022-068885)
Supplement: Supplementary data [file bmjopen-2022-068885supp005.pdf]

	RR of receiving ≥1 prescription for any anti-asthmatic compared to reference children	RR of receiving >1 prescription for any anti-asthmatic compared to reference children
**<1 year**	1.17 (1.10-1.25)	1.39 (1.17-1.65)
**1 year**	1.22 (1.17-1.27)	1.41 (1.29-1.55)
**2-5 years**	1.27 (1.19-1.35)	1.44 (1.35-1.53)
**6-7 years**	1.33 (1.26-1.41)	1.43 (1.32-1.54)
**8-9 years**	1.30 (1.19-1.41)	1.37 (1.25-1.51)
**All years combined**	1.25 (1.21-1.28)	1.41 (1.35-1.48)

Supplementary Table 4. Relative risk (RR) of receiving ≥1 prescription versus receiving >1 prescription for any anti-asthmatic in children with congenital anomalies compared to reference children
