# Supplementary material for: Antiasthmatic prescriptions in children with and without congenital anomalies: a population-based study
Source: BMJ Open. 2023 Oct 13;13(10):e068885. doi: 10.1136/bmjopen-2022-068885 (PMC10583066; doi:10.1136/bmjopen-2022-068885)
Supplement: Supplementary data [file bmjopen-2022-068885supp003.pdf]

| Type of Anomaly* | Congenital Anomaly | ICD10-BPA Code | ICD9 Code |
|---|---|---|---|
| Isolated* | Spina bifida | Q05 | 741 |
| | Hydrocephalus | Q03 | 7423 |
| | Anomalies of the corpus callosum | Q040 | 74221 |
| | Severe microcephaly | Q02 | 7421 |
| | Congenital heart defects (CHD)** | Q20-Q26 | 745, 746, 7470-7474 |
| | Severe CHD*** | Q200, Q201, Q203, Q204, Q212, Q213, Q220, Q224, Q225, Q226, Q230, Q232, Q233, Q234, Q251, Q252, Q262 | 74500, 74510, 7452, 7453, 7456, 7461, 7462, 74600, 7463, 7465, 7466, 7467, 7471, 74720, 74742 |
| | Transposition of great arteries | Q203 | 74510 |
| | Ventricular septal defect | Q210 | 7454 |
| | Atrial septal defect | Q211 | 7455 |
| | Tetralogy of Fallot | Q213 | 7452 |
| | Coarctation of the aorta | Q251 | 7471 |
| | Patent ductus arteriosus | Q250 | 7470 |
| | Cleft lip (with or without cleft palate) | Q36, Q37 | 7491, 7492 |
| | Cleft palate | Q35 | 7490 |
| | Oesophageal atresia | Q390-Q391 | 75030-75031 |
| | Anorectal atresia | Q420-Q423 | 75121-75124 |
| | Diaphragmatic hernia | Q790 | 75661 |
| | Gastroschisis | Q793 | 75671 |
| | Multicystic renal dysplasia | Q6140, Q6141 | 75316 |
| | Congenital hydronephrosis | Q620 | 75320 |
| | Hypospadias | Q54 | 75260 |
| | Limb reduction defects | Q71-Q73 | 7552-7554 |
| | Club foot | Q660 | 75450 |
| | Hip dislocation and/or dysplasia | Q650-Q652, Q6580, Q6581 | 75430 |
| | Craniosynostosis | Q750 | 75600 |
| Chromosomal | Down syndrome | Q90 | 7580 |
| | Down syndrome without CHD | Q90 excluding codes Q20-Q26 | 7580 excluding codes 745, 746, 7470-7474 |

| | | | |
|---|---|---|---|
| | Down syndrome with CHD | Q90 including codes Q20-Q26 | 7580 including codes 745, 746, 7470-7474 |
| | Turner syndrome | Q96 | 75860, 75861, 75862, 75869 |
| Genetic syndromes | Di George syndrome | D821 | 27910 |
| | Noonan syndrome | Q8714 | 759896 |

**Supplementary Table 2.** Congenital anomalies and relevant International Classification of Diseases (ICD)-British Paediatric Association (BPA) codes

*Based on the EUROCAT algorithm [13]

**CHD includes all the anomalies listed under severe CHD, see below, as well as atrial septal defect, ventricular septal defect, pulmonary valve stenosis and patent ductus arteriosus in term infants

***Severe CHD included the following CHD subgroups: common arterial truncus, double outlet right ventricle, transposition of great vessels, single ventricle, atrioventricular septal defect, tetralogy of Fallot, pulmonary valve atresia, tricuspid atresia and stenosis, Ebstein anomaly, hypoplastic right heart, aortic valve atresia/stenosis, mitral valve anomalies, hypoplastic left heart, coarctation of aorta, aortic atresia/interrupted aortic arch, total anomalous pulmonary venous return.
