# Supplementary material for: Antiasthmatic prescriptions in children with and without congenital anomalies: a population-based study
Source: BMJ Open. 2023 Oct 13;13(10):e068885. doi: 10.1136/bmjopen-2022-068885 (PMC10583066; doi:10.1136/bmjopen-2022-068885)
Supplement: Author's manuscript [file bmjopen-2022-068885.draft_revisions.pdf]

BMJ Open

BMJ Open is committed to open peer review. As part of this commitment we make the peer review history of every article we publish publicly available.

When an article is published we post the peer reviewers' comments and the authors' responses online. We also post the versions of the paper that were used during peer review. These are the versions that the peer review comments apply to.

The versions of the paper that follow are the versions that were submitted during the peer review process. They are not the versions of record or the final published versions. They should not be cited or distributed as the published version of this manuscript.

BMJ Open is an open access journal and the full, final, typeset and author-corrected version of record of the manuscript is available on our site with no access controls, subscription charges or pay-per-view fees (http://bmjopen.bmj.com).

If you have any questions on BMJ Open's open peer review process please email info.bmjopen@bmj.com

BMJ Open

Anti-asthmatic prescriptions in children with and without congenital anomalies: a population-based study

Journal:	*BMJ Open*
Manuscript ID	bmjopen-2022-068885
Article Type:	Original research
Date Submitted by the Author:	10-Oct-2022
Complete List of Authors:	Divin, Natalie; Ulster University - Belfast Campus, Centre for Maternal, Foetal and Infant Research<br>Given, Joanne; Ulster University, Institute of Nursing and Health Research<br>Tan, Joachim; St George's University of London, Population Health Research Institute<br>Astolfi, Gianni; University of Ferrara, Department of Neuroscience and Rehabilitation<br>Ballardini, Elisa; University of Ferrara, Italy, Neonatal Intensive Care Unit, Paediatric Section, University of Ferrara, Italy<br>Barrachina-Bonet, Laia; Foundation for the Promotion of Health and Biomedical Research in the Valencian region, Rare Disease Research Unit<br>Cavero- Carbonell, Clara; Foundation for the Promotion of Health and Biomedical Research in the Valencian region, Rare Diseases Research Unit<br>Coi, Alessio; National Research Council Pisa Research Area, Institute of Clinical Physiology<br>Garne, Ester; University of Southern Denmark<br>Gissler, Mika; THL National Institute for Health and Welfare, Information<br>Heino, Anna; Finnish Institute for Health and Welfare<br>Jordan, Susan; Swansea University, Nursing<br>Pierini, Anna; Institute of Clinical Physiology National Research Council<br>Scanlon, Ieuan; Swansea University<br>Urhøj, Stine Kjær ; University of Copenhagen, Section of Epidemiology, Department of Public Health<br>Morris, Joan; St George's, University of London, Population Health Research Institute<br>Loane , Maria ; Ulster University
Keywords:	RESPIRATORY MEDICINE (see Thoracic Medicine), PAEDIATRICS, EPIDEMIOLOGY

SCHOLARONE™
Manuscripts

I, the Submitting Author has the right to grant and does grant on behalf of all authors of the Work (as defined in the below author licence), an exclusive licence and/or a non-exclusive licence for contributions from authors who are: i) UK Crown employees; ii) where BMJ has agreed a CC-BY licence shall apply, and/or iii) in accordance with the terms applicable for US Federal Government officers or employees acting as part of their official duties; on a worldwide, perpetual, irrevocable, royalty-free basis to BMJ Publishing Group Ltd ("BMJ") its licensees and where the relevant Journal is co-owned by BMJ to the co-owners of the Journal, to publish the Work in this journal and any other BMJ products and to exploit all rights, as set out in our licence.

The Submitting Author accepts and understands that any supply made under these terms is made by BMJ to the Submitting Author unless you are acting as an employee on behalf of your employer or a postgraduate student of an affiliated institution which is paying any applicable article publishing charge ("APC") for Open Access articles. Where the Submitting Author wishes to make the Work available on an Open Access basis (and intends to pay the relevant APC), the terms of reuse of such Open Access shall be governed by a Creative Commons licence – details of these licences and which Creative Commons licence will apply to this Work are set out in our licence referred to above.

Other than as permitted in any relevant BMJ Author's Self Archiving Policies, I confirm this Work has not been accepted for publication elsewhere, is not being considered for publication elsewhere and does not duplicate material already published. I confirm all authors consent to publication of this Work and authorise the granting of this licence.

For peer review only

Anti-asthmatic prescriptions in children with and without congenital anomalies: a population-based study

Authors: Natalie Divin PhD[1], Joanne Given PhD[1], Joachim Tan PhD[2], Gianni Astolfi BSc[3], Elisa Ballardini MD[3], Laia Barrachina Bonet MSc[4], Clara Cavero PhD[4], Alessio Coi PhD[5], Ester Garne MD[6], Mika Gissler PhD[7], Anna Heino M.Soc.Sc[7], Susan Jordan PhD[8], Anna Pierini BSc[5], Ieuan Scanlon MSc[8], Stine Kjaer Urhoj PhD[9], Joan K Morris PhD[2], Maria Loane PhD[1]

Affiliations: [1]Ulster University; [2]St George's University of London; [3]Department of Neuroscience and Rehabilitation, University of Ferrara; [4]Rare Diseases Research Unit, Foundation for the Promotion of Health and Biomedical Research of Valencia Region; [5]Unit of Epidemiology of Rare Diseases and Congenital Anomalies, Institute of Clinical Physiology; [6]Pediatric Department, Hospital Lillebaelt Kolding; [7]Finnish Institute for Health and Welfare (THL); [8]Swansea University; [9]University of Copenhagen.

Word count: 3,729 (including legend)

Corresponding author:
Maria Loane
Ulster University, Belfast Campus, Room BC-04-167
York Street,
Belfast, Co Antrim
N Ireland BT15 1ES
Telephone: +44 2895 365256
Email: ma.loane@ulster.ac.uk

For peer review only

Abstract

Objectives: To explore the risk of being prescribed/dispensed medications for respiratory symptoms and breathing difficulties in children with and without congenital anomalies.

Design: A EUROlinkCAT population-based data linkage cohort study. Data on children with and without congenital anomalies were linked to prescription databases to identify children who did/did not receive anti-asthmatic prescriptions. Data were analysed by age, European region, class of anti-asthmatic, anomaly, sex, gestational age, and birth cohort.

Setting: Children born 2000-2014 in six regions within five European countries.

Participants: 60,662 children with congenital anomalies and 1,722,912 reference children up to age ten years.

Primary outcome measure: Relative risks (RR) of >1 anti-asthmatic prescription in a year, identified using Anatomical Therapeutic Chemical classification codes beginning with R03.

Results: There were significant differences in the prescribing of anti-asthmatics in the six regions. Children with congenital anomalies had a significantly higher risk of being prescribed anti-asthmatics (RR=1.41, 95% CI 1.35-1.48) compared to reference children. The increased risk was consistent across all regions and all age groups. Children with congenital anomalies were more likely to be prescribed beta-2 agonists (RR=1.71, 95% CI 1.60-1.83) and inhaled corticosteroids (RR=1.74, 95% CI 1.61-1.87). Children with oesophageal atresia, diaphragmatic hernia, genetic syndromes and chromosomal anomalies had over twice the risk of being prescribed anti-asthmatics compared to reference children. Children with congenital anomalies born <32 weeks gestational age were over twice as likely to be prescribed anti-asthmatics than those born at term (RR=2.20, 95% CI 2.10-2.30).

Conclusion: This study documents the additional burden of respiratory symptoms and breathing difficulties for children with congenital anomalies, particularly those born preterm, compared to children without congenital anomalies in the first ten years of life. These findings are beneficial to clinicians and health care providers as they identify children with greater morbidity associated with respiratory symptoms, as indicated by anti-asthmatic prescriptions.

Strengths and limitations of this study

- This is the first population-based multi-centre study exploring anti-asthmatic prescriptions in children with and without congenital anomalies.
- Data on over 60,000 children with congenital anomalies were analysed, overcoming limitations of previous research based on small, single-centre sample sizes.
- A Common Data Model was used to map and standardise prescription/pharmacy dispensing records across a range of coding classification systems, languages, and healthcare systems in Europe.
- Approximately 5% of children across registries could not be linked to prescription databases, potentially leading to bias.

For peer review only - http://bmjopen.bmj.com/site/about/guidelines.xhtml

- While we examined medications prescribed/dispensed, we could not gauge whether these medications were actually taken.

Key words: Anti-asthmatics; prescriptions; congenital anomalies; population-based; data linkage

For peer review only

Introduction

Breathing difficulties in childhood are a leading cause of emergency department visits, hospitalisations and missed days at school [1]. The prevalence of chronic breathing conditions such as asthma varies globally in children. The US National Health Interview Survey in 2013 reported prevalence rates of 8.3% in children aged 0-17 years based on parent reports of doctor-diagnosed asthma [2]. The International Study of Asthma and Allergies in Childhood (ISAAC) 1999-2004, which was also based on parental reports, found that prevalence of asthma in European children aged 6-7 years varied from <5% in Albania to over 20% in the United Kingdom (UK), with prevalence rates increasing yearly [3-4]. Similarly, the more recent MeDALL (Mechanisms of the Development of ALLergy) study involving eight birth cohorts across Europe found that parental reports of doctor-diagnosed asthma at age four years ranged from 1.7% in Germany to 13.5% in Bradford, England [5].

The prevalence of asthma also varies according to age and sex in childhood. The ISAAC study consistently found higher asthma rates for 13-14 year olds compared with 6-7 year olds, and this pattern of higher asthma prevalence rates in older children has been replicated across Europe [5-7]. However, as asthma can be challenging to diagnose in infants, wheezing and recurrent wheezing have been used as measures of breathing difficulties in infants. A meta-analysis of European, Latin American and African research reported prevalence rates of 36.1% (95% CI 35.2-37.0%) and 17.4% (95% CI 16.7-18.1%) for wheezing and recurring wheezing respectively for children under two years of age [8]. These prevalence rates were higher than rates of current wheeze in the ISAAC study for 6-7 year olds (11.5%) and 13-14 year olds (14.1%) [9]. Boys have an increased likelihood of parent-reported asthma and wheeze [10-12], and receive more anti-asthmatic prescriptions than girls across Denmark, Sweden and Germany [13-15].

Most of the literature on childhood asthma and respiratory conditions does not differentiate between children with and without congenital anomalies, nor does it explore the additional burden of having chronic and frequent breathing difficulties in children with congenital anomalies. A cohort study based on health records of 170,960 Canadian children up to age six, 1980-1990, reported that children with congenital anomalies of the circulatory system and anomalies of the respiratory system had an increased risk of a recorded asthma diagnosis (HR=1.16, 95% CI 1.03-1.30; and HR=1.43, 95% CI 1.27-1.61) compared to control children [16]. Children under two with diaphragmatic hernia had high hospital readmission rates for wheeze, particularly if born preterm [17]. Most research exploring asthma and breathing difficulties in those with congenital anomalies is based on case series involving small sample sizes. For example, a cohort of adult Dutch patients with unrepaired atrial septal defect reported that four out of 31 (13%) patients had an asthma diagnosis and were taking anti-asthmatic medication [18]. Similarly, a study of 14 Dutch children aged 7-12 years with oesophageal atresia identified that four of the 14 children (29%) had received a diagnosis of asthma [19].

The aim of this study is to compare the risk of being prescribed or dispensed medications for respiratory conditions in children with and without congenital anomalies in six European regions.

For peer review only

Materials and Methods

This is a EUROlinkCAT population-based data linkage cohort study involving children from 0-9 years of age with and without congenital anomalies in six European regions: Denmark: Funen; Finland; Italy: Emilia Romagna; Italy: Tuscany; Spain: Valencian Region; UK: Wales.

The inclusion criteria was all liveborn children in the registry areas born between 2000 and 2014, or the first birth year included in the study by each registry (Table 1). Five registries included reference children from the whole population covering the registry area, while Tuscany provided a 10% sample of reference children matched on year of birth and sex. Data on children with congenital anomalies were extracted from the European congenital anomaly (EUROCAT) registries and data on reference children were obtained from the birth registers in each region [20-22].

Children were linked to regional or national prescription databases and to hospital outpatient pharmacy databases, if available. Children who could not be linked to these databases were classified as "not linked" and were not included in the study, see Supplementary Table 1. Wales had information on prescriptions issued by the general practitioner (GP) for approximately 70% of Welsh GP practices that were part of the Secure Anonymised Information Linkage (SAIL) databank during the study period, while the other registries had information on prescriptions dispensed by a pharmacy in their region. None of the registries were able to link to hospital inpatient prescribing data or had access to over the counter medication.

Anti-asthmatic medication is defined using the World Health Organisation's Anatomical Therapeutic Chemical (ATC) classification system, i.e. ATC codes beginning with R03 (drugs for obstructive airway diseases): beta-2 agonists (R03AC); inhaled corticosteroids (R03BA); anticholinergics (R03BB); antiallergic agents (R03BC); and leukotriene receptor antagonists (R03DC). Data on beta-2 agonists and inhaled corticosteroids were explored individually. Due to small numbers, data on the remaining anti-asthmatic types were included in the 'any anti-asthmatic (R03)' category. Children were classed as exposed to anti-asthmatics if they had been prescribed or dispensed >1 anti-asthmatic prescription in a year in order to minimise children presenting with one-time occurrences of wheeze or infection. Children were classified as unexposed if they were prescribed or dispensed less than two anti-asthmatic prescriptions in a year. Data on anti-asthmatic medications were included from the year 2000 or the first birth year included in the study by each registry up to the end of 2015, resulting in at least one year of follow-up information for each child.

Information on prescriptions were standardised according to a Common Data Model developed as part of the EUROlinkCAT study protocol [21-22]. The EUROCAT data on children with congenital anomalies were already standardised according to EUROCAT guidelines [23]. A common analysis script was sent to all registries to produce aggregate data and analytic results. The aggregate data and analytic results were uploaded to a secure web portal for download by the research team; all individual case data were kept locally.

We explored anti-asthmatic medications in children with any major congenital anomaly (defined as a congenital malformation, deformation, disruption or dysplasia) [20-21], and in

For peer review only - http://bmjopen.bmj.com/site/about/guidelines.xhtml

Registry	Average follow-up years (years)	Total number of children included in study		Total receiving >1 anti-asthmatic during whole follow-up		Total receiving >1 inhaled corticosteroid during whole follow-up		Total receiving >1 beta-2 agonist during whole follow-up	
		Children with congenital anomalies	Reference children	Children with congenital anomalies (%)	Reference children (%)	Children with congenital anomalies (%)	Reference children (%)	Children with congenital anomalies (%)	Reference children (%)
**Denmark: Funen (2000-2014)**	7.3	1,789	72,290	614 (34.3%)	20,728 (28.7%)	288 (16.1%)	8,114 (11.2%)	284 (15.9%)	7,684 (10.6%)
**Finland (2000-2014)**	7.2	32,926	755,923	5,617 (17.1%)	99,780 (13.2%)	3,036 (9.2%)	48,866 (6.5%)	3,375 (10.3%)	53,395 (7.1%)
**Italy: Emilia Romagna (2008-2014)**	4.6	5,499	250,829	2,741 (49.8%)	117,675 (46.9%)	1,769 (32.2%)	71,088 (28.3%)	1,087 (19.8%)	41,215 (16.4%)
**Italy: Tuscany (2008-2014)**	4.4	3,048	16,844	1,392 (45.7%)	6,542 (38.8%)	962 (31.6%)	3,947 (23.4%)	560 (18.4%)	1,861 (11.0%)
**Spain: Valencian Region (2010-2014)**	3.6	4,281	223,760	1,816 (42.4%)	77,028 (34.4%)	352 (8.2%)	8,692 (3.9%)	740 (17.3%)	25,518 (11.4%)
**UK: Wales (2000-2014)**	6.7	13,119	403,266	2,540 (19.4%)	60,230 (14.9%)	1,246 (9.5%)	26,732 (6.6%)	2,018 (15.4%)	46,934 (11.6%)
**Total**		60,662	1,722,912	14,720 (24.3%)	381,983 (22.2%)	7,653 (12.6%)	167,439 (9.7%)	8,064 (13.3%)	176,607 (10.3%)

Table 1. Number and percentages of children with congenital anomalies and reference children prescribed >1 anti-asthmatic medication, by registry

For peer review only - http://bmjopen.bmj.com/site/about/guidelines.xhtml

32 isolated congenital anomaly subgroups with a livebirth prevalence of ≥1.75 per 10,000 births to avoid potential issues with small numbers [21] (Supplementary Table 2). Isolated anomalies are defined as anomalies within a single organ, as defined using the EUROCAT algorithm [23].

Each participating registry obtained local approvals for their data to be used in the EUROlinkCAT project. Ethical approval for this study was obtained from Ulster University's Nursing and Health Research Ethics Filter Committee (Reference Number: FCNUR-21-060).

Patient and public involvement

No patient involved.

Statistical analysis

Age groups were collapsed into six categories to avoid disclosive counts: <1 year; 1 year; 2-3 years; 4-5 years; 6-7 years; 8-9 years. Data were available from Valencian Region for children 0-5 years of age (i.e. up to the children's 6th birthday), from Italian registries for children 0-7 years of age (i.e. up to 8th birthday), and from Funen, Finland and Wales for children 0-9 years of age (i.e. up to 10th birthday). Analyses of specific congenital anomalies were conducted using data for children aged 0-7 years to include data from the Italian registries.

DerSimonian-Laird (DL) random-effects meta analyses were conducted to identify both the relative risk (RR) of receiving anti-asthmatic prescriptions relative to the reference group, and the heterogeneity of prevalence rates across registries and by age group [24]. Random-effects meta-analyses were used to estimate RR ratios for the 32 congenital anomaly subgroups, all registries combined, and by class of anti-asthmatic i.e. any anti-asthmatic, inhaled corticosteroids and beta-2 agonists. Heterogeneity was estimated by the statistic I^2. Stata Version 17 was used for data analysis [25].

We explored the relative risk of receiving >1 of any anti-asthmatic for children with and without congenital anomalies for three potential risk factors: birth cohort, sex of child, and gestational age (GA). Birth cohort was examined for 2000-2004 (baseline group) and 2005-2009, using data from Funen, Finland and Wales as these were the only regions that had data on children born 2000-2004. For the remaining analyses, risk factors were examined for the 0-5 age group for all regions. Girls were used as the baseline group for sex of child analyses. Birth at 37+ weeks GA was used as the baseline group in comparison to birth at <32 weeks GA and birth at 32-36 weeks GA.

Classifying children as exposed to anti-asthmatics if they had been prescribed or dispensed >1 anti-asthmatic prescription in a year was relaxed in a sensitivity analysis to include children with only 1 prescription/dispensation in a year.

For peer review only - http://bmjopen.bmj.com/site/about/guidelines.xhtml

Results

A total of 63,911 children with congenital anomalies and 1,811,431 reference children up to and including nine years of age were included from six national/regional databases in five countries. Of these, 60,662 (94.9%) children with congenital anomalies and 1,722,912 reference children (95.1%) were included in the study. The percentage of children with and without congenital anomalies prescribed anti-asthmatic medication in each region are shown in Table 1. Overall, 5% of children (ranging from 0% in Tuscany and Valencian Region to 14.6% in Wales) were not included in the study as they could not be linked.

Prescriptions for any anti-asthmatic medicines

There were considerable geographical variations in age-specific prevalence rates of any anti-asthmatic prescription (Figure 1). However, the pattern of prescriptions according to age was similar for children with and without congenital anomalies in all registries. In four registries, the prevalence of >1 prescription for any anti-asthmatic in children with congenital anomalies and reference children peaked at the younger ages (<1 and 1 year), then decreased sharply with age. In Finland and Wales, the prevalence increased until age 2-3 years for all children and then stabilised. Geographical differences in prevalence of >1 prescription for any anti-asthmatic levelled off by age 6-7 years, with rates in children with congenital anomalies ranging between 6-9% in all registries, and rates for reference children ranging between 4-6% in all registries. Prevalence rates for >1 prescription for any anti-asthmatics were consistently slightly higher in children with congenital anomalies than reference children across registries and age groups (Figure 1).

Children with congenital anomalies had a 41% higher risk of >1 prescription for any anti-asthmatic compared to reference children across all age groups and across all registries (RR=1.41, 95% CI 1.35-1.48) (Figure 2). Children aged <1 year with congenital anomalies in Finland had an almost two-fold increase in risk compared to reference children (RR=1.97; 95% CI 1.86-2.08). The wider CIs in older age groups in some registries indicate smaller sample sizes as there were fewer children with long follow-ups. Statistically significant heterogeneity was identified between registries at all ages (I^2 = 94.3%, p < .001) due to the large number of reference children, but the differences were small in magnitude.

Prescriptions for inhaled corticosteroids

Children with congenital anomalies had a higher risk of receiving >1 prescription for inhaled corticosteroids than reference children. However, there was heterogeneity between age groups (p = 0.004). The risk was highest for children with congenital anomalies aged <1 year (RR=2.12, 95% CI 1.50-2.99) and steadily decreased to 1.35 (95% CI 1.22-1.49) in children aged 8-9 years (Figure 3). The relative risks of receiving >1 prescription for inhaled corticosteroids appeared consistently higher in Valencian Region in children <1 year of age (RR=3.24, 95% CI 2.74-3.84), children at 1 year of age (RR=2.67, 95% CI 2.30-3.10), and children at 2-5 years (RR=2.16, 95% CI 1.91-2.45) compared to children in other registries. Again, there was significant statistical heterogeneity across registries for all ages (I^2 = 95.6%, p < .001) due to the large sample sizes.

For peer review only - http://bmjopen.bmj.com/site/about/guidelines.xhtml

Prescriptions for beta-2 agonists

	Children with congenital anomalies (RR; 95% CI)	Reference children (RR; 95% CI)
**Birth Cohort**		

The RR of receiving >1 prescription for beta-2 agonists was 71% higher in children with congenital anomalies compared to reference children (RR=1.71, 95% CI 1.60-1.83) (Figure 3). In contrast to inhaled corticosteroids, the risk of receiving >1 prescription for beta-2-agonists remained consistently high across all age groups (heterogeneity between age groups p = 0.358). Significant heterogeneity was identified across registries for all ages (I^2 = 94.4%, p < .001).

Risk factors for receiving anti-asthmatic prescriptions (children up to age six years)

Children aged 0-5 years were more likely to receive >1 anti-asthmatic per year in 2005-2009 compared to 2000-2004, with this risk being higher in reference children than children with congenital anomalies (Table 2). Children with congenital anomalies born at <32 weeks GA had over twice the risk of being prescribed/dispensed anti-asthmatics (RR= 2.20, 95% CI 2.10-2.30) compared to children born at 37+ weeks. The risk was also raised for reference children (RR=1.86, 95% CI 1.82-1.90). Relative risks decreased in children born 32-36 weeks GA but remained higher in children with congenital anomalies than in reference children (RR= 1.45, 95% CI 1.40-1.49; and RR= 1.31 95% CI 1.30-1.32 respectively). Boys were more likely to be prescribed anti-asthmatics than girls, with similar risks observed for children with congenital anomalies and reference children.

Congenital anomaly subgroups (children up to eight years of age)

The RR for having >1 anti-asthmatic prescription for children with specific congenital anomalies aged 0-7 years was compared to reference children. Children with oesophageal atresia had the highest risk of being prescribed/dispensed any anti-asthmatic medication (RR=3.57, 95% CI 3.14-4.06), inhaled corticosteroids (RR=5.46, 95% CI 4.46-6.68), and beta-2 agonists (RR=5.75, 95% CI 4.86-6.81), Supplementary Table 3. Children with genetic syndromes (Di George syndrome and Noonan syndrome), chromosomal anomalies (all chromosomal anomalies and Down syndrome with and without congenital heart defects), and diaphragmatic hernia had more than double the risk of having >1 prescription for any anti-asthmatics compared to reference children. Across all 32 congenital anomaly subgroups examined, children with these anomalies were significantly more likely to be prescribed any anti-asthmatic than reference children, except for children with Tetralogy of Fallot or children with hip dislocation and/or dysplasia whose risks were slightly raised but were not statistically significant.

The relative risk for receiving >1 prescription for beta-2-agonists and for inhaled corticosteroids for the 32 congenital anomaly subgroups followed the same trends as those for all anti-asthmatic medication, but they were less likely to be statistically significant due to smaller numbers being analysed.

For peer review only - http://bmjopen.bmj.com/site/about/guidelines.xhtml

2000-2004	Reference	
2005-2009	1.27 (1.23-1.31)	1.60 (1.59-1.61)
**Gestation Age**		
37+ weeks	Reference	
32-36 weeks	1.45 (1.40-1.49)	1.31 (1.30-1.32)
<32 weeks	2.20 (2.10-2.30)	1.86 (1.83-1.90)
**Sex of Child**		
Female	Reference	
Male	1.38 (1.35-1.41)	1.35 (1.35-1.36)

Table 2. Relative risk (RR) of >1 anti-asthmatic prescriptions in children with congenital anomalies and reference children up to age six years, by risk factor

For the birth cohort analysis, data were analysed only from three registries: Denmark: Funen, Finland, and Wales.

For peer review only

For peer review only - http://bmjopen.bmj.com/site/about/guidelines.xhtml

Sensitivity analysis

The overall RR of children with congenital anomalies receiving at least one prescription for any anti-asthmatic medication was 1.25 (95% CI 1.21-1.28) compared to reference children (Supplementary Table 4). This is compared to the RR = 1.41 (95% CI 1.35-1.48) of receiving >1 prescription. Although the risk estimates were lower for children with at least one prescription for any anti-asthmatic, the pattern of risk across registries and age groups was similar to that found for children with >1 prescription (Supplementary Figure 1).

For peer review only

For peer review only - http://bmjopen.bmj.com/site/about/guidelines.xhtml

Discussion

This population-based cohort study of over 1.78 million children in six European regions found that across all regions and age groups, children with congenital anomalies had a consistently higher risk of receiving prescriptions for >1 anti-asthmatic medication compared to reference children. We observed geographical variation in the use of anti-asthmatic medications with registries in Denmark, Italy and Spain having higher prevalence in young children whereas Finland and Wales had lower prevalence. By age 6-7 years, the prevalence in the individual registries started to converge for both children with and without anomalies, although it remained slightly higher for children with congenital anomalies. Children with oesophageal atresia, diaphragmatic hernia, chromosomal anomalies and specific genetic syndromes had the highest risk of being prescribed anti-asthmatic prescriptions. Male children, children born in a later cohort, and children born preterm had an increased risk of anti-asthmatic prescriptions, with children with congenital anomalies born very preterm (<32 weeks) having over double the risk of anti-asthmatic prescriptions compared to children with congenital anomalies born at term.

With the exception of Finland and Wales, our findings that anti-asthmatic prescriptions tended to be higher in younger age groups is consistent with literature indicating higher rates of wheezing and current wheeze in infants below two years of age than older children [8-9]. As beta-2 agonists and inhaled corticosteroids are recommended as treatment for infant wheeze [35], prescriptions for anti-asthmatics in young children may be indicative of wheeze and acute respiratory infections which can be common in young children [36]. However, other studies reported higher prevalence of breathing difficulties for older children in the form of parent-reported asthma [3]. It is possible that parental reports may overestimate asthma prevalence, while our study was based on administrative anti-asthmatic prescription records. We identified anti-asthmatic prevalence rates that seemed to vary from other regional studies. For example, a study based on a sample of Welsh children (n=1,529), born 2000-2002, with a GP diagnosis of asthma and/or prescriptions for anti-asthmatics in their health records in the last 12 months reported much higher prevalence rates ranging from 13.4% for 3 year olds to 10.4% in 7 year olds [38]. These differences may relate to methodology as our study criteria was >1 anti-asthmatic prescription in a year rather than at least one. We also identified regional differences within countries as within Italy, children in Emilia Romagna had a higher prevalence of >1 prescription for asthma medications than children in Tuscany. A study investigating the use of anti-asthmatics in women before, during and after pregnancy also reported this regional difference between the two Italian registries [37].

Children with congenital anomalies consistently had higher rates of anti-asthmatic medication than reference children which may be due to several factors. Congenital anomalies such as diaphragmatic hernia and Down syndrome are associated with pulmonary hypoplasia [26-29], requiring anti-asthmatics to manage wheezing episodes. Children with diaphragmatic hernia commonly require mechanical ventilation for days or weeks after birth which can lead to bronchopulmonary dysplasia [27; 30]. Beta-2 agonists are prescribed to children with bronchopulmonary dysplasia to reduce pulmonary resistance

For peer review only - http://bmjopen.bmj.com/site/about/guidelines.xhtml

[31]. Studies have shown that patients with diaphragmatic hernia were over six times more likely to have been diagnosed with asthma than the general population [30]. Congenital anomalies may be comorbid with respiratory conditions, such as the associations between oesophageal atresia, Down syndrome and Di George syndrome with respiratory tract disease [32-34]. We did not find an increased risk for prescription of beta-2 agonists for children with Tetralogy of Fallot. Beta-2-agonists should not be used in children with infundibular pulmonary stenosis as beta-blockers may be prescribed to prevent hypoxic spells in these children while awaiting surgery [39].

The finding that boys were more likely to be prescribed/dispensed anti-asthmatics is consistent with previous literature [10-15]. Both children with and without congenital anomalies had more anti-asthmatic prescriptions in 2005-2009 compared to 2000-2004. While self-reported anti-asthmatic use has increased in cohort studies of young adults between 1990-2007 [40], our findings support an increasing trend of anti-asthmatic prescriptions over time in children using administrative prescription data. Children born preterm at <32 weeks GA were twice as likely to be prescribed/dispensed anti-asthmatics than children born at term, although the risk was reduced for children born 32-36 weeks. The higher risk in children born <32 weeks GA may be due to respiratory complications such as respiratory distress syndrome or bronchopulmonary dysplasia arising from mechanical ventilation in preterm children [41-42].

A major strength of this study is that we have standardised data on congenital anomalies and a Common Data Model which enabled us to map and standardise the prescription/pharmacy dispensing records, as well as standardising demographic information on children. This is important as there are diverse coding classification systems, languages, and healthcare systems in Europe. Furthermore, this is the first population-based multi-centre study exploring anti-asthmatic prescriptions in children with and without congenital anomalies which included data from 60,662 children with congenital anomalies across Europe, overcoming limitations of previous research based on small sizes. The use of reference children for comparison in each geographical region is also a major strength, as some differences in prescription rates across Europe may be explained by methodological issues (such as frequency of prescriptions issued at the pharmacy) and different level of indication for prescribing asthma medications to small children. However, there were a number of limitations to this study. Firstly, bias may arise as a result of approximately 5% of children across registries being unable to be linked to the prescription database due to invalid identifications numbers or because their records were not sent to the national/vital statistics to be linked. In the case of Wales, the children were registered in a GP practice that was not part of the SAIL databank, therefore they could not be linked. Secondly, only three participating registries (Funen, Finland and Wales) could provide data for children up to and including nine years of age. Our list of anti-asthmatic medications did not include oral corticosteroids used to treat acute symptoms, but for young children this treatment may mainly be done during hospital stays, and we were unable to differentiate between long-acting and short-acting beta-2-agonists. While we explored medications prescribed and dispensed, we were unable to gauge whether these medications were actually taken.

For peer review only

For peer review only - http://bmjopen.bmj.com/site/about/guidelines.xhtml

In summary, this study demonstrates that valid information on the prevalence of anti-asthmatic prescriptions for children can be obtained through data linkage studies in order to monitor regional differences across age groups and over time. In general, children with congenital anomalies had a higher prevalence and risk of anti-asthmatic prescriptions than reference children. To evaluate the additional burden of disease that children with congenital anomalies face, it is essential to compare these children to those without congenital anomalies in the same geographic areas as prescription patterns of anti-asthmatic medications vary across Europe. The findings from this study are also beneficial to clinicians for identifying which congenital anomalies are associated with a higher risk of prescriptions for respiratory symptoms and breathing difficulties.

For peer review only

For peer review only - http://bmjopen.bmj.com/site/about/guidelines.xhtml

Contributorship Statement: ND prepared the manuscript and revised it following feedback and comments from all authors. ND analysed the pooled data from each registry. JG and ML produced the standardisation scripts. JT and JKM produced the central analysis scripts. GA, EB, LBB, CC, AC, MG, AH, SJ, AP, IS, SKU extracted and analysed local registry data. ML, EG and JKM secured funding, conceptualised and designed the study, and provided frequent feedback on manuscript drafts. JKM and ML provided data analysis guidance and syntax for meta-analyses. ML executed data analysis syntax relating to protected data.

Competing Interests: None

Funding Statement: This work was supported by the European Union's Horizon 2020 research and innovation programme under grant agreement No. 733001. The views presented here are those of the authors only, and the European Commission is not responsible for any use that may be made of the information presented here.

Data Sharing Statement: No additional data available.

Ethics Approval Statement: Each participating registry obtained local approvals for their data to be used in the EUROlinkCAT project. Ethical approval for this study was obtained from Ulster University's Nursing and Health Research Ethics Filter Committee (Reference Number: FCNUR-21-060).

For peer review only

For peer review only - http://bmjopen.bmj.com/site/about/guidelines.xhtml

References

1. World Health Organisation. Asthma [Internet]. World Health Organisation; 2021 [cited 2021 Aug 18]. Available from: https://www.who.int/news-room/fact-sheets/detail/asthma
2. Akinbami LJ, Simon AE, Rossen LM. Changing trends in asthma prevalence among children. Pediatrics. 2016 Jan 1;137(1).
3. Wirl C, Puklova V. Prevalence of asthma and allergies in children. *European Environment and Health Information System Fact Sheet, World Health Organization. 2007*; Fact Sheet No 3.1 (May).
4. Pearce N, Aït-Khaled N, Beasley R, Mallol J, Keil U, Mitchell E, Robertson C. Worldwide trends in the prevalence of asthma symptoms: phase III of the International Study of Asthma and Allergies in Childhood (ISAAC). Thorax. 2007 Sep 1;62(9):758-66.
5. Uphoff EP, Bird PK, Antó JM, Basterrechea M, Von Berg A, Bergström A, Bousquet J, Chatzi L, Fantini MP, Ferrero A, Gehring U. Variations in the prevalence of childhood asthma and wheeze in MeDALL cohorts in Europe. ERJ open research. 2017 Jul 1;3(3).
6. Bloom CI, Saglani S, Feary J, Jarvis D, Quint JK. Changing prevalence of current asthma and inhaled corticosteroid treatment in the UK: population-based cohort 2006–2016. European Respiratory Journal. 2019 Apr 1;53(4).
7. Sestini P, De Sario M, Bugiani M, Bisanti L, Giannella G, Kaisermann D, Frasca G, Lombardi E, Petronio MG, Dell'Orco V, Indinnimeo L. Frequency of asthma and allergies in Italian children and adolescents: results from SIDRIA-2. Epidemiologia e prevenzione. 2005 Mar 1;29(2 Suppl):24-31
8. Alvarez-Alvarez I, Niu H, Guillen-Grima F, Aguinaga-Ontoso I. Meta-analysis of prevalence of wheezing and recurrent wheezing in infants. Allergologia et immunopathologia. 2018 May 1;46(3):210-7.
9. Lai CK, Beasley R, Crane J, Foliaki S, Shah J, Weiland S, ISAAC Phase Three Study Group. Global variation in the prevalence and severity of asthma symptoms: phase three of the International Study of Asthma and Allergies in Childhood (ISAAC). Thorax. 2009 Jun 1;64(6):476-83.
10. Duggan EM, Sturley J, Fitzgerald AP, Perry IJ, Hourihane JO. The 2002–2007 trends of prevalence of asthma, allergic rhinitis and eczema in Irish schoolchildren. Pediatric allergy and immunology. 2012 Aug;23(5):464-71.
11. Ciccone G, Camarlengo A, Bugiani M, Dalmasso P, Faggiano F, Volante T, Magnani C, Natale P, Piccioni P, Bisanti L, Gianelle V. Asthma and respiratory symptoms in 6-7 yr old Italian children: gender, latitude, urbanization and socioeconomic factors. Eur Respir J. 1997 Aug 1;10:1780-6.
12. Linneberg A, Simonsen JB, Petersen J, Stensballe LG, Benn CS. Differential effects of risk factors on infant wheeze and atopic dermatitis emphasize a different etiology. Journal of Allergy and Clinical Immunology. 2006 Jan 1;117(1):184-9.
13. Henriksen L, Simonsen J, Haerskjold A, Linder M, Kieler H, Thomsen SF, Stensballe LG. Incidence rates of atopic dermatitis, asthma, and allergic rhinoconjunctivitis in

For peer review only - http://bmjopen.bmj.com/site/about/guidelines.xhtml

Danish and Swedish children. Journal of Allergy and Clinical Immunology. 2015 Aug 1;136(2):360-6.

14. Hoffmann F, Glaeske G. Prescriptions as a proxy for asthma in children: a good choice?. European journal of clinical pharmacology. 2010 Mar;66(3):307-13.
15. Stock S, Redaelli M, Luengen M, Wendland G, Civello D, Lauterbach KW. Asthma: prevalence and cost of illness. European Respiratory Journal. 2005 Jan 1;25(1):47-53.
16. Dik N, Tate RB, Manfreda J, Anthonisen NR. Risk of physician-diagnosed asthma in the first 6 years of life. Chest. 2004 Oct 1;126(4):1147-53.
17. Benoist G, Mokhtari M, Deschildre A, Khen-Dunlop N, Storme L, Benachi A, Delacourt C. Risk of readmission for wheezing during infancy in children with congenital diaphragmatic hernia. PloS one. 2016 May 12;11(5):e0155556
18. Nassif M, van Steenwijk RP, Hogenhout JM, Lu H, de Bruin-Bon RH, Hirsch A, Sterk PJ, Bouma BJ, Straver B, Tijssen JG, Mulder BJ. Atrial septal defect in adults is associated with airway hyperresponsiveness. Congenital heart disease. 2018 Nov;13(6):959-66.
19. Agrawal L, Beardsmore CS, MacFadyen UM. Respiratory function in childhood following repair of oesophageal atresia and tracheoesophageal fistula. Archives of disease in childhood. 1999 Nov 1;81(5):404-8.
20. Boyd PA, Haeusler M, Barisic I, Loane M, Garne E, Dolk H. Paper 1: The EUROCAT network—organization and processes. *Birth Defects Research Part A, Clinical and Molecular Teratology.* 2011;91(S1):S2-S15.
21. Morris JK, Garne E, Loane M, Barisic I, Densem J, Latos-Bieleńska A, et al. EUROlinkCAT protocol for a European population-based data linkage study investigating the survival, morbidity and education of children with congenital anomalies. BMJ Open. 2021;11(6):e047859.
22. Loane M, Given JE, Tan J, Astolfi G, Barisic I, Carbonell CC, et al. (2021) Creating a population-based cohort of children born with and without congenital anomalies using birth data linked to hospital discharge databases in 11 European regions: assessment of linkage success and data quality. PLoS ONE (submitted)
23. EUROCAT (2020). EUROCAT Guide 1.4: instruction for the registration of congenital anomalies. EUROCAT Central Registry, Ulster University
24. DerSimonian R, Laird N. Meta-analysis in clinical trials. Control Clin Trials. 1986; 7(3):177–88. https://doi.org/10.1016/0197-2456(86)90046-2.
25. StataCorp. 2021. *Stata Statistical Software: Release 17*. College Station, TX: StataCorp LLC.
26. Cooney TP, Thurlbeck WM. Pulmonary hypoplasia in Down's syndrome. New England Journal of Medicine. 1982 Nov 4;307(19):1170-3.
27. Ijsselstijn H, Tibboel D, Hop WJ, Molenaar JC, de Jongste JC. Long-term pulmonary sequelae in children with congenital diaphragmatic hernia. Am J Respir Crit Care Med 1997;155:174–180.
28. Logan JW, Rice HE, Goldberg RN, Cotten CM. Congenital diaphragmatic hernia: a systematic review and summary of best-evidence practice strategies. Journal of Perinatology. 2007 Sep;27(9):535-49.

For peer review only - http://bmjopen.bmj.com/site/about/guidelines.xhtml

29. Wang J, Liu H, Gao L, Liu X. Impaired FGF10 signaling and epithelial development in experimental lung hypoplasia with esophageal atresia. Frontiers in pediatrics. 2018 Apr 20;6:109.
30. Spoel M, van der Cammen-van Zijp MH, Hop WC, Tibboel D, de Jongste JC, IJsselstijn H. Lung function in young adults with congenital diaphragmatic hernia; a longitudinal evaluation. Pediatric pulmonology. 2013 Feb;48(2):130-7.
31. Pfenninger J, Aebi C. Respiratory response to salbutamol (albuterol) in ventilator-dependent infants with chronic lung disease: pressurized aerosol delivery versus intravenous injection. Intensive care medicine. 1993 May;19(5):251-5.
32. Usui N, Kamata S, Ishikawa S, Sawai T, Okuyama H, Imura K, Okada A. Anomalies of the tracheobronchial tree in patients with esophageal atresia. Journal of pediatric surgery. 1996 Feb 1;31(2):258-62.
33. Verheij E, Speleman L, van der Molen AB, Thomeer HG. Congenital respiratory tract disorders in 22q11. 2 deletion syndrome. International journal of pediatric otorhinolaryngology. 2018 Jan 1;104:1-4.
34. Colvin KL, Yeager ME. What people with Down Syndrome can teach us about cardiopulmonary disease. European Respiratory Review. 2017 Mar 31;26(143).
35. Brand PL, Baraldi E, Bisgaard H, Boner AL, Castro-Rodriguez JA, Custovic A, de Blic J, De Jongste JC, Eber E, Everard ML, Frey U. Definition, assessment and treatment of wheezing disorders in preschool children: an evidence-based approach. European Respiratory Journal. 2008 Oct 1;32(4):1096-110.
36. Nair H, Nokes DJ, Gessner BD, Dherani M, Madhi SA, Singleton RJ, O'Brien KL, Roca A, Wright PF, Bruce N, Chandran A. Global burden of acute lower respiratory infections due to respiratory syncytial virus in young children: a systematic review and meta-analysis. The Lancet. 2010 May 1;375(9725):1545-55.
37. Charlton RA, Pierini A, Klungsøyr K, Neville AJ, Jordan S, Thayer D, Bos HJ, Puccini A, Hansen AV, Gini R, Engeland A. Asthma medication prescribing before, during and after pregnancy: a study in seven European regions. BMJ open. 2016 Jan 1;6(1):e009237.
38. Griffiths LJ, Lyons RA, Bandyopadhyay A, et al. Childhood asthma prevalence: cross-sectional record linkage study comparing parent-reported wheeze with general practitioner-recorded asthma diagnoses from primary care electronic health records in Wales. BMJ Open Resp Res 2018;5:e000260. doi:10.1136/ bmjresp-2017-000260
39. Fanous E, Mogyorósy G. Does the prophylactic and therapeutic use of beta-blockers in preoperative patients with tetralogy of Fallot significantly prevent and treat the occurrence of cyanotic spells? Interactive cardiovascular and thoracic surgery. 2017 Oct 1;25(4):647-50.
40. Sigurkarlsson S, Clausen M, Gislason T, Gislason D. Prevalence of respiratory symptoms and use of asthma drugs are increasing among young adult Icelanders. Laeknabladid. 2011 Sep 1;97(9):463-7.
41. Fraser J, Walls M, McGuire W. Respiratory complications of preterm birth. Bmj. 2004 Oct 21;329(7472):962-5.

For peer review only - http://bmjopen.bmj.com/site/about/guidelines.xhtml

42. Xu YP. Bronchopulmonary dysplasia in preterm infants born at less than 32 weeks gestation. Global pediatric health. 2016 Sep 13;3:2333794X16668773.

For peer review only

For peer review only - http://bmjopen.bmj.com/site/about/guidelines.xhtml

Legends

Figure 1. Prevalence of >1 prescription for any anti-asthmatic per 100 children in each age group, by registry

Figure 2. Relative risk (RR) of >1 prescription for any anti-asthmatic (AA) in children with congenital anomalies (CAs) compared to reference children, by registry
CI=Confidence intervals,
DL=DerSimonian-Laird

Figure 3. Relative risk (RR) of >1 beta-2 agonist (B2A) prescription and >1 inhaled corticosteroid (ICS) prescription in children with congenital anomalies (CAs) compared to reference children, by registry
CI=Confidence interval
DL=DerSimonian-Laird

For peer review only

For peer review only - http://bmjopen.bmj.com/site/about/guidelines.xhtml

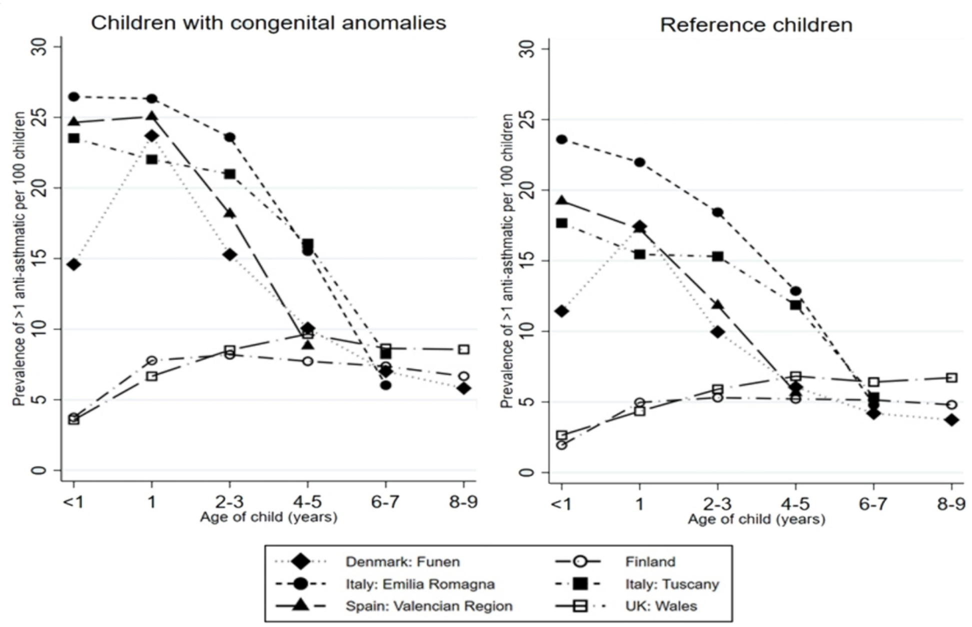

Figure 1. Prevalence of >1 prescription for any anti-asthmatic per 100 children in each age group, by registry

582x366mm (72 x 72 DPI)

Figure 2. Relative risk (RR) of >1 prescription for any anti-asthmatic (AA) in children with congenital anomalies (CAs) compared to reference children, by registry
CI=Confidence intervals,
DL=DerSimonian-Laird

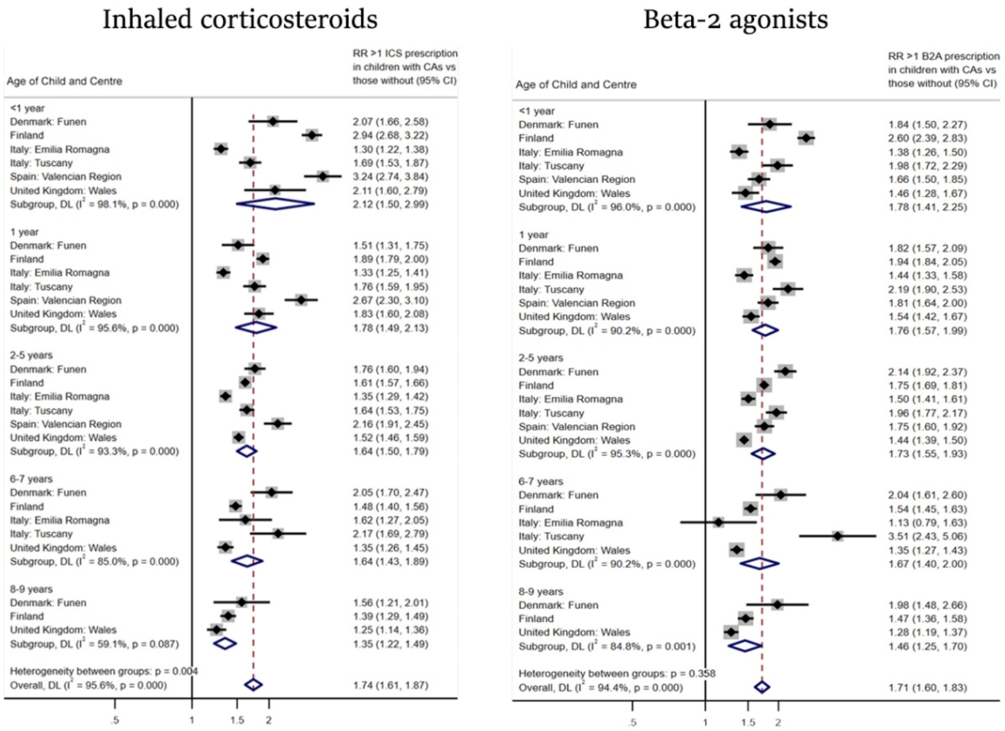

Figure 3. Relative risk (RR) of >1 beta-2 agonist (B2A) prescription and >1 inhaled corticosteroid (ICS) prescription in children with congenital anomalies (CAs) compared to reference children, by registry
CI=Confidence interval
DL=DerSimonian-Laird

426x318mm (72 x 72 DPI)

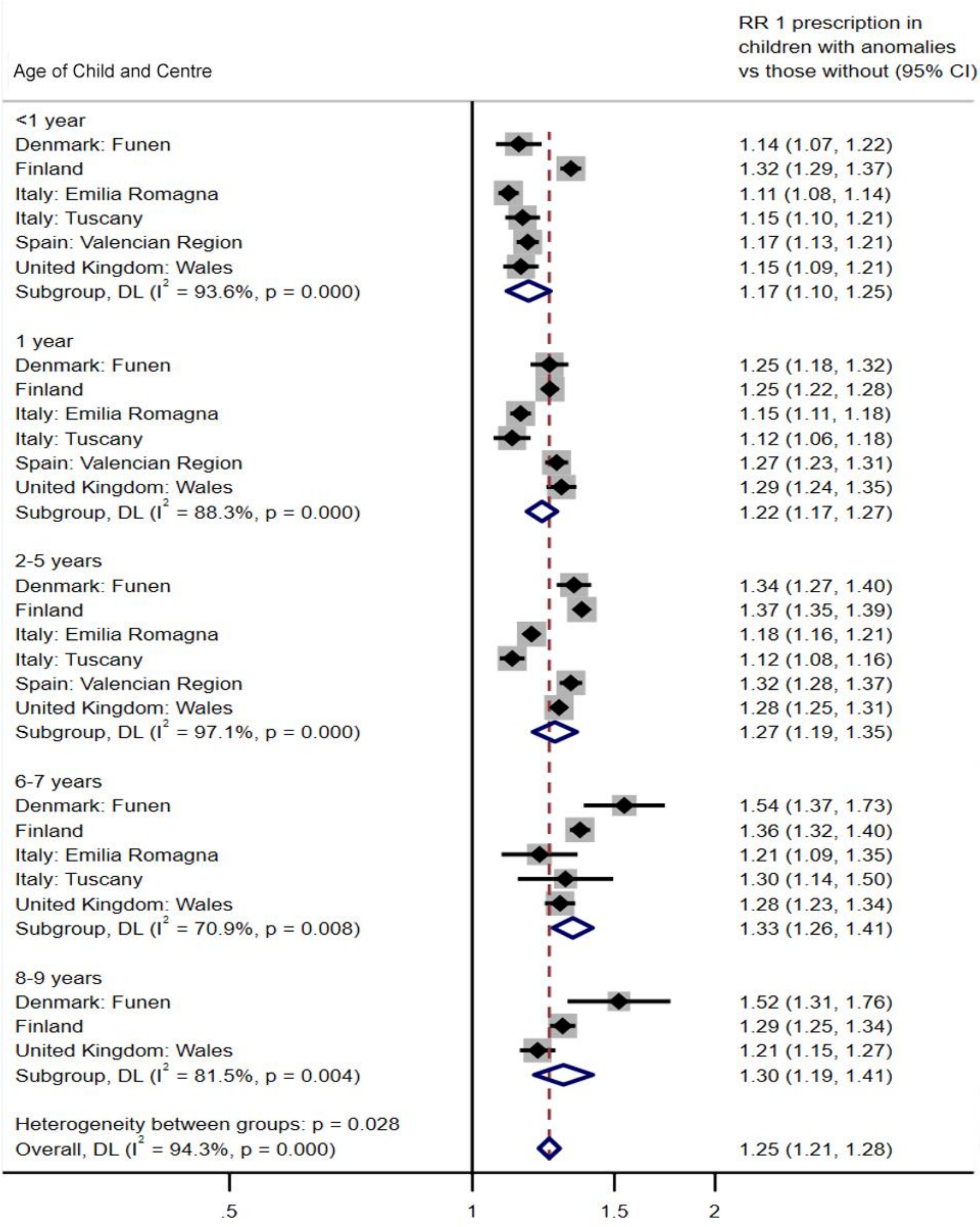

Supplementary Figure 1. Relative risk (RR) of at least 1 prescription for any anti-asthmatic in children with congenital anomalies (CAs) compared to reference children, by registry

CI=Confidence interval,

DL=DerSimonian-Laird

Registry	Birth years included	Total number of children in population		Number of children included in the study		Number of children not linked*	
		Children with congenital anomalies	Reference children	Children with congenital anomalies (%)	Reference children (%)	Children with congenital anomalies (%)	Reference children (%)
**Denmark: Funen**	2000-2014	1,813	72,382	1,789 (98.7)	72,290 (99.9)	24 (1.3)	92 (0.1)
**Finland**	2000-2014	33,177	762,989	32,926 (99.2)	755,923 (99.1)	251 (0.8)	7,066 (0.9)
**Italy: Emilia Romagna**	2008-2014	5,801	263,574	5,499 (94.8)	250,829 (95.2)	302 (5.2)	12,745 (4.8)
**Italy: Tuscany**	2008-2014	3,445	16,844	3,048 (88.5)	16,844 (100)	397 (11.5)	0
**Spain: Valencian Region**	2010-2014	4,308	223,760	4,281 (99.4)	223,760 (100)	27 (0.6)	0
**UK: Wales**	2000-2014	15,367	471,882	13,119 (85.4)	403,266 (85.5)	2,248 (14.6)	68,616 (14.5)
**Total**		63,911	1,811,431	60,662 (94.9)	1,722,912 (95.1)	3,249 (5.1)	88,519 (4.9)

*These children were excluded from the study

Supplementary Table 1. Number and percentage of children included in the study by registry

For peer review only

Type of Anomaly*	Congenital Anomaly	ICD10-BPA Code	ICD9 Code
Isolated*	Spina bifida	Q05	741
	Hydrocephalus	Q03	7423
	Anomalies of the corpus callosum	Q040	74221
	Severe microcephaly	Q02	7421
	Congenital heart defects (CHD)**	Q20-Q26	745, 746, 7470-7474
	Severe CHD***	Q200, Q201, Q203, Q204, Q212, Q213, Q220, Q224, Q225, Q226, Q230, Q232, Q233, Q234, Q251, Q252, Q262	74500, 74510, 7452, 7453, 7456, 7461, 7462, 74600, 7463, 7465, 7466, 7467, 7471, 74720, 74742
	Transposition of great arteries	Q203	74510
	Ventricular septal defect	Q210	7454
	Atrial septal defect	Q211	7455
	Tetralogy of Fallot	Q213	7452
	Coarctation of the aorta	Q251	7471
	Patent ductus arteriosus	Q250	7470
	Cleft lip (with or without cleft palate)	Q36, Q37	7491, 7492
	Cleft palate	Q35	7490
	Oesophageal atresia	Q390-Q391	75030-75031
	Anorectal atresia	Q420-Q423	75121-75124
	Diaphragmatic hernia	Q790	75661
	Gastroschisis	Q793	75671
	Multicystic renal dysplasia	Q6140, Q6141	75316
	Congenital hydronephrosis	Q620	75320
	Hypospadias	Q54	75260
	Limb reduction defects	Q71-Q73	7552-7554
	Club foot	Q660	75450
	Hip dislocation and/or dysplasia	Q650-Q652, Q6580, Q6581	75430
	Craniosynostosis	Q750	75600
Chromosomal	Down syndrome	Q90	7580
	Down syndrome without CHD	Q90 excluding codes Q20-Q26	7580 excluding codes 745, 746, 7470-7474

For peer review only

	Down syndrome with CHD	Q90 including codes Q20-Q26	7580 including codes 745, 746, 7470-7474
	Turner syndrome	Q96	75860, 75861, 75862, 75869
Genetic syndromes	Di George syndrome	D821	27910
	Noonan syndrome	Q8714	759896

Supplementary Table 2. Congenital anomalies and relevant International Classification of Diseases (ICD)-British Paediatric Association (BPA) codes

*Based on the EUROCAT algorithm [13]

**CHD includes all the anomalies listed under severe CHD, see below, as well as atrial septal defect, ventricular septal defect, pulmonary valve stenosis and patent ductus arteriosus in term infants

***Severe CHD included the following CHD subgroups: common arterial truncus, double outlet right ventricle, transposition of great vessels, single ventricle, atrioventricular septal defect, tetralogy of Fallot, pulmonary valve atresia, tricuspid atresia and stenosis, Ebstein anomaly, hypoplastic right heart, aortic valve atresia/stenosis, mitral valve anomalies, hypoplastic left heart, coarctation of aorta, aortic atresia/interrupted aortic arch, total anomalous pulmonary venous return.

For peer review only

Isolated Anomaly	Any Anti-Asthmatic (RR, 95% CI)	Beta-2 Agonists (RR, 95% CI)	Inhaled Corticosteroids (RR, 95% CI)
**Spina bifida** n = 154	1.68 (1.35-2.09)	2.53 (1.89-3.38)	2.52 (1.97-3.23)
**Hydrocephalus** n = 299	1.59 (1.41-1.80)	2.24 (1.86-2.69)	2.04 (1.71-2.45)
**Severe microcephaly** n = 278	1.53 (1.30-1.79)	2.15 (1.63-2.84)	1.82 (1.44-2.31)
**Anomalies of corpus callosum** n = 471	1.94 (1.70-2.22)	3.44 (2.90-4.08)	2.90 (2.53-3.33)
**Congenital heart defects (CHD)** n = 19,643	1.31 (1.27-1.36)	1.47 (1.40-1.54)	1.48 (1.39-1.57)
**Severe CHD** n = 3,368	1.42 (1.33-1.52)	1.60 (1.47-1.74)	1.83 (1.65-2.02)
**Transposition of great arteries** n = 488	1.46 (1.28-1.66)	1.91 (1.62-2.24)	2.10 (1.81-2.43)
**Ventricular septal defect** n = 11,826	1.21 (1.18-1.24)	1.35 (1.30-1.41)	1.26 (1.20-1.31)
**Atrial septal defect** n = 2,679	1.43 (1.33-1.54)	1.51 (1.37-1.67)	1.53 (1.37-1.70)
**Tetralogy of Fallot** n = 426	1.10 (0.97-1.25)	1.19 (0.94-1.49)	1.52 (1.26-1.84)
**Coarctation of aorta** n = 1,021	1.72 (1.56-1.90)	2.09 (1.85-2.36)	2.12 (1.88-2.38)
**Patent ductus arteriosus** n = 385	1.57 (1.40-1.76)	2.24 (1.81-2.77)	2.23 (1.84-2.71)
**Cleft lip (with or without cleft palate)** n = 1,299	1.27 (1.19-1.36)	1.62 (1.47-1.80)	1.31 (1.18-1.45)
**Cleft palate** n = 1,164	1.49 (1.35-1.65)	1.79 (1.49-2.15)	1.71 (1.49-1.96)
**Oesophageal atresia** n = 239	3.57 (3.14-4.06)	5.75 (4.86-6.81)	5.46 (4.46-6.68)
**Anorectal atresia** n = 236	1.40 (1.20-1.64)	1.92 (1.48-2.49)	1.82 (1.45-2.28)
**Diaphragmatic hernia** n = 211	2.28 (1.72-3.03)	3.70 (2.85-4.81)	3.34 (2.48-4.50)
**Gastroschisis** n = 376	1.45 (1.27-1.67)	1.85 (1.52-2.25)	1.44 (1.15-1.80)

**Multicystic renal dysplasia** n = 621	1.31 (1.18-1.46)	1.83 (1.58-2.11)	1.56 (1.33-1.83)
**Congenital hydronephrosis** n = 3,026	1.21 (1.15-1.28)	1.33 (1.20-1.46)	1.27 (1.18-1.38)
**Hypospadias** n = 2,526	1.30 (1.22-1.39)	1.52 (1.37-1.69)	1.44 (1.32-1.57)
**Limb reduction defects** n = 445	1.16 (1.03-1.32)	1.40 (1.14-1.72)	1.32 (1.06-1.64)
**Club foot** n = 1,997	1.31 (1.24-1.38)	1.46 (1.34-1.59)	1.42 (1.31-1.54)
**Hip dislocation and/or dysplasia** n = 1,026	1.05 (0.96-1.15)	1.03 (0.91-1.17)	1.22 (1.07-1.39)
**Craniosynostosis** n = 765	1.37 (1.26-1.49)	1.70 (1.50-1.93)	1.46 (1.27-1.67)
**All chromosomal** n = 3,608	2.19 (2.00-2.40)	2.86 (2.47-3.31)	2.94 (2.58-3.34)
**Down syndrome** n = 1,993	2.19 (2.00-2.40)	2.77 (2.40-3.20)	2.88 (2.54-3.28)
**Down syndrome (including CHD)** n = 1,131	2.33 (2.09-2.61)	2.93 (2.47-3.48)	3.12 (2.70-3.61)
**Down syndrome (excluding CHD)** n = 862	2.11 (1.93-2.31)	2.76 (2.39-3.19)	2.73 (2.31-3.23)
**Turner syndrome** n = 143	1.48 (1.21-1.82)	2.50 (1.91-3.28)	2.15 (1.64-2.82)
**Di George syndrome** n = 172	3.47 (3.04-3.95)	5.11 (4.35-6.01)	4.88 (4.01-5.94)
**Noonan syndrome** n = 120	2.33 (1.99-2.72)	2.76 (2.09-3.64)	3.68 (2.82-4.82)

Supplementary Table 3. Combined relative risks (RR) across registries for >1 anti-asthmatic prescriptions up to age eight years, by anomaly

	RR of receiving ≥1 prescription for any anti-asthmatic compared to reference children	RR of receiving >1 prescription for any anti-asthmatic compared to reference children
**<1 year**	1.17 (1.10-1.25)	1.39 (1.17-1.65)
**1 year**	1.22 (1.17-1.27)	1.41 (1.29-1.55)
**2-5 years**	1.27 (1.19-1.35)	1.44 (1.35-1.53)
**6-7 years**	1.33 (1.26-1.41)	1.43 (1.32-1.54)
**8-9 years**	1.30 (1.19-1.41)	1.37 (1.25-1.51)
**All years combined**	1.25 (1.21-1.28)	1.41 (1.35-1.48)

Supplementary Table 4. Relative risk (RR) of receiving ≥1 prescription versus receiving >1 prescription for any anti-asthmatic in children with congenital anomalies compared to reference children

For peer review only

Reporting checklist for cohort study.

Based on the STROBE cohort guidelines.

Instructions to authors

Complete this checklist by entering the page numbers from your manuscript where readers will find each of the items listed below.

Your article may not currently address all the items on the checklist. Please modify your text to include the missing information. If you are certain that an item does not apply, please write "n/a" and provide a short explanation.

Upload your completed checklist as an extra file when you submit to a journal.

In your methods section, say that you used the STROBE cohortreporting guidelines, and cite them as:

von Elm E, Altman DG, Egger M, Pocock SJ, Gotzsche PC, Vandenbroucke JP. The Strengthening the Reporting of Observational Studies in Epidemiology (STROBE) Statement: guidelines for reporting observational studies.

		Reporting Item	Page Number
**Title and abstract**			
Title	#1a	Indicate the study's design with a commonly used term in the title or the abstract	1
Abstract	#1b	Provide in the abstract an informative and balanced summary of what was done and what was found	1
**Introduction**			
Background / rationale	#2	Explain the scientific background and rationale for the investigation being reported	3
Objectives	#3	State specific objectives, including any prespecified hypotheses	3
**Methods**			
Study design	#4	Present key elements of study design early in the paper	4
Setting	#5	Describe the setting, locations, and relevant dates, including periods	4

For peer review only

		of recruitment, exposure, follow-up, and data collection	
Eligibility criteria	#6a	Give the eligibility criteria, and the sources and methods of selection of participants. Describe methods of follow-up.	4
Eligibility criteria	#6b	For matched studies, give matching criteria and number of exposed and unexposed	4
Variables	#7	Clearly define all outcomes, exposures, predictors, potential confounders, and effect modifiers. Give diagnostic criteria, if applicable	4
Data sources / measurement	#8	For each variable of interest give sources of data and details of methods of assessment (measurement). Describe comparability of assessment methods if there is more than one group. Give information separately for for exposed and unexposed groups if applicable.	4-5
Bias	#9	Describe any efforts to address potential sources of bias	10
Study size	#10	Explain how the study size was arrived at	6
Quantitative variables	#11	Explain how quantitative variables were handled in the analyses. If applicable, describe which groupings were chosen, and why	5
Statistical methods	#12a	Describe all statistical methods, including those used to control for confounding	
5			
Statistical methods	#12b	Describe any methods used to examine subgroups and interactions	5
Statistical methods	#12c	Explain how missing data were addressed	6
Statistical methods	#12d	If applicable, explain how loss to follow-up was addressed	N/A
Statistical methods	#12e	Describe any sensitivity analyses	
8			
**Results**			
Participants	#13a	Report numbers of individuals at each stage of study—eg numbers potentially eligible, examined for eligibility, confirmed eligible,	6

For peer review only

		included in the study, completing follow-up, and analysed. Give information separately for for exposed and unexposed groups if applicable.	
Participants	#13b	Give reasons for non-participation at each stage	6
Participants	#13c	Consider use of a flow diagram	
N/A			
Descriptive data	#14a	Give characteristics of study participants (eg demographic, clinical, social) and information on exposures and potential confounders. Give information separately for exposed and unexposed groups if applicable.	6
Descriptive data	#14b	Indicate number of participants with missing data for each variable of interest	
6, Supplementary Table 1			
Descriptive data	#14c	Summarise follow-up time (eg, average and total amount)	
17			
Outcome data	#15	Report numbers of outcome events or summary measures over time. Give information separately for exposed and unexposed groups if applicable.	
17			
Main results	#16a	Give unadjusted estimates and, if applicable, confounder-adjusted estimates and their precision (eg, 95% confidence interval). Make clear which confounders were adjusted for and why they were included	6-7
Main results	#16b	Report category boundaries when continuous variables were categorized	N/A
Main results	#16c	If relevant, consider translating estimates of relative risk into absolute risk for a meaningful time period	
N/A			
Other analyses	#17	Report other analyses done—eg analyses of subgroups and interactions, and sensitivity analyses	8

For peer review only

Discussion

Key results	#18	Summarise key results with reference to study objectives	9-10
Limitations	#19	Discuss limitations of the study, taking into account sources of potential bias or imprecision. Discuss both direction and magnitude of any potential bias.	10
Interpretation	#20	Give a cautious overall interpretation considering objectives, limitations, multiplicity of analyses, results from similar studies, and other relevant evidence.	10-11
Generalisability	#21	Discuss the generalisability (external validity) of the study results	10-11
**Other Information**			
Funding	#22	Give the source of funding and the role of the funders for the present study and, if applicable, for the original study on which the present article is based	1

Notes:

- 14b: 6, Supplementary Table 1 The STROBE checklist is distributed under the terms of the Creative Commons Attribution License CC-BY. This checklist was completed on 03. October 2022 using https://www.goodreports.org/, a tool made by the EQUATOR Network in collaboration with Penelope.ai

For peer review only

BMJ Open

Anti-asthmatic prescriptions in children with and without congenital anomalies: a population-based study

Journal:	*BMJ Open*
Manuscript ID	bmjopen-2022-068885.R1
Article Type:	Original research
Date Submitted by the Author:	24-May-2023
Complete List of Authors:	Divin, Natalie; Ulster University Faculty of Life and Health Sciences, Institute of Nursing and Health Research<br>Given, Joanne; Ulster University Faculty of Life and Health Sciences, Institute of Nursing and Health Research<br>Tan, Joachim; St George's University of London, Population Health Research Institute<br>Astolfi, Gianni; University of Ferrara, Department of Neuroscience and Rehabilitation<br>Ballardini, Elisa; University of Ferrara, Italy, Neonatal Intensive Care Unit, Paediatric Section, University of Ferrara, Italy<br>Barrachina-Bonet, Laia; Foundation for the Promotion of Health and Biomedical Research in the Valencian region, Rare Disease Research Unit<br>Cavero- Carbonell, Clara; Foundation for the Promotion of Health and Biomedical Research in the Valencian region, Rare Diseases Research Unit<br>Coi, Alessio; National Research Council Pisa Research Area, Institute of Clinical Physiology<br>Garne, Ester; University of Southern Denmark<br>Gissler, Mika; THL National Institute for Health and Welfare, Information<br>Heino, Anna; Finnish Institute for Health and Welfare<br>Jordan, Susan; Swansea University, Nursing<br>Pierini, Anna; Institute of Clinical Physiology National Research Council<br>Scanlon, Ieuan; Swansea University<br>Urhøj, Stine Kjær ; University of Copenhagen, Section of Epidemiology, Department of Public Health<br>Morris, Joan; St George's, University of London, Population Health Research Institute<br>Loane , Maria ; Ulster University Faculty of Life and Health Sciences, Institute of Nursing and Health Research
**Primary Subject Heading**:	Epidemiology
Secondary Subject Heading:	Paediatrics
Keywords:	RESPIRATORY MEDICINE (see Thoracic Medicine), PAEDIATRICS, EPIDEMIOLOGY

SCHOLARONE™
Manuscripts

I, the Submitting Author has the right to grant and does grant on behalf of all authors of the Work (as defined in the below author licence), an exclusive licence and/or a non-exclusive licence for contributions from authors who are: i) UK Crown employees; ii) where BMJ has agreed a CC-BY licence shall apply, and/or iii) in accordance with the terms applicable for US Federal Government officers or employees acting as part of their official duties; on a worldwide, perpetual, irrevocable, royalty-free basis to BMJ Publishing Group Ltd ("BMJ") its licensees and where the relevant Journal is co-owned by BMJ to the co-owners of the Journal, to publish the Work in this journal and any other BMJ products and to exploit all rights, as set out in our licence.

The Submitting Author accepts and understands that any supply made under these terms is made by BMJ to the Submitting Author unless you are acting as an employee on behalf of your employer or a postgraduate student of an affiliated institution which is paying any applicable article publishing charge ("APC") for Open Access articles. Where the Submitting Author wishes to make the Work available on an Open Access basis (and intends to pay the relevant APC), the terms of reuse of such Open Access shall be governed by a Creative Commons licence – details of these licences and which Creative Commons licence will apply to this Work are set out in our licence referred to above.

Other than as permitted in any relevant BMJ Author's Self Archiving Policies, I confirm this Work has not been accepted for publication elsewhere, is not being considered for publication elsewhere and does not duplicate material already published. I confirm all authors consent to publication of this Work and authorise the granting of this licence.

For peer review only

Anti-asthmatic prescriptions in children with and without congenital anomalies: a population-based study

For peer review only

Authors: Natalie Divin PhD[1], Joanne Given PhD[1], Joachim Tan PhD[2], Gianni Astolfi BSc[3], Elisa Ballardini MD[3], Laia Barrachina Bonet MSc[4], Clara Cavero PhD[4], Alessio Coi PhD[5], Ester Garne MD[6], Mika Gissler PhD[7], Anna Heino M.Soc.Sc[7], Susan Jordan PhD[8], Anna Pierini BSc[5], Ieuan Scanlon MSc[8], Stine Kjaer Urhoj PhD[9], Joan K Morris PhD[2], Maria Loane PhD[1]

Affiliations: [1]Ulster University; [2]St George's University of London; [3]Department of Neuroscience and Rehabilitation, University of Ferrara; [4]Rare Diseases Research Unit, Foundation for the Promotion of Health and Biomedical Research of Valencia Region; [5]Unit of Epidemiology of Rare Diseases and Congenital Anomalies, Institute of Clinical Physiology; [6]Pediatric Department, Hospital Lillebaelt Kolding; [7]Finnish Institute for Health and Welfare (THL); [8]Swansea University; [9]University of Copenhagen.

Word count: 3,792

Corresponding author:
Maria Loane
Ulster University, Belfast Campus, Room BC-04-167
York Street,
Belfast, Co Antrim
N Ireland BT15 1ES
Telephone: +44 2895 365256
Email: ma.loane@ulster.ac.uk

Abstract

Objectives: To explore the risk of being prescribed/dispensed medications for respiratory symptoms and breathing difficulties in children with and without congenital anomalies.

Design: A EUROlinkCAT population-based data linkage cohort study. Data on children with and without congenital anomalies were linked to prescription databases to identify children who did/did not receive anti-asthmatic prescriptions. Data were analysed by age, European region, class of anti-asthmatic, anomaly, sex, gestational age, and birth cohort.

Setting: Children born 2000-2014 in six regions within five European countries.

Participants: 60,662 children with congenital anomalies and 1,722,912 reference children up to age ten years.

Primary outcome measure: Relative risks (RR) of >1 anti-asthmatic prescription in a year, identified using Anatomical Therapeutic Chemical classification codes beginning with R03.

Results: There were significant differences in the prescribing of anti-asthmatics in the six regions. Children with congenital anomalies had a significantly higher risk of being prescribed anti-asthmatics (RR=1.41, 95% CI 1.35-1.48) compared to reference children. The increased risk was consistent across all regions and all age groups. Children with congenital anomalies were more likely to be prescribed beta-2 agonists (RR=1.71, 95% CI 1.60-1.83) and inhaled corticosteroids (RR=1.74, 95% CI 1.61-1.87). Children with oesophageal atresia, diaphragmatic hernia, genetic syndromes and chromosomal anomalies had over twice the risk of being prescribed anti-asthmatics compared to reference children. Children with congenital anomalies born <32 weeks gestational age were over twice as likely to be prescribed anti-asthmatics than those born at term (RR=2.20, 95% CI 2.10-2.30).

Conclusion: This study documents the additional burden of respiratory symptoms and breathing difficulties for children with congenital anomalies, particularly those born preterm, compared to children without congenital anomalies in the first ten years of life. These findings are beneficial to clinicians and health care providers as they identify children with greater morbidity associated with respiratory symptoms, as indicated by anti-asthmatic prescriptions.

Strengths and limitations of this study

- The use of population-based data from six regions across Europe yielded a large sample size of children with and without congenital anomalies.
- Use of a Common Data Model enabled standardisation of prescription/pharmacy dispensing records across a range of coding classification systems, languages, and healthcare systems in Europe.
- Risks of being prescribed/dispensed medications for respiratory conditions were estimated in children with any major congenital anomaly, in children with 32

For peer review only

For peer review only - http://bmjopen.bmj.com/site/about/guidelines.xhtml

specified isolated congenital anomalies, and for within region comparisons in children with no major congenital anomaly.

- Over 95% of children across registries were linked to prescription databases limiting the potential bias from missed linkages.
- The lack of information on socio-economic status meant this potential confounding factor could not be adjusted for.

Key words: Anti-asthmatics; prescriptions; congenital anomalies; population-based; data linkage

For peer review only

Introduction

Breathing difficulties in childhood are a leading cause of emergency department visits, hospitalisations and missed days at school [1]. The prevalence of chronic breathing conditions such as asthma varies globally in children. The US National Health Interview Survey in 2013 reported prevalence rates of 8.3% in children aged 0-17 years based on parent reports of doctor-diagnosed asthma [2]. The International Study of Asthma and Allergies in Childhood (ISAAC) 1999-2004, which was also based on parental reports, found that prevalence of asthma in European children aged 6-7 years varied from <5% in Albania to over 20% in the United Kingdom (UK), with prevalence rates increasing yearly [3-4]. Similarly, the more recent MeDALL (Mechanisms of the Development of ALLergy) study involving eight birth cohorts across Europe found that parental reports of doctor-diagnosed asthma at age four years ranged from 1.7% in Germany to 13.5% in Bradford, England [5].

The prevalence of asthma also varies according to age and sex in childhood. The ISAAC study consistently found higher asthma rates for 13-14 year olds compared with 6-7 year olds, and this pattern of higher asthma prevalence rates in older children has been replicated across Europe [5-7]. However, as asthma can be challenging to diagnose in infants, wheezing and recurrent wheezing have been used as measures of breathing difficulties in infants. A meta-analysis of European, Latin American and African research reported prevalence rates of 36.1% (95% CI 35.2-37.0%) and 17.4% (95% CI 16.7-18.1%) for wheezing and recurring wheezing respectively for children under two years of age [8]. These prevalence rates were higher than rates of current wheeze in the ISAAC study for 6-7 year olds (11.5%) and 13-14 year olds (14.1%) [9]. Boys have an increased likelihood of parent-reported asthma and wheeze [10-12] and receive more anti-asthmatic prescriptions than girls across Denmark, Sweden and Germany [13-15].

Most of the literature on childhood asthma and respiratory conditions does not differentiate between children with and without congenital anomalies, nor does it explore the additional burden of having chronic and frequent breathing difficulties in children with congenital anomalies. A cohort study based on health records of 170,960 Canadian children up to age six, 1980-1990, reported that children with congenital anomalies of the circulatory system and anomalies of the respiratory system had an increased risk of a recorded asthma diagnosis (HR=1.16, 95% CI 1.03-1.30; and HR=1.43, 95% CI 1.27-1.61) compared to control children [16]. Children under two with diaphragmatic hernia had high hospital readmission rates for wheeze, particularly if born preterm [17]. Most research exploring asthma and breathing difficulties in those with congenital anomalies is based on case series involving small sample sizes. For example, a cohort of adult Dutch patients with unrepaired atrial septal defect reported that four out of 31 (13%) patients had an asthma diagnosis and were taking anti-asthmatic medication [18]. Similarly, a study of 14 Dutch children aged 7-12 years with oesophageal atresia identified that four of the 14 children (29%) had received a diagnosis of asthma [19].

The aim of this study is to compare the risk of being prescribed or dispensed medications for respiratory conditions in children with and without congenital anomalies in six European regions.

For peer review only - http://bmjopen.bmj.com/site/about/guidelines.xhtml

Materials and Methods

This is a EUROlinkCAT population-based data linkage cohort study involving children from 0-9 years of age with and without congenital anomalies in six European regions: Denmark: Funen; Finland; Italy: Emilia Romagna; Italy: Tuscany; Spain: Valencian Region; UK: Wales.

The inclusion criteria was all liveborn children in the registry areas born between 2000 and 2014, or the first birth year included in the study by each registry (Table 1). Five registries included reference children from the whole population covering the registry area, while Tuscany provided a 10% sample of reference children matched on year of birth and sex. Data on children with congenital anomalies were extracted from the European congenital anomaly (EUROCAT) registries and data on reference children were obtained from the birth registers in each region [20-22].

Children were linked to regional or national prescription databases and to hospital outpatient pharmacy databases, if available. Children who could not be linked to these databases were classified as "not linked" and were not included in the study, see Supplementary Table 1. Wales had information on prescriptions issued by the general practitioner (GP) for approximately 70% of Welsh GP practices that were part of the Secure Anonymised Information Linkage (SAIL) databank during the study period, while the other registries had information on prescriptions dispensed by a pharmacy in their region. None of the registries were able to link to hospital inpatient prescribing data or had access to over-the-counter medication.

Anti-asthmatic medication is defined using the World Health Organisation's Anatomical Therapeutic Chemical (ATC) classification system, i.e. ATC codes beginning with R03 (drugs for obstructive airway diseases): beta-2 agonists (R03AC); inhaled corticosteroids (R03BA); anticholinergics (R03BB); antiallergic agents (R03BC); and leukotriene receptor antagonists (R03DC). Data on beta-2 agonists and inhaled corticosteroids were explored individually. Due to small numbers, data on the remaining anti-asthmatic types were included in the 'any anti-asthmatic (R03)' category. Children were classed as exposed to anti-asthmatics if they had been prescribed or dispensed >1 anti-asthmatic prescription in a year in order to minimise children presenting with one-time occurrences of wheeze or infection. Children were classified as unexposed if they were prescribed or dispensed less than two anti-asthmatic prescriptions in a year. Data on anti-asthmatic medications were included from the year 2000 or the first birth year included in the study by each registry up to the end of 2015, resulting in at least one year of follow-up information for each child.

Information on prescriptions were standardised according to a Common Data Model developed as part of the EUROlinkCAT study protocol [21-22]. The EUROCAT data on children with congenital anomalies were already standardised according to EUROCAT guidelines [23]. A common analysis script was sent to all registries to produce aggregate data and analytic results. The aggregate data and analytic results were uploaded to a secure web portal for download by the research team; all individual case data were kept locally.

For peer review only - http://bmjopen.bmj.com/site/about/guidelines.xhtml

Registry	Total number of children included in study		Total receiving >1 anti-asthmatic during whole follow-up		Total receiving >1 inhaled corticosteroid during whole follow-up		Total receiving >1 beta-2 agonist during whole follow-up	
	Children with congenital anomalies	Reference children	Children with congenital anomalies (%)	Reference children (%)	Children with congenital anomalies (%)	Reference children (%)	Children with congenital anomalies (%)	Reference children (%)
Denmark: Funen (2000-2014)	1,789	72,290	614 (34.3%)	20,728 (28.7%)	288 (16.1%)	8,114 (11.2%)	284 (15.9%)	7,684 (10.6%)
Finland (2000-2014)	32,926	755,923	5,617 (17.1%)	99,780 (13.2%)	3,036 (9.2%)	48,866 (6.5%)	3,375 (10.3%)	53,395 (7.1%)
Italy: Emilia Romagna (2008-2014)	5,499	250,829	2,741 (49.8%)	117,675 (46.9%)	1,769 (32.2%)	71,088 (28.3%)	1,087 (19.8%)	41,215 (16.4%)
Italy: Tuscany (2008-2014)	3,048	16,844	1,392 (45.7%)	6,542 (38.8%)	962 (31.6%)	3,947 (23.4%)	560 (18.4%)	1,861 (11.0%)
Spain: Valencian Region (2010-2014)	4,281	223,760	1,816 (42.4%)	77,028 (34.4%)	352 (8.2%)	8,692 (3.9%)	740 (17.3%)	25,518 (11.4%)
UK: Wales (2000-2014)	13,119	403,266	2,540 (19.4%)	60,230 (14.9%)	1,246 (9.5%)	26,732 (6.6%)	2,018 (15.4%)	46,934 (11.6%)
Total	60,662	1,722,912	14,720 (24.3%)	381,983 (22.2%)	7,653 (12.6%)	167,439 (9.7%)	8,064 (13.3%)	176,607 (10.3%)

Table 1. Number and percentages of children with congenital anomalies and reference children prescribed >1 anti-asthmatic medication, by registry

For peer review only - http://bmjopen.bmj.com/site/about/guidelines.xhtml

We explored anti-asthmatic medications in children with any major congenital anomaly (defined as a congenital malformation, deformation, disruption or dysplasia) [20-21], and in 32 isolated congenital anomaly subgroups with a livebirth prevalence of ≥1.75 per 10,000 births to avoid potential issues with small numbers [21] (Supplementary Table 2). Isolated anomalies are defined as anomalies within a single organ, as defined using the EUROCAT algorithm [23].

Each participating registry obtained local approvals for their data to be used in the EUROlinkCAT project. Ethical approval for this study was obtained from Ulster University's Nursing and Health Research Ethics Filter Committee (Reference Number: FCNUR-21-060).

Patient and public involvement

No patient involved.

Statistical analysis

Person-year estimates were calculated, which considers the number of children in the study and the length of follow-up time in the study i.e. each age group includes the number of children alive at the start of that age group. The average years of follow-up for each registry are shown in Supplementary Table 1. Age groups were collapsed into six categories to avoid disclosive counts: <1 year; 1 year; 2-3 years; 4-5 years; 6-7 years; 8-9 years. Data were available from Valencian Region for children 0-5 years of age (i.e. up to the children's 6th birthday), from Italian registries for children 0-7 years of age (i.e. up to 8th birthday), and from Funen, Finland and Wales for children 0-9 years of age (i.e. up to 10th birthday). Analyses of specific congenital anomalies were conducted using data for children aged 0-7 years to include data from the Italian registries.

DerSimonian-Laird (DL) random-effects meta-analyses were conducted to identify both the relative risk (RR) of receiving anti-asthmatic prescriptions relative to the reference group, and the heterogeneity of prevalence rates across registries and by age group [24]. Random-effects meta-analyses were used to estimate RR ratios for the 32 congenital anomaly subgroups, all registries combined, and by class of anti-asthmatic i.e. any anti-asthmatic, inhaled corticosteroids and beta-2 agonists. Heterogeneity was estimated by the statistic I^2. Stata Version 17 was used for data analysis [25].

We explored the effect of receiving >1 anti-asthmatic prescription by birth cohort, sex of child, and gestational age (GA) separately for children with congenital anomalies and reference children e.g. children with congenital anomalies born in 2005-2009 were compared to children with congenital anomalies born 2000-2004; and similarly, reference children born in 2005-2009 were compared to reference children born 2000-2004. Birth cohort was examined for 2000-2004 (baseline group) and 2005-2009, using data from Funen, Finland and Wales as these were the only regions that had data on children born 2000-2004. For the remaining analyses, risk factors were examined for the 0-5 age group for all regions. Girls were used as the baseline group for sex of child analyses. Birth at 37+ weeks GA was used as the baseline group in comparison to birth at <32 weeks GA and birth at 32-36 weeks GA.

For peer review only - http://bmjopen.bmj.com/site/about/guidelines.xhtml

Classifying children as exposed to anti-asthmatics if they had been prescribed or dispensed >1 anti-asthmatic prescription in a year was relaxed in a sensitivity analysis to include children with only 1 prescription/dispensation in a year.

For peer review only

For peer review only - http://bmjopen.bmj.com/site/about/guidelines.xhtml

Results

A total of 63,911 children with congenital anomalies and 1,811,431 reference children up to and including nine years of age were included from six national/regional databases in five countries. Of these, 60,662 (94.9%) children with congenital anomalies and 1,722,912 reference children (95.1%) were included in the study. The percentage of children with and without congenital anomalies prescribed anti-asthmatic medication in each region are shown in Table 1. Overall, 5% of children (ranging from 0% in Tuscany and Valencian Region to 14.6% in Wales) were not included in the study as they could not be linked.

Prescriptions for any anti-asthmatic medicines

There were considerable geographical variations in age-specific prevalence rates of any anti-asthmatic prescription (Figure 1). However, the pattern of prescriptions according to age was similar for children with and without congenital anomalies in all registries. In four registries, the prevalence of >1 prescription for any anti-asthmatic in children with congenital anomalies and reference children peaked at the younger ages (<1 and 1 year), then decreased sharply with age. In Finland and Wales, the prevalence increased until age 2-3 years for all children and then stabilised. Geographical differences in prevalence of >1 prescription for any anti-asthmatic levelled off by age 6-7 years, with rates in children with congenital anomalies ranging between 6-9% in all registries, and rates for reference children ranging between 4-6% in all registries. Prevalence rates for >1 prescription for any anti-asthmatics were consistently slightly higher in children with congenital anomalies than reference children across registries and age groups (Figure 1).

Children with congenital anomalies had a 41% higher risk of >1 prescription for any anti-asthmatic compared to reference children across all age groups and across all registries (RR=1.41, 95% CI 1.35-1.48) (Figure 2). Children aged <1 year with congenital anomalies in Finland had an almost two-fold increase in risk compared to reference children (RR=1.97; 95% CI 1.86-2.08). The wider CIs in older age groups in some registries indicate smaller sample sizes as there were fewer children with long follow-ups. Statistically significant heterogeneity was identified between registries at all ages ($I^2 = 94.3\%$, $p < .001$) due to the large number of reference children, but the differences were small in magnitude.

Prescriptions for inhaled corticosteroids

Children with congenital anomalies had a higher risk of receiving >1 prescription for inhaled corticosteroids than reference children. However, there was heterogeneity between age groups ($p = 0.004$). The risk was highest for children with congenital anomalies aged <1 year (RR=2.12, 95% CI 1.50-2.99) and steadily decreased to 1.35 (95% CI 1.22-1.49) in children aged 8-9 years (Figure 3). The relative risks of receiving >1 prescription for inhaled corticosteroids appeared consistently higher in Valencian Region in children <1 year of age (RR=3.24, 95% CI 2.74-3.84), children at 1 year of age (RR=2.67, 95% CI 2.30-3.10), and children at 2-5 years (RR=2.16, 95% CI 1.91-2.45) compared to children in other registries. Again, there was significant statistical heterogeneity across registries for all ages ($I^2 = 95.6\%$, $p < .001$) due to the large sample sizes.

For peer review only

For peer review only - http://bmjopen.bmj.com/site/about/guidelines.xhtml

Prescriptions for beta-2 agonists

	Children with congenital anomalies (RR; 95% CI)	Reference children (RR; 95% CI)
**Birth Cohort**		

The RR of receiving >1 prescription for beta-2 agonists was 71% higher in children with congenital anomalies compared to reference children (RR=1.71, 95% CI 1.60-1.83) (Figure 3). In contrast to inhaled corticosteroids, the risk of receiving >1 prescription for beta-2-agonists remained consistently high across all age groups (heterogeneity between age groups p = 0.358). Significant heterogeneity was identified across registries for all ages (I^2 = 94.4%, p < .001).

Risk factors for receiving anti-asthmatic prescriptions (children up to age six years)

Children aged 0-5 years were more likely to receive >1 anti-asthmatic per year in 2005-2009 compared to 2000-2004, with this risk being higher in reference children than children with congenital anomalies (Table 2). Children with congenital anomalies born at <32 weeks GA had over twice the risk of being prescribed/dispensed anti-asthmatics (RR= 2.20, 95% CI 2.10-2.30) compared to children born at 37+ weeks. The risk was also raised for reference children (RR=1.86, 95% CI 1.82-1.90). Relative risks decreased in children born 32-36 weeks GA but remained higher in children with congenital anomalies than in reference children (RR= 1.45, 95% CI 1.40-1.49; and RR= 1.31 95% CI 1.30-1.32 respectively). Boys were more likely to be prescribed anti-asthmatics than girls, with similar risks observed for children with congenital anomalies and reference children.

Congenital anomaly subgroups (children up to eight years of age)

The RR for having >1 anti-asthmatic prescription for children with specific congenital anomalies aged 0-7 years was compared to reference children. Children with oesophageal atresia had the highest risk of being prescribed/dispensed any anti-asthmatic medication (RR=3.57, 95% CI 3.14-4.06), inhaled corticosteroids (RR=5.46, 95% CI 4.46-6.68), and beta-2 agonists (RR=5.75, 95% CI 4.86-6.81), Supplementary Table 3. Children with genetic syndromes (Di George syndrome and Noonan syndrome), chromosomal anomalies (all chromosomal anomalies and Down syndrome with and without congenital heart defects), and diaphragmatic hernia had more than double the risk of having >1 prescription for any anti-asthmatics compared to reference children. Across all 32 congenital anomaly subgroups examined, children with these anomalies were significantly more likely to be prescribed any anti-asthmatic than reference children, except for children with Tetralogy of Fallot or children with hip dislocation and/or dysplasia whose risks were slightly raised but were not statistically significant.

The relative risk for receiving >1 prescription for beta-2-agonists and for inhaled corticosteroids for the 32 congenital anomaly subgroups followed the same trends as those for all anti-asthmatic medication, but they were less likely to be statistically significant due to smaller numbers being analysed.

For peer review only

For peer review only - http://bmjopen.bmj.com/site/about/guidelines.xhtml

2000-2004	Reference	
2005-2009	1.27 (1.23-1.31)	1.60 (1.59-1.61)
**Gestation Age**		
37+ weeks	Reference	
32-36 weeks	1.45 (1.40-1.49)	1.31 (1.30-1.32)
<32 weeks	2.20 (2.10-2.30)	1.86 (1.83-1.90)
**Sex of Child**		
Female	Reference	
Male	1.38 (1.35-1.41)	1.35 (1.35-1.36)

Table 2. Relative risk (RR) of >1 anti-asthmatic prescriptions in children with congenital anomalies and reference children up to age six years, by risk factor

For the birth cohort analysis, data were analysed only from three registries: Denmark: Funen, Finland, and Wales.

For peer review only

For peer review only - http://bmjopen.bmj.com/site/about/guidelines.xhtml

Sensitivity analysis

The overall RR of children with congenital anomalies receiving at least one prescription for any anti-asthmatic medication was 1.25 (95% CI 1.21-1.28) compared to reference children (Supplementary Table 4). This is compared to the RR = 1.41 (95% CI 1.35-1.48) of receiving >1 prescription. Although the risk estimates were lower for children with at least one prescription for any anti-asthmatic, the pattern of risk across registries and age groups was similar to that found for children with >1 prescription (Supplementary Figure 1).

For peer review only

Discussion

This population-based cohort study of over 1.78 million children in six European regions found that across all regions and age groups, children with congenital anomalies had a consistently higher risk of receiving prescriptions for >1 anti-asthmatic medication compared to reference children. We observed geographical variation in the use of anti-asthmatic medications with registries in Denmark, Italy and Spain having higher prevalence in young children whereas Finland and Wales had lower prevalence. By age 6-7 years, the prevalence in the individual registries started to converge for both children with and without anomalies, although it remained slightly higher for children with congenital anomalies. Children with oesophageal atresia, diaphragmatic hernia, chromosomal anomalies and specific genetic syndromes had the highest risk of being prescribed anti-asthmatic prescriptions. Male children, children born in a later cohort, and children born preterm had an increased risk of anti-asthmatic prescriptions, with children with congenital anomalies born very preterm (<32 weeks) having over double the risk of anti-asthmatic prescriptions compared to children with congenital anomalies born at term.

With the exception of Finland and Wales, our findings that anti-asthmatic prescriptions tended to be higher in younger age groups is consistent with literature indicating higher rates of wheezing and current wheeze in infants below two years of age than older children [8-9]. As beta-2 agonists and inhaled corticosteroids are recommended as treatment for infant wheeze [26], prescriptions for anti-asthmatics in young children may be indicative of wheeze and acute respiratory infections which can be common in young children [27]. However, other studies reported higher prevalence of breathing difficulties for older children in the form of parent-reported asthma [3]. It is possible that parental reports may overestimate asthma prevalence, while our study was based on administrative anti-asthmatic prescription records. We identified anti-asthmatic prevalence rates that seemed to vary from other regional studies. For example, a study based on a sample of Welsh children (n=1,529), born 2000-2002, with a GP diagnosis of asthma and/or prescriptions for anti-asthmatics in their health records in the last 12 months reported much higher prevalence rates ranging from 13.4% for 3 year olds to 10.4% in 7 year olds [28]. These differences may relate to methodology as our study criteria was >1 anti-asthmatic prescription in a year rather than at least one. We also identified regional differences within countries as within Italy, children in Emilia Romagna had a higher prevalence of >1 prescription for asthma medications than children in Tuscany. A study investigating the use of anti-asthmatics in women before, during and after pregnancy also reported this regional difference between the two Italian registries [29].

Children with congenital anomalies consistently had higher rates of anti-asthmatic medication than reference children which may be due to several factors. Congenital anomalies such as diaphragmatic hernia and Down syndrome are associated with pulmonary hypoplasia [30-33], requiring anti-asthmatics to manage wheezing episodes. Children with diaphragmatic hernia commonly require mechanical ventilation for days or weeks after birth which can lead to bronchopulmonary dysplasia [31; 34]. Beta-2 agonists are prescribed to children with bronchopulmonary dysplasia to reduce pulmonary resistance

For peer review only

For peer review only - http://bmjopen.bmj.com/site/about/guidelines.xhtml

[35]. Studies have shown that patients with diaphragmatic hernia were over six times more likely to have been diagnosed with asthma than the general population [34]. Congenital anomalies may be comorbid with respiratory conditions, such as the associations between oesophageal atresia, Down syndrome and Di George syndrome with respiratory tract disease [36-38]. We did not find an increased risk for prescription of beta-2 agonists for children with Tetralogy of Fallot. Beta-2-agonists should not be used in children with infundibular pulmonary stenosis as beta-blockers may be prescribed to prevent hypoxic spells in these children while awaiting surgery [39].

The finding that boys were more likely to be prescribed/dispensed anti-asthmatics is consistent with previous literature [10-15]. Both children with and without congenital anomalies had more anti-asthmatic prescriptions in 2005-2009 compared to 2000-2004. While the magnitude of risk was higher in reference children than for children with congenital anomalies, the comparisons were not between children with congenital anomalies and reference children, but within the two groups of children. Indeed, children with congenital anomalies had a slightly higher proportion of >1 anti-asthmatic prescription (24%) compared to reference children (22%). While self-reported anti-asthmatic use has increased in cohort studies of young adults between 1990-2007 [40], our findings support an increasing trend of anti-asthmatic prescriptions over time in children using administrative prescription data. Children born preterm at <32 weeks GA were twice as likely to be prescribed/dispensed anti-asthmatics than children born at term, although the risk was reduced for children born 32-36 weeks. The higher risk in children born <32 weeks GA may be due to respiratory complications such as respiratory distress syndrome or bronchopulmonary dysplasia arising from mechanical ventilation in preterm children [41-42].

A major strength of this study is that we have standardised data on congenital anomalies and a Common Data Model which enabled us to map and standardise the prescription/pharmacy dispensing records, as well as standardising demographic information on children. This is important as there are diverse coding classification systems, languages, and healthcare systems in Europe. Furthermore, this is the first population-based multi-centre study exploring anti-asthmatic prescriptions in children with and without congenital anomalies which included data from 60,662 children with congenital anomalies across Europe, overcoming limitations of previous research based on small sizes. The use of reference children for comparison in each geographical region is also a major strength, as some differences in prescription rates across Europe may be explained by methodological issues (such as frequency of prescriptions issued at the pharmacy) and different level of indication for prescribing asthma medications to small children.

However, there were a number of limitations to this study. Firstly, bias may arise as approximately 5% of children across registries were unable to be linked to the prescription database due to invalid identifications numbers as was the case in Tuscany, or because their records were not sent to the national/vital statistics to be linked. In Wales, the children were registered in a GP practice that was not part of the SAIL databank, therefore they could not be linked. There is evidence suggesting that low socioeconomic status (SES) is

For peer review only - http://bmjopen.bmj.com/site/about/guidelines.xhtml

associated with asthma [43]. Wales compared the SES of children registered in SAIL versus children not registered in SAIL and concluded that there were no differences in SES between linked/unlinked children. Similarly, Tuscany found no differences in gestational age, maternal age, sex and survival between children with congenital anomalies who were linked versus those who were not linked. Secondly, only three participating registries (Funen, Finland and Wales) could provide data for children up to and including nine years of age. Our list of anti-asthmatic medications did not include oral corticosteroids used to treat acute symptoms, but for young children this treatment may mainly be done during hospital stays, and we were unable to differentiate between long-acting and short-acting beta-2-agonists. While we explored medications prescribed and dispensed, we were unable to gauge whether these medications were actually taken.

In summary, this study demonstrates that valid information on the prevalence of anti-asthmatic prescriptions for children can be obtained through data linkage studies in order to monitor regional differences across age groups and over time. In general, children with congenital anomalies had a higher prevalence and risk of anti-asthmatic prescriptions than reference children. To evaluate the additional burden of disease that children with congenital anomalies face, it is essential to compare these children to those without congenital anomalies in the same geographic areas as prescription patterns of anti-asthmatic medications vary across Europe. The findings from this study are also beneficial to clinicians for identifying which congenital anomalies are associated with a higher risk of prescriptions for respiratory symptoms and breathing difficulties.

For peer review only

For peer review only - http://bmjopen.bmj.com/site/about/guidelines.xhtml

Contributorship Statement: ND prepared the manuscript and revised it following feedback and comments from all authors. ND analysed the pooled data from each registry. JG and ML produced the standardisation scripts. JT and JKM produced the central analysis scripts. GA, EB, LBB, CC, AC, MG, AH, SJ, AP, IS, SKU extracted and analysed local registry data. ML, EG and JKM secured funding, conceptualised and designed the study, and provided frequent feedback on manuscript drafts. JKM and ML provided data analysis guidance and syntax for meta-analyses. ML executed data analysis syntax relating to protected data.

Competing Interests: None

Funding Statement: This work was supported by the European Union's Horizon 2020 research and innovation programme under grant agreement No. 733001. The views presented here are those of the authors only, and the European Commission is not responsible for any use that may be made of the information presented here.

Data Sharing Statement: We are legally not allowed to share the third-party administrative data used in this study as it belongs to the data providers in each of the regions i.e. the regional or national statistical organisations. All our documentation is available on the EUROlinkCAT website (http://www.EUROlinkCAT.eu/wp2-buildingresultsrepository). and we encourage any interested parties to apply to the EUROlinkCAT management team to assist them in obtaining approval from the data providers in each region/ country to use the aggregated data for an approved study http://www.EUROlinkCAT.eu/contactinformationanddatarequests

Ethics Approval Statement: Ethical approval for this study was obtained from Ulster University's Nursing and Health Research Ethics Filter Committee (Reference Number: FCNUR-21-060). This study is based on secondary data analysis of data held in routine administrative databases in Europe. The individual records of children linked to electronic prescription databases remain within the regional/national statistical organisations. Only aggregated results were sent to the research team for analysis, hence informed consent was not required. Each participating registry obtained local approvals for their data to be used in the EUROlinkCAT project as described in a paper accepted for publication by the BMJ Open: Claridge H et al "Ethics and legal requirements for data linkage in 14 European countries".

For peer review only

For peer review only - http://bmjopen.bmj.com/site/about/guidelines.xhtml

References

1. World Health Organisation. Asthma [Internet]. World Health Organisation; 2021 [cited 2021 Aug 18]. Available from: https://www.who.int/news-room/fact-sheets/detail/asthma
2. Akinbami LJ, Simon AE, Rossen LM. Changing trends in asthma prevalence among children. Pediatrics. 2016 Jan 1;137(1).
3. Wirl C, Puklova V. Prevalence of asthma and allergies in children. *European Environment and Health Information System Fact Sheet, World Health Organization. 2007*; Fact Sheet No 3.1 (May).
4. Pearce N, Aït-Khaled N, Beasley R, Mallol J, Keil U, Mitchell E, Robertson C. Worldwide trends in the prevalence of asthma symptoms: phase III of the International Study of Asthma and Allergies in Childhood (ISAAC). Thorax. 2007 Sep 1;62(9):758-66.
5. Uphoff EP, Bird PK, Antó JM, Basterrechea M, Von Berg A, Bergström A, Bousquet J, Chatzi L, Fantini MP, Ferrero A, Gehring U. Variations in the prevalence of childhood asthma and wheeze in MeDALL cohorts in Europe. ERJ open research. 2017 Jul 1;3(3).
6. Bloom CI, Saglani S, Feary J, Jarvis D, Quint JK. Changing prevalence of current asthma and inhaled corticosteroid treatment in the UK: population-based cohort 2006–2016. European Respiratory Journal. 2019 Apr 1;53(4).
7. Sestini P, De Sario M, Bugiani M, Bisanti L, Giannella G, Kaisermann D, Frasca G, Lombardi E, Petronio MG, Dell'Orco V, Indinnimeo L. Frequency of asthma and allergies in Italian children and adolescents: results from SIDRIA-2. Epidemiologia e prevenzione. 2005 Mar 1;29(2 Suppl):24-31
8. Alvarez-Alvarez I, Niu H, Guillen-Grima F, Aguinaga-Ontoso I. Meta-analysis of prevalence of wheezing and recurrent wheezing in infants. Allergologia et immunopathologia. 2018 May 1;46(3):210-7.
9. Lai CK, Beasley R, Crane J, Foliaki S, Shah J, Weiland S, ISAAC Phase Three Study Group. Global variation in the prevalence and severity of asthma symptoms: phase three of the International Study of Asthma and Allergies in Childhood (ISAAC). Thorax. 2009 Jun 1;64(6):476-83.
10. Duggan EM, Sturley J, Fitzgerald AP, Perry IJ, Hourihane JO. The 2002–2007 trends of prevalence of asthma, allergic rhinitis and eczema in Irish schoolchildren. Pediatric allergy and immunology. 2012 Aug;23(5):464-71.
11. Ciccone G, Camarlengo A, Bugiani M, Dalmasso P, Faggiano F, Volante T, Magnani C, Natale P, Piccioni P, Bisanti L, Gianelle V. Asthma and respiratory symptoms in 6-7 yr old Italian children: gender, latitude, urbanization and socioeconomic factors. Eur Respir J. 1997 Aug 1;10:1780-6.
12. Linneberg A, Simonsen JB, Petersen J, Stensballe LG, Benn CS. Differential effects of risk factors on infant wheeze and atopic dermatitis emphasize a different etiology. Journal of Allergy and Clinical Immunology. 2006 Jan 1;117(1):184-9.
13. Henriksen L, Simonsen J, Haerskjold A, Linder M, Kieler H, Thomsen SF, Stensballe LG. Incidence rates of atopic dermatitis, asthma, and allergic rhinoconjunctivitis in

For peer review only - http://bmjopen.bmj.com/site/about/guidelines.xhtml

Danish and Swedish children. Journal of Allergy and Clinical Immunology. 2015 Aug 1;136(2):360-6.

14. Hoffmann F, Glaeske G. Prescriptions as a proxy for asthma in children: a good choice?. European journal of clinical pharmacology. 2010 Mar;66(3):307-13.
15. Stock S, Redaelli M, Luengen M, Wendland G, Civello D, Lauterbach KW. Asthma: prevalence and cost of illness. European Respiratory Journal. 2005 Jan 1;25(1):47-53.
16. Dik N, Tate RB, Manfreda J, Anthonisen NR. Risk of physician-diagnosed asthma in the first 6 years of life. Chest. 2004 Oct 1;126(4):1147-53.
17. Benoist G, Mokhtari M, Deschildre A, Khen-Dunlop N, Storme L, Benachi A, Delacourt C. Risk of readmission for wheezing during infancy in children with congenital diaphragmatic hernia. PloS one. 2016 May 12;11(5):e0155556
18. Nassif M, van Steenwijk RP, Hogenhout JM, Lu H, de Bruin-Bon RH, Hirsch A, Sterk PJ, Bouma BJ, Straver B, Tijssen JG, Mulder BJ. Atrial septal defect in adults is associated with airway hyperresponsiveness. Congenital heart disease. 2018 Nov;13(6):959-66.
19. Agrawal L, Beardsmore CS, MacFadyen UM. Respiratory function in childhood following repair of oesophageal atresia and tracheoesophageal fistula. Archives of disease in childhood. 1999 Nov 1;81(5):404-8.
20. Boyd PA, Haeusler M, Barisic I, Loane M, Garne E, Dolk H. Paper 1: The EUROCAT network—organization and processes. *Birth Defects Research Part A, Clinical and Molecular Teratology.* 2011;91(S1):S2-S15.
21. Morris JK, Garne E, Loane M, Barisic I, Densem J, Latos-Bieleńska A, et al. EUROlinkCAT protocol for a European population-based data linkage study investigating the survival, morbidity and education of children with congenital anomalies. BMJ Open. 2021;11(6):e047859.
22. Loane M, Given JE, Tan J, Astolfi G, Barisic I, Carbonell CC, et al. (2021) Creating a population-based cohort of children born with and without congenital anomalies using birth data linked to hospital discharge databases in 11 European regions: assessment of linkage success and data quality. PLoS ONE (submitted)
23. EUROCAT (2020). EUROCAT Guide 1.4: instruction for the registration of congenital anomalies. EUROCAT Central Registry, Ulster University
24. DerSimonian R, Laird N. Meta-analysis in clinical trials. Control Clin Trials. 1986; 7(3):177–88. https://doi.org/10.1016/0197-2456(86)90046-2.
25. StataCorp. 2021. *Stata Statistical Software: Release 17*. College Station, TX: StataCorp LLC.
26. Brand PL, Baraldi E, Bisgaard H, Boner AL, Castro-Rodriguez JA, Custovic A, de Blic J, De Jongste JC, Eber E, Everard ML, Frey U. Definition, assessment and treatment of wheezing disorders in preschool children: an evidence-based approach. European Respiratory Journal. 2008 Oct 1;32(4):1096-110.
27. Nair H, Nokes DJ, Gessner BD, Dherani M, Madhi SA, Singleton RJ, O'Brien KL, Roca A, Wright PF, Bruce N, Chandran A. Global burden of acute lower respiratory infections due to respiratory syncytial virus in young children: a systematic review and meta-analysis. The Lancet. 2010 May 1;375(9725):1545-55.

For peer review only

For peer review only - http://bmjopen.bmj.com/site/about/guidelines.xhtml

28. Griffiths LJ, Lyons RA, Bandyopadhyay A, et al. Childhood asthma prevalence: cross-sectional record linkage study comparing parent-reported wheeze with general practitioner-recorded asthma diagnoses from primary care electronic health records in Wales. BMJ Open Resp Res 2018;5:e000260. doi:10.1136/ bmjresp-2017-000260
29. Charlton RA, Pierini A, Klungsøyr K, Neville AJ, Jordan S, Thayer D, Bos HJ, Puccini A, Hansen AV, Gini R, Engeland A. Asthma medication prescribing before, during and after pregnancy: a study in seven European regions. BMJ open. 2016 Jan 1;6(1):e009237.
30. Cooney TP, Thurlbeck WM. Pulmonary hypoplasia in Down's syndrome. New England Journal of Medicine. 1982 Nov 4;307(19):1170-3.
31. Ijsselstijn H, Tibboel D, Hop WJ, Molenaar JC, de Jongste JC. Long-term pulmonary sequelae in children with congenital diaphragmatic hernia. Am J Respir Crit Care Med 1997;155:174–180.
32. Logan JW, Rice HE, Goldberg RN, Cotten CM. Congenital diaphragmatic hernia: a systematic review and summary of best-evidence practice strategies. Journal of Perinatology. 2007 Sep;27(9):535-49.
33. Wang J, Liu H, Gao L, Liu X. Impaired FGF10 signaling and epithelial development in experimental lung hypoplasia with esophageal atresia. Frontiers in pediatrics. 2018 Apr 20;6:109.
34. Spoel M, van der Cammen-van Zijp MH, Hop WC, Tibboel D, de Jongste JC, IJsselstijn H. Lung function in young adults with congenital diaphragmatic hernia; a longitudinal evaluation. Pediatric pulmonology. 2013 Feb;48(2):130-7.
35. Pfenninger J, Aebi C. Respiratory response to salbutamol (albuterol) in ventilator-dependent infants with chronic lung disease: pressurized aerosol delivery versus intravenous injection. Intensive care medicine. 1993 May;19(5):251-5.
36. Usui N, Kamata S, Ishikawa S, Sawai T, Okuyama H, Imura K, Okada A. Anomalies of the tracheobronchial tree in patients with esophageal atresia. Journal of pediatric surgery. 1996 Feb 1;31(2):258-62.
37. Verheij E, Speleman L, van der Molen AB, Thomeer HG. Congenital respiratory tract disorders in 22q11. 2 deletion syndrome. International journal of pediatric otorhinolaryngology. 2018 Jan 1;104:1-4.
38. Colvin KL, Yeager ME. What people with Down Syndrome can teach us about cardiopulmonary disease. European Respiratory Review. 2017 Mar 31;26(143).
39. Fanous E, Mogyorósy G. Does the prophylactic and therapeutic use of beta-blockers in preoperative patients with tetralogy of Fallot significantly prevent and treat the occurrence of cyanotic spells? Interactive cardiovascular and thoracic surgery. 2017 Oct 1;25(4):647-50.
40. Sigurkarlsson S, Clausen M, Gislason T, Gislason D. Prevalence of respiratory symptoms and use of asthma drugs are increasing among young adult Icelanders. Laeknabladid. 2011 Sep 1;97(9):463-7.
41. Fraser J, Walls M, McGuire W. Respiratory complications of preterm birth. Bmj. 2004 Oct 21;329(7472):962-5.
42. Xu YP. Bronchopulmonary dysplasia in preterm infants born at less than 32 weeks gestation. Global pediatric health. 2016 Sep 13;3:2333794X16668773.

For peer review only - http://bmjopen.bmj.com/site/about/guidelines.xhtml

43. Uphoff E, Cabieses B, Pinart M, et al. A systematic review of socioeconomic position in relation to asthma and allergic diseases. Eur Respir J 2015; 46: 364–374

For peer review only

For peer review only - http://bmjopen.bmj.com/site/about/guidelines.xhtml

Legends

Figure 1. Prevalence of >1 prescription for any anti-asthmatic per 100 children in each age group, by registry

Figure 2. Relative risk (RR) of >1 prescription for any anti-asthmatic (AA) in children with congenital anomalies (CAs) compared to reference children, by registry
CI=Confidence intervals,
DL=DerSimonian-Laird

Figure 3. Relative risk (RR) of >1 beta-2 agonist (B2A) prescription and >1 inhaled corticosteroid (ICS) prescription in children with congenital anomalies (CAs) compared to reference children, by registry
CI=Confidence interval
DL=DerSimonian-Laird

For peer review only

For peer review only - http://bmjopen.bmj.com/site/about/guidelines.xhtml

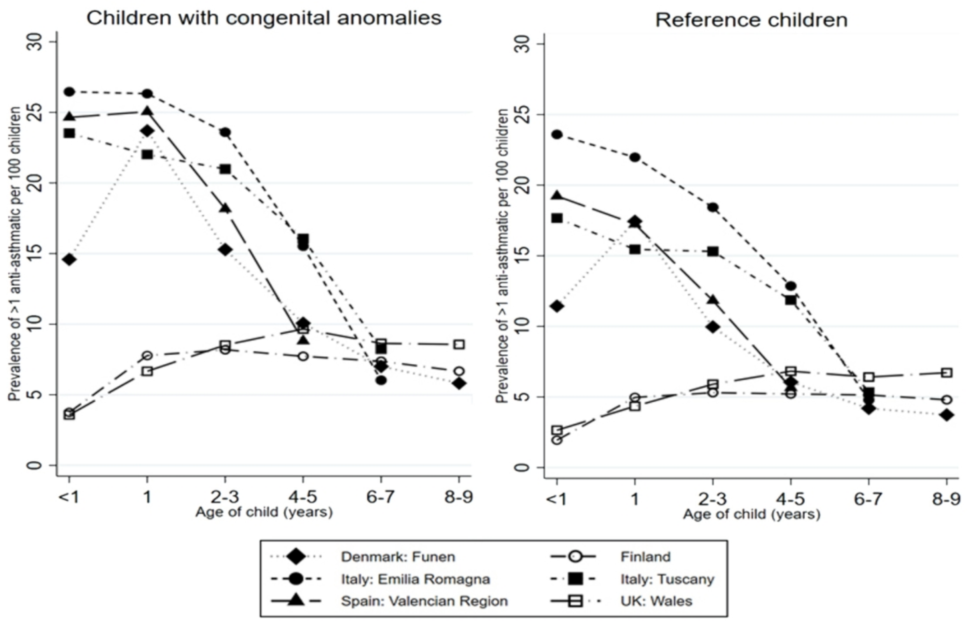

Figure 1. Prevalence of >1 prescription for any anti-asthmatic per 100 children in each age group, by registry

582x366mm (72 x 72 DPI)

Figure 2. Relative risk (RR) of >1 prescription for any anti-asthmatic (AA) in children with congenital anomalies (CAs) compared to reference children, by registry
CI=Confidence intervals,
DL=DerSimonian-Laird

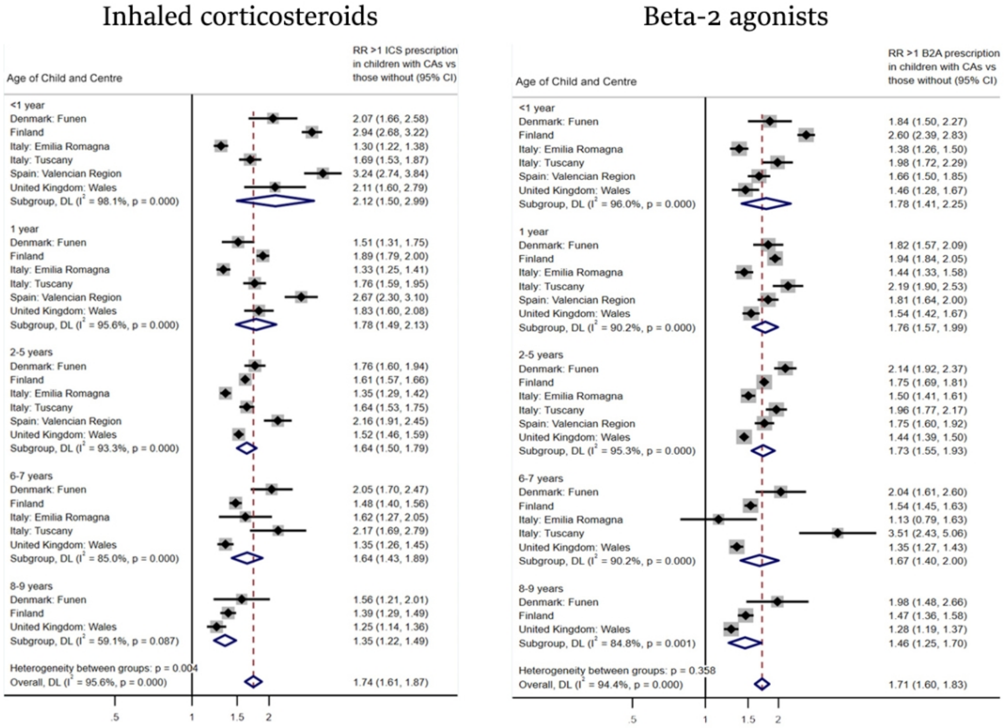

Figure 3. Relative risk (RR) of >1 beta-2 agonist (B2A) prescription and >1 inhaled corticosteroid (ICS) prescription in children with congenital anomalies (CAs) compared to reference children, by registry
CI=Confidence interval
DL=DerSimonian-Laird

426x318mm (72 x 72 DPI)

Registry	Birth years included	Average follow-up years (years)	Total number of children in population		Number of children included in the study		Number of children not linked*	
			Children with congenital anomalies	Reference children	Children with congenital anomalies (%)	Reference children (%)	Children with congenital anomalies (%)	Reference children (%)
**Denmark: Funen**	2000-2014	7.3	1,813	72,382	1,789 (98.7)	72,290 (99.9)	24 (1.3)	92 (0.1)
**Finland**	2000-2014	7.2	33,177	762,989	32,926 (99.2)	755,923 (99.1)	251 (0.8)	7,066 (0.9)
**Italy: Emilia Romagna**	2008-2014	4.6	5,801	263,574	5,499 (94.8)	250,829 (95.2)	302 (5.2)	12,745 (4.8)
**Italy: Tuscany**	2008-2014	4.4	3,445	16,844	3,048 (88.5)	16,844 (100)	397 (11.5)	0
**Spain: Valencian Region**	2010-2014	3.6	4,308	223,760	4,281 (99.4)	223,760 (100)	27 (0.6)	0
**UK: Wales**	2000-2014	6.7	15,367	471,882	13,119 (85.4)	403,266 (85.5)	2,248 (14.6)	68,616 (14.5)
**Total**			63,911	1,811,431	60,662 (94.9)	1,722,912 (95.1)	3,249 (5.1)	88,519 (4.9)

*These children were excluded from the study

Supplementary Table 1. Number and percentage of children included in the study by registry

For peer review only

Type of Anomaly*	Congenital Anomaly	ICD10-BPA Code	ICD9 Code
Isolated*	Spina bifida	Q05	741
	Hydrocephalus	Q03	7423
	Anomalies of the corpus callosum	Q040	74221
	Severe microcephaly	Q02	7421
	Congenital heart defects (CHD)**	Q20-Q26	745, 746, 7470-7474
	Severe CHD***	Q200, Q201, Q203, Q204, Q212, Q213, Q220, Q224, Q225, Q226, Q230, Q232, Q233, Q234, Q251, Q252, Q262	74500, 74510, 7452, 7453, 7456, 7461, 7462, 74600, 7463, 7465, 7466, 7467, 7471, 74720, 74742
	Transposition of great arteries	Q203	74510
	Ventricular septal defect	Q210	7454
	Atrial septal defect	Q211	7455
	Tetralogy of Fallot	Q213	7452
	Coarctation of the aorta	Q251	7471
	Patent ductus arteriosus	Q250	7470
	Cleft lip (with or without cleft palate)	Q36, Q37	7491, 7492
	Cleft palate	Q35	7490
	Oesophageal atresia	Q390-Q391	75030-75031
	Anorectal atresia	Q420-Q423	75121-75124
	Diaphragmatic hernia	Q790	75661
	Gastroschisis	Q793	75671
	Multicystic renal dysplasia	Q6140, Q6141	75316
	Congenital hydronephrosis	Q620	75320
	Hypospadias	Q54	75260
	Limb reduction defects	Q71-Q73	7552-7554
	Club foot	Q660	75450
	Hip dislocation and/or dysplasia	Q650-Q652, Q6580, Q6581	75430
	Craniosynostosis	Q750	75600
Chromosomal	Down syndrome	Q90	7580
	Down syndrome without CHD	Q90 excluding codes Q20-Q26	7580 excluding codes 745, 746, 7470-7474

For peer review only

	Down syndrome with CHD	Q90 including codes Q20-Q26	7580 including codes 745, 746, 7470-7474
	Turner syndrome	Q96	75860, 75861, 75862, 75869
Genetic syndromes	Di George syndrome	D821	27910
	Noonan syndrome	Q8714	759896

Supplementary Table 2. Congenital anomalies and relevant International Classification of Diseases (ICD)-British Paediatric Association (BPA) codes

*Based on the EUROCAT algorithm [13]

**CHD includes all the anomalies listed under severe CHD, see below, as well as atrial septal defect, ventricular septal defect, pulmonary valve stenosis and patent ductus arteriosus in term infants

***Severe CHD included the following CHD subgroups: common arterial truncus, double outlet right ventricle, transposition of great vessels, single ventricle, atrioventricular septal defect, tetralogy of Fallot, pulmonary valve atresia, tricuspid atresia and stenosis, Ebstein anomaly, hypoplastic right heart, aortic valve atresia/stenosis, mitral valve anomalies, hypoplastic left heart, coarctation of aorta, aortic atresia/interrupted aortic arch, total anomalous pulmonary venous return.

For peer review only

Isolated Anomaly	Any Anti-Asthmatic (RR, 95% CI)	Beta-2 Agonists (RR, 95% CI)	Inhaled Corticosteroids (RR, 95% CI)
**Spina bifida** n = 154	1.68 (1.35-2.09)	2.53 (1.89-3.38)	2.52 (1.97-3.23)
**Hydrocephalus** n = 299	1.59 (1.41-1.80)	2.24 (1.86-2.69)	2.04 (1.71-2.45)
**Severe microcephaly** n = 278	1.53 (1.30-1.79)	2.15 (1.63-2.84)	1.82 (1.44-2.31)
**Anomalies of corpus callosum** n = 471	1.94 (1.70-2.22)	3.44 (2.90-4.08)	2.90 (2.53-3.33)
**Congenital heart defects (CHD)** n = 19,643	1.31 (1.27-1.36)	1.47 (1.40-1.54)	1.48 (1.39-1.57)
**Severe CHD** n = 3,368	1.42 (1.33-1.52)	1.60 (1.47-1.74)	1.83 (1.65-2.02)
**Transposition of great arteries** n = 488	1.46 (1.28-1.66)	1.91 (1.62-2.24)	2.10 (1.81-2.43)
**Ventricular septal defect** n = 11,826	1.21 (1.18-1.24)	1.35 (1.30-1.41)	1.26 (1.20-1.31)
**Atrial septal defect** n = 2,679	1.43 (1.33-1.54)	1.51 (1.37-1.67)	1.53 (1.37-1.70)
**Tetralogy of Fallot** n = 426	1.10 (0.97-1.25)	1.19 (0.94-1.49)	1.52 (1.26-1.84)
**Coarctation of aorta** n = 1,021	1.72 (1.56-1.90)	2.09 (1.85-2.36)	2.12 (1.88-2.38)
**Patent ductus arteriosus** n = 385	1.57 (1.40-1.76)	2.24 (1.81-2.77)	2.23 (1.84-2.71)
**Cleft lip (with or without cleft palate)** n = 1,299	1.27 (1.19-1.36)	1.62 (1.47-1.80)	1.31 (1.18-1.45)
**Cleft palate** n = 1,164	1.49 (1.35-1.65)	1.79 (1.49-2.15)	1.71 (1.49-1.96)
**Oesophageal atresia** n = 239	3.57 (3.14-4.06)	5.75 (4.86-6.81)	5.46 (4.46-6.68)
**Anorectal atresia** n = 236	1.40 (1.20-1.64)	1.92 (1.48-2.49)	1.82 (1.45-2.28)
**Diaphragmatic hernia** n = 211	2.28 (1.72-3.03)	3.70 (2.85-4.81)	3.34 (2.48-4.50)
**Gastroschisis** n = 376	1.45 (1.27-1.67)	1.85 (1.52-2.25)	1.44 (1.15-1.80)

**Multicystic renal dysplasia** n = 621	1.31 (1.18-1.46)	1.83 (1.58-2.11)	1.56 (1.33-1.83)
**Congenital hydronephrosis** n = 3,026	1.21 (1.15-1.28)	1.33 (1.20-1.46)	1.27 (1.18-1.38)
**Hypospadias** n = 2,526	1.30 (1.22-1.39)	1.52 (1.37-1.69)	1.44 (1.32-1.57)
**Limb reduction defects** n = 445	1.16 (1.03-1.32)	1.40 (1.14-1.72)	1.32 (1.06-1.64)
**Club foot** n = 1,997	1.31 (1.24-1.38)	1.46 (1.34-1.59)	1.42 (1.31-1.54)
**Hip dislocation and/or dysplasia** n = 1,026	1.05 (0.96-1.15)	1.03 (0.91-1.17)	1.22 (1.07-1.39)
**Craniosynostosis** n = 765	1.37 (1.26-1.49)	1.70 (1.50-1.93)	1.46 (1.27-1.67)
**All chromosomal** n = 3,608	2.19 (2.00-2.40)	2.86 (2.47-3.31)	2.94 (2.58-3.34)
**Down syndrome** n = 1,993	2.19 (2.00-2.40)	2.77 (2.40-3.20)	2.88 (2.54-3.28)
**Down syndrome (including CHD)** n = 1,131	2.33 (2.09-2.61)	2.93 (2.47-3.48)	3.12 (2.70-3.61)
**Down syndrome (excluding CHD)** n = 862	2.11 (1.93-2.31)	2.76 (2.39-3.19)	2.73 (2.31-3.23)
**Turner syndrome** n = 143	1.48 (1.21-1.82)	2.50 (1.91-3.28)	2.15 (1.64-2.82)
**Di George syndrome** n = 172	3.47 (3.04-3.95)	5.11 (4.35-6.01)	4.88 (4.01-5.94)
**Noonan syndrome** n = 120	2.33 (1.99-2.72)	2.76 (2.09-3.64)	3.68 (2.82-4.82)

Supplementary Table 3. Combined relative risks (RR) across registries for >1 anti-asthmatic prescriptions up to age eight years, by anomaly

	RR of receiving ≥1 prescription for any anti-asthmatic compared to reference children	RR of receiving >1 prescription for any anti-asthmatic compared to reference children
**<1 year**	1.17 (1.10-1.25)	1.39 (1.17-1.65)
**1 year**	1.22 (1.17-1.27)	1.41 (1.29-1.55)
**2-5 years**	1.27 (1.19-1.35)	1.44 (1.35-1.53)
**6-7 years**	1.33 (1.26-1.41)	1.43 (1.32-1.54)
**8-9 years**	1.30 (1.19-1.41)	1.37 (1.25-1.51)
**All years combined**	1.25 (1.21-1.28)	1.41 (1.35-1.48)

Supplementary Table 3. Relative risk (RR) of receiving ≥1 prescription versus receiving >1 prescription for any anti-asthmatic in children with congenital anomalies compared to reference children

For peer review only

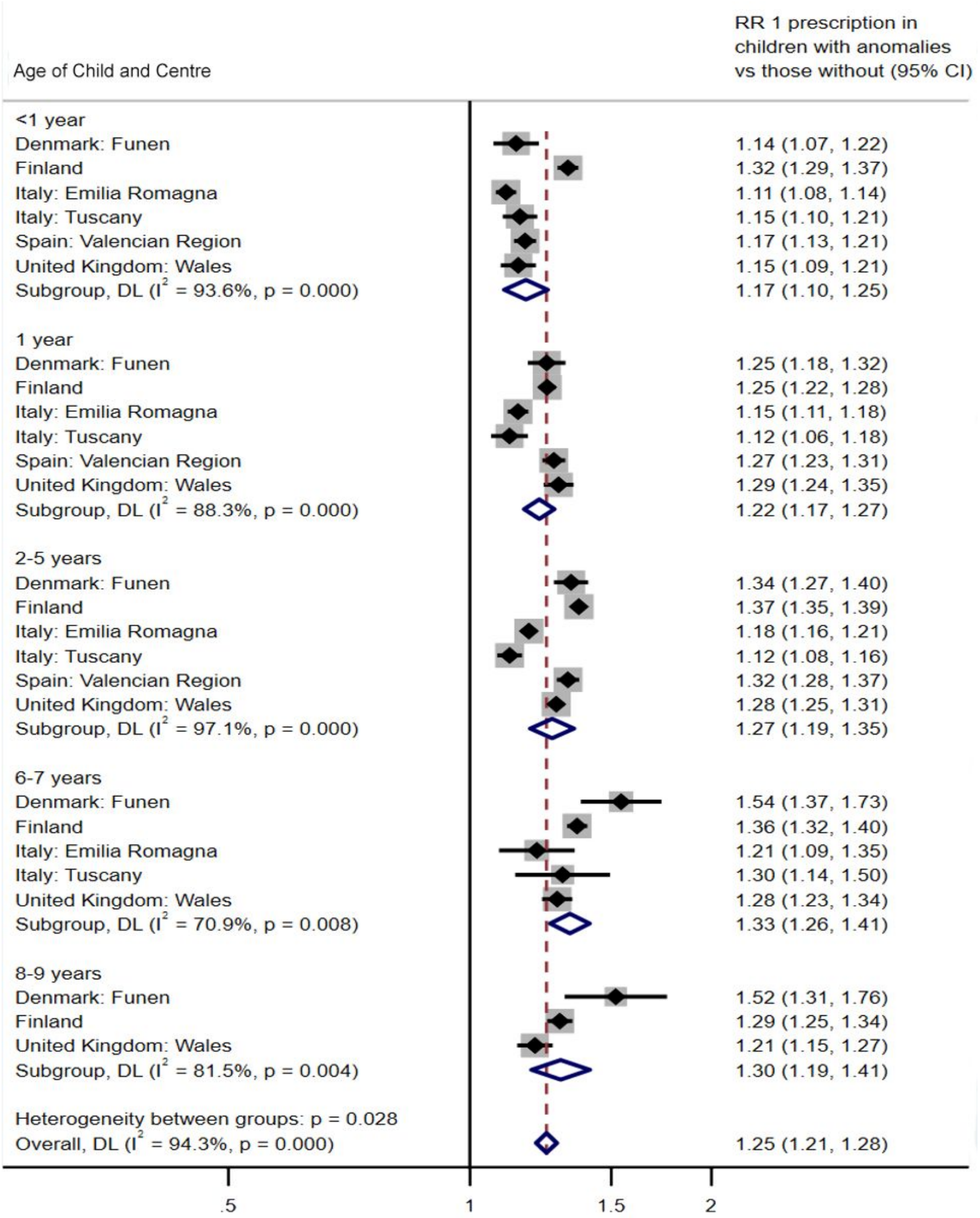

Supplementary Figure 1. Relative risk (RR) of at least 1 prescription for any anti-asthmatic in children with congenital anomalies (CAs) compared to reference children, by registry
CI=Confidence interval,
DL=DerSimonian-Laird

Reporting checklist for cohort study.

Based on the STROBE cohort guidelines.

Instructions to authors

Complete this checklist by entering the page numbers from your manuscript where readers will find each of the items listed below.

Your article may not currently address all the items on the checklist. Please modify your text to include the missing information. If you are certain that an item does not apply, please write "n/a" and provide a short explanation.

Upload your completed checklist as an extra file when you submit to a journal.

In your methods section, say that you used the STROBE cohortreporting guidelines, and cite them as:

von Elm E, Altman DG, Egger M, Pocock SJ, Gotzsche PC, Vandenbroucke JP. The Strengthening the Reporting of Observational Studies in Epidemiology (STROBE) Statement: guidelines for reporting observational studies.

		Reporting Item	Page Number
**Title and abstract**			
Title	#1a	Indicate the study’s design with a commonly used term in the title or the abstract	1
Abstract	#1b	Provide in the abstract an informative and balanced summary of what was done and what was found	1
**Introduction**			
Background / rationale	#2	Explain the scientific background and rationale for the investigation being reported	3
Objectives	#3	State specific objectives, including any prespecified hypotheses	3
**Methods**			
Study design	#4	Present key elements of study design early in the paper	4
Setting	#5	Describe the setting, locations, and relevant dates, including periods	4

For peer review only

		of recruitment, exposure, follow-up, and data collection	
Eligibility criteria	#6a	Give the eligibility criteria, and the sources and methods of selection of participants. Describe methods of follow-up.	4
Eligibility criteria	#6b	For matched studies, give matching criteria and number of exposed and unexposed	4
Variables	#7	Clearly define all outcomes, exposures, predictors, potential confounders, and effect modifiers. Give diagnostic criteria, if applicable	4
Data sources / measurement	#8	For each variable of interest give sources of data and details of methods of assessment (measurement). Describe comparability of assessment methods if there is more than one group. Give information separately for for exposed and unexposed groups if applicable.	4-5
Bias	#9	Describe any efforts to address potential sources of bias	10
Study size	#10	Explain how the study size was arrived at	6
Quantitative variables	#11	Explain how quantitative variables were handled in the analyses. If applicable, describe which groupings were chosen, and why	5
Statistical methods	#12a	Describe all statistical methods, including those used to control for confounding	
5			
Statistical methods	#12b	Describe any methods used to examine subgroups and interactions	5
Statistical methods	#12c	Explain how missing data were addressed	6
Statistical methods	#12d	If applicable, explain how loss to follow-up was addressed	N/A
Statistical methods	#12e	Describe any sensitivity analyses	
8			
**Results**			
Participants	#13a	Report numbers of individuals at each stage of study—eg numbers potentially eligible, examined for eligibility, confirmed eligible,	6

For peer review only

		included in the study, completing follow-up, and analysed. Give information separately for for exposed and unexposed groups if applicable.	
Participants	#13b	Give reasons for non-participation at each stage	6
Participants	#13c	Consider use of a flow diagram	
N/A			
Descriptive data	#14a	Give characteristics of study participants (eg demographic, clinical, social) and information on exposures and potential confounders. Give information separately for exposed and unexposed groups if applicable.	6
Descriptive data	#14b	Indicate number of participants with missing data for each variable of interest	
6, Supplementary Table 1			
Descriptive data	#14c	Summarise follow-up time (eg, average and total amount)	
17			
Outcome data	#15	Report numbers of outcome events or summary measures over time. Give information separately for exposed and unexposed groups if applicable.	
17			
Main results	#16a	Give unadjusted estimates and, if applicable, confounder-adjusted estimates and their precision (eg, 95% confidence interval). Make clear which confounders were adjusted for and why they were included	6-7
Main results	#16b	Report category boundaries when continuous variables were categorized	N/A
Main results	#16c	If relevant, consider translating estimates of relative risk into absolute risk for a meaningful time period	
N/A			
Other analyses	#17	Report other analyses done—eg analyses of subgroups and interactions, and sensitivity analyses	8

For peer review only

Discussion

Key results	#18	Summarise key results with reference to study objectives	9-10
Limitations	#19	Discuss limitations of the study, taking into account sources of potential bias or imprecision. Discuss both direction and magnitude of any potential bias.	10
Interpretation	#20	Give a cautious overall interpretation considering objectives, limitations, multiplicity of analyses, results from similar studies, and other relevant evidence.	10-11
Generalisability	#21	Discuss the generalisability (external validity) of the study results	10-11
**Other Information**			
Funding	#22	Give the source of funding and the role of the funders for the present study and, if applicable, for the original study on which the present article is based	1

Notes:

- 14b: 6, Supplementary Table 1 The STROBE checklist is distributed under the terms of the Creative Commons Attribution License CC-BY. This checklist was completed on 03. October 2022 using https://www.goodreports.org/, a tool made by the EQUATOR Network in collaboration with Penelope.ai

For peer review only

Anti-asthmatic prescriptions in children with and without congenital anomalies: a population-based study

Journal:	*BMJ Open*
Manuscript ID	bmjopen-2022-068885.R2
Article Type:	Original research
Date Submitted by the Author:	11-Sep-2023
Complete List of Authors:	Divin, Natalie; Ulster University Faculty of Life and Health Sciences, Institute of Nursing and Health Research<br>Given, Joanne; Ulster University Faculty of Life and Health Sciences, Institute of Nursing and Health Research<br>Tan, Joachim; St George's University of London, Population Health Research Institute<br>Astolfi, Gianni; University of Ferrara, Department of Neuroscience and Rehabilitation<br>Ballardini, Elisa; University of Ferrara, Italy, Neonatal Intensive Care Unit, Paediatric Section, University of Ferrara, Italy<br>Barrachina-Bonet, Laia; Foundation for the Promotion of Health and Biomedical Research in the Valencian region, Rare Disease Research Unit<br>Cavero- Carbonell, Clara; Foundation for the Promotion of Health and Biomedical Research in the Valencian region, Rare Diseases Research Unit<br>Coi, Alessio; National Research Council Pisa Research Area, Institute of Clinical Physiology<br>Garne, Ester; University of Southern Denmark<br>Gissler, Mika; THL National Institute for Health and Welfare, Information<br>Heino, Anna; Finnish Institute for Health and Welfare<br>Jordan, Susan; Swansea University, Nursing<br>Pierini, Anna; Institute of Clinical Physiology National Research Council<br>Scanlon, Ieuan; Swansea University<br>Urhøj, Stine Kjær ; University of Copenhagen, Section of Epidemiology, Department of Public Health<br>Morris, Joan; St George's, University of London, Population Health Research Institute<br>Loane , Maria ; Ulster University Faculty of Life and Health Sciences, Institute of Nursing and Health Research
**Primary Subject Heading**:	Paediatrics
Secondary Subject Heading:	Epidemiology, Respiratory medicine
Keywords:	PAEDIATRICS, EPIDEMIOLOGY, Risk Factors, Asthma < THORACIC MEDICINE

SCHOLARONE™
Manuscripts

I, the Submitting Author has the right to grant and does grant on behalf of all authors of the Work (as defined in the below author licence), an exclusive licence and/or a non-exclusive licence for contributions from authors who are: i) UK Crown employees; ii) where BMJ has agreed a CC-BY licence shall apply, and/or iii) in accordance with the terms applicable for US Federal Government officers or employees acting as part of their official duties; on a worldwide, perpetual, irrevocable, royalty-free basis to BMJ Publishing Group Ltd ("BMJ") its licensees and where the relevant Journal is co-owned by BMJ to the co-owners of the Journal, to publish the Work in this journal and any other BMJ products and to exploit all rights, as set out in our licence.

The Submitting Author accepts and understands that any supply made under these terms is made by BMJ to the Submitting Author unless you are acting as an employee on behalf of your employer or a postgraduate student of an affiliated institution which is paying any applicable article publishing charge ("APC") for Open Access articles. Where the Submitting Author wishes to make the Work available on an Open Access basis (and intends to pay the relevant APC), the terms of reuse of such Open Access shall be governed by a Creative Commons licence – details of these licences and which Creative Commons licence will apply to this Work are set out in our licence referred to above.

Other than as permitted in any relevant BMJ Author's Self Archiving Policies, I confirm this Work has not been accepted for publication elsewhere, is not being considered for publication elsewhere and does not duplicate material already published. I confirm all authors consent to publication of this Work and authorise the granting of this licence.

For peer review only

Anti-asthmatic prescriptions in children with and without congenital anomalies: a population-based study

Authors: Natalie Divin PhD[1], Joanne Given PhD[1], Joachim Tan PhD[2], Gianni Astolfi BSc[3], Elisa Ballardini MD[3], Laia Barrachina Bonet MSc[4], Clara Cavero PhD[4], Alessio Coi PhD[5], Ester Garne MD[6], Mika Gissler PhD[7], Anna Heino M.Soc.Sc[7], Susan Jordan PhD[8], Anna Pierini BSc[5], Ieuan Scanlon MSc[8], Stine Kjaer Urhoj PhD[9], Joan K Morris PhD[2], Maria Loane PhD[1]

Affiliations: [1]Ulster University; [2]St George's University of London; [3]Department of Neuroscience and Rehabilitation, University of Ferrara; [4]Rare Diseases Research Unit, Foundation for the Promotion of Health and Biomedical Research of Valencia Region; [5]Unit of Epidemiology of Rare Diseases and Congenital Anomalies, Institute of Clinical Physiology; [6]Pediatric Department, Hospital Lillebaelt Kolding; [7]Finnish Institute for Health and Welfare (THL); [8]Swansea University; [9]University of Copenhagen.

Word count: 3,991

Corresponding author:
Maria Loane
Ulster University, Belfast Campus, Room BC-04-167
York Street,
Belfast, Co Antrim
N Ireland BT15 1ES
Telephone: +44 2895 365256
Email: ma.loane@ulster.ac.uk

For peer review only

Abstract

Objectives: To explore the risk of being prescribed/dispensed medications for respiratory symptoms and breathing difficulties in children with and without congenital anomalies.

Design: A EUROlinkCAT population-based data linkage cohort study. Data on children with and without congenital anomalies were linked to prescription databases to identify children who did/did not receive anti-asthmatic prescriptions. Data were analysed by age, European region, class of anti-asthmatic, anomaly, sex, gestational age, and birth cohort.

Setting: Children born 2000-2014 in six regions within five European countries.

Participants: 60,662 children with congenital anomalies and 1,722,912 reference children up to age ten years.

Primary outcome measure: Relative risks (RR) of >1 anti-asthmatic prescription in a year, identified using Anatomical Therapeutic Chemical classification codes beginning with R03.

Results: There were significant differences in the prescribing of anti-asthmatics in the six regions. Children with congenital anomalies had a significantly higher risk of being prescribed anti-asthmatics (RR=1.41, 95% CI 1.35-1.48) compared to reference children. The increased risk was consistent across all regions and all age groups. Children with congenital anomalies were more likely to be prescribed beta-2 agonists (RR=1.71, 95% CI 1.60-1.83) and inhaled corticosteroids (RR=1.74, 95% CI 1.61-1.87). Children with oesophageal atresia, 3333, genetic syndromes and chromosomal anomalies had over twice the risk of being prescribed anti-asthmatics compared to reference children. Children with congenital anomalies born <32 weeks gestational age were over twice as likely to be prescribed anti-asthmatics than those born at term (RR=2.20, 95% CI 2.10-2.30).

Conclusion: This study documents the additional burden of respiratory symptoms and breathing difficulties for children with congenital anomalies, particularly those born preterm, compared to children without congenital anomalies in the first ten years of life. These findings are beneficial to clinicians and health care providers as they identify children with greater morbidity associated with respiratory symptoms, as indicated by anti-asthmatic prescriptions.

Strengths and limitations of this study

- The use of population-based data from six regions across Europe yielded a large sample size of children with and without congenital anomalies.
- Use of a Common Data Model enabled standardisation of prescription/pharmacy dispensing records across a range of coding classification systems, languages, and healthcare systems in Europe.
- Risks of being prescribed/dispensed medications for respiratory conditions were estimated in children with any major congenital anomaly, in children with 32

For peer review only - http://bmjopen.bmj.com/site/about/guidelines.xhtml

specified isolated congenital anomalies, and for within region comparisons in children with no major congenital anomaly.

- Over 95% of children across registries were linked to prescription databases limiting the potential bias from missed linkages.
- The lack of information on socio-economic status meant this potential confounding factor could not be adjusted for.

Key words: Anti-asthmatics; prescriptions; congenital anomalies; population-based; data linkage

For peer review only

For peer review only - http://bmjopen.bmj.com/site/about/guidelines.xhtml

Introduction

Breathing difficulties in childhood are a leading cause of emergency department visits, hospitalisations and missed days at school [1]. The prevalence of chronic breathing conditions such as asthma varies globally in children. The US National Health Interview Survey in 2013 reported prevalence rates of 8.3% in children aged 0-17 years based on parent reports of doctor-diagnosed asthma [2]. The International Study of Asthma and Allergies in Childhood (ISAAC) 1999-2004, which was also based on parental reports, found that prevalence of asthma in European children aged 6-7 years varied from <5% in Albania to over 20% in the United Kingdom (UK), with prevalence rates increasing yearly [3-4]. Similarly, the more recent MeDALL (Mechanisms of the Development of ALLergy) study involving eight birth cohorts across Europe found that parental reports of doctor-diagnosed asthma at age four years ranged from 1.7% in Germany to 13.5% in Bradford, England [5].

The prevalence of asthma also varies according to age and sex in childhood. The ISAAC study consistently found higher asthma rates for 13-14 year olds compared with 6-7 year olds, and this pattern of higher asthma prevalence rates in older children has been replicated across Europe [5-7]. However, as asthma can be challenging to diagnose in infants, wheezing and recurrent wheezing have been used as measures of breathing difficulties in infants. A meta-analysis of European, Latin American and African research reported prevalence rates of 36.1% (95% CI 35.2-37.0%) and 17.4% (95% CI 16.7-18.1%) for wheezing and recurring wheezing respectively for children under two years of age [8]. These prevalence rates were higher than rates of current wheeze in the ISAAC study for 6-7 year olds (11.5%) and 13-14 year olds (14.1%) [9]. Boys have an increased likelihood of parent-reported asthma and wheeze [10-12] and receive more anti-asthmatic prescriptions than girls across Denmark, Sweden and Germany [13-15].

Most of the literature on childhood asthma and respiratory conditions does not differentiate between children with and without congenital anomalies, nor does it explore the additional burden of having chronic and frequent breathing difficulties in children with congenital anomalies. A cohort study based on health records of 170,960 Canadian children up to age six, 1980-1990, reported that children with congenital anomalies of the circulatory system and anomalies of the respiratory system had an increased risk of a recorded asthma diagnosis (HR=1.16, 95% CI 1.03-1.30; and HR=1.43, 95% CI 1.27-1.61) compared to control children [16]. Children under two with diaphragmatic hernia had high hospital readmission rates for wheeze, particularly if born preterm [17]. Most research exploring asthma and breathing difficulties in those with congenital anomalies is based on case series involving small sample sizes. For example, a cohort of adult Dutch patients with unrepaired atrial septal defect reported that four out of 31 (13%) patients had an asthma diagnosis and were taking anti-asthmatic medication [18]. Similarly, a study of 14 Dutch children aged 7-12 years with oesophageal atresia identified that four of the 14 children (29%) had received a diagnosis of asthma [19].

The aim of this study is to compare the risk of being prescribed or dispensed medications for respiratory conditions in children with and without congenital anomalies in six European regions.

For peer review only - http://bmjopen.bmj.com/site/about/guidelines.xhtml

Materials and Methods

This is a EUROlinkCAT population-based data linkage cohort study involving children from 0-9 years of age with and without congenital anomalies in six European regions: Denmark: Funen; Finland; Italy: Emilia Romagna; Italy: Tuscany; Spain: Valencian Region; UK: Wales.

The inclusion criteria was all liveborn children in the registry areas born between 2000 and 2014, or the first birth year included in the study by each registry (Table 1). Five registries included reference children from the whole population covering the registry area, while Tuscany provided a 10% sample of reference children matched on year of birth and sex. Data on children with congenital anomalies were extracted from the European congenital anomaly (EUROCAT) registries and data on reference children were obtained from the birth registers in each region [20-22].

Children were linked to regional or national prescription databases and to hospital outpatient pharmacy databases, if available. Children who could not be linked to these databases were classified as "not linked" and were not included in the study, see Supplementary Table 1. Wales had information on prescriptions issued by the general practitioner (GP) for approximately 70% of Welsh GP practices that were part of the Secure Anonymised Information Linkage (SAIL) databank during the study period, while the other registries had information on prescriptions dispensed by a pharmacy in their region. None of the registries were able to link to hospital inpatient prescribing data or had access to over-the-counter medication.

Anti-asthmatic medication is defined using the World Health Organisation's Anatomical Therapeutic Chemical (ATC) classification system, i.e. ATC codes beginning with R03 (drugs for obstructive airway diseases): beta-2 agonists (R03AC); inhaled corticosteroids (R03BA); anticholinergics (R03BB); antiallergic agents (R03BC); and leukotriene receptor antagonists (R03DC). Data on beta-2 agonists and inhaled corticosteroids were explored individually. Due to small numbers, data on the remaining anti-asthmatic types were included in the 'any anti-asthmatic (R03)' category. Children were classed as exposed to anti-asthmatics if they had been prescribed or dispensed >1 anti-asthmatic prescription in a year in order to minimise children presenting with one-time occurrences of wheeze or infection. Children were classified as unexposed if they were prescribed or dispensed less than two anti-asthmatic prescriptions in a year. Data on anti-asthmatic medications were included from the year 2000 or the first birth year included in the study by each registry up to the end of 2015, resulting in at least one year of follow-up information for each child.

Information on prescriptions were standardised according to a Common Data Model developed as part of the EUROlinkCAT study protocol [21-22]. The EUROCAT data on children with congenital anomalies were already standardised according to EUROCAT guidelines [23]. A common analysis script was sent to all registries to produce aggregate data and analytic results. The aggregate data and analytic results were uploaded to a secure web portal for download by the research team; all individual case data were kept locally.

For peer review only - http://bmjopen.bmj.com/site/about/guidelines.xhtml

Registry	Total number of children included in study		Total receiving >1 anti-asthmatic during whole follow-up		Total receiving >1 inhaled corticosteroid during whole follow-up		Total receiving >1 beta-2 agonist during whole follow-up	
	**Children with congenital anomalies**	**Reference children**	**Children with congenital anomalies (%)**	**Reference children (%)**	**Children with congenital anomalies (%)**	**Reference children (%)**	**Children with congenital anomalies (%)**	**Reference children (%)**
**Denmark: Funen (2000-2014)**	1,789	72,290	614 (34.3%)	20,728 (28.7%)	288 (16.1%)	8,114 (11.2%)	284 (15.9%)	7,684 (10.6%)
**Finland (2000-2014)**	32,926	755,923	5,617 (17.1%)	99,780 (13.2%)	3,036 (9.2%)	48,866 (6.5%)	3,375 (10.3%)	53,395 (7.1%)
**Italy: Emilia Romagna (2008-2014)**	5,499	250,829	2,741 (49.8%)	117,675 (46.9%)	1,769 (32.2%)	71,088 (28.3%)	1,087 (19.8%)	41,215 (16.4%)
**Italy: Tuscany (2008-2014)**	3,048	16,844	1,392 (45.7%)	6,542 (38.8%)	962 (31.6%)	3,947 (23.4%)	560 (18.4%)	1,861 (11.0%)
**Spain: Valencian Region (2010-2014)**	4,281	223,760	1,816 (42.4%)	77,028 (34.4%)	352 (8.2%)	8,692 (3.9%)	740 (17.3%)	25,518 (11.4%)
**UK: Wales (2000-2014)**	13,119	403,266	2,540 (19.4%)	60,230 (14.9%)	1,246 (9.5%)	26,732 (6.6%)	2,018 (15.4%)	46,934 (11.6%)
**Total**	60,662	1,722,912	14,720 (24.3%)	381,983 (22.2%)	7,653 (12.6%)	167,439 (9.7%)	8,064 (13.3%)	176,607 (10.3%)

Table 1. Number and percentages of children with congenital anomalies and reference children prescribed >1 anti-asthmatic medication, by registry

For peer review only - http://bmjopen.bmj.com/site/about/guidelines.xhtml

We explored anti-asthmatic medications in children with any major congenital anomaly (defined as a congenital malformation, deformation, disruption or dysplasia) [20-21], and in 32 isolated congenital anomaly subgroups with a livebirth prevalence of ≥1.75 per 10,000 births to avoid potential issues with small numbers [21] (Supplementary Table 2). Isolated anomalies are defined as anomalies within a single organ, as defined using the EUROCAT algorithm [23].

Each participating registry obtained local approvals for their data to be used in the EUROlinkCAT project. Ethical approval for this study was obtained from Ulster University's Nursing and Health Research Ethics Filter Committee (Reference Number: FCNUR-21-060).

Patient and public involvement

No patient involved.

Statistical analysis

Person-year estimates were calculated, which considers the number of children in the study and the length of follow-up time in the study i.e. each age group includes the number of children alive at the start of that age group. The average years of follow-up for each registry are shown in Supplementary Table 1. Age groups were collapsed into six categories to avoid disclosive counts: <1 year; 1 year; 2-3 years; 4-5 years; 6-7 years; 8-9 years. Data were available from Valencian Region for children 0-5 years of age (i.e. up to the children's 6th birthday), from Italian registries for children 0-7 years of age (i.e. up to 8th birthday), and from Funen, Finland and Wales for children 0-9 years of age (i.e. up to 10th birthday). Analyses of specific congenital anomalies were conducted using data for children aged 0-7 years to include data from the Italian registries.

DerSimonian-Laird (DL) random-effects meta-analyses were conducted to identify both the relative risk (RR) of receiving anti-asthmatic prescriptions relative to the reference group, and the heterogeneity of prevalence rates across registries and by age group [24]. Random-effects meta-analyses were used to estimate RR ratios for the 32 congenital anomaly subgroups, all registries combined, and by class of anti-asthmatic i.e. any anti-asthmatic, inhaled corticosteroids and beta-2 agonists. Heterogeneity was estimated by the statistic I^2. Stata Version 17 was used for data analysis [25].

We explored the effect of receiving >1 anti-asthmatic prescription by birth cohort, sex of child, and gestational age (GA) separately for children with congenital anomalies and reference children e.g. children with congenital anomalies born in 2005-2009 were compared to children with congenital anomalies born 2000-2004; and similarly, reference children born in 2005-2009 were compared to reference children born 2000-2004. Birth cohort was examined for 2000-2004 (baseline group) and 2005-2009, using data from Funen, Finland and Wales as these were the only regions that had data on children born 2000-2004. For the remaining analyses, risk factors were examined for the 0-5 age group for all regions. Girls were used as the baseline group for sex of child analyses. Birth at 37+ weeks GA was used as the baseline group in comparison to birth at <32 weeks GA and birth at 32-36 weeks GA.

For peer review only - http://bmjopen.bmj.com/site/about/guidelines.xhtml

171 Classifying children as exposed to anti-asthmatics if they had been prescribed or dispensed
172 >1 anti-asthmatic prescription in a year was relaxed in a sensitivity analysis to include
173 children with only 1 prescription/dispensation in a year.

For peer review only

For peer review only - http://bmjopen.bmj.com/site/about/guidelines.xhtml

Results

A total of 63,911 children with congenital anomalies and 1,811,431 reference children up to and including nine years of age were included from six national/regional databases in five countries. Of these, 60,662 (94.9%) children with congenital anomalies and 1,722,912 reference children (95.1%) were included in the study. The percentage of children with and without congenital anomalies prescribed anti-asthmatic medication in each region are shown in Table 1. Overall, 5% of children (ranging from 0% in Tuscany and Valencian Region to 14.6% in Wales) were not included in the study as they could not be linked.

Prescriptions for any anti-asthmatic medicines

There were considerable geographical variations in age-specific prevalence rates of any anti-asthmatic prescription (Figure 1). However, the pattern of prescriptions according to age was similar for children with and without congenital anomalies in all registries. In four registries, the prevalence of >1 prescription for any anti-asthmatic in children with congenital anomalies and reference children peaked at the younger ages (<1 and 1 year), then decreased sharply with age. In Finland and Wales, the prevalence increased until age 2-3 years for all children and then stabilised. Geographical differences in prevalence of >1 prescription for any anti-asthmatic levelled off by age 6-7 years, with rates in children with congenital anomalies ranging between 6-9% in all registries, and rates for reference children ranging between 4-6% in all registries. Prevalence rates for >1 prescription for any anti-asthmatics were consistently slightly higher in children with congenital anomalies than reference children across registries and age groups (Figure 1).

Children with congenital anomalies had a 41% higher risk of >1 prescription for any anti-asthmatic compared to reference children across all age groups and across all registries (RR=1.41, 95% CI 1.35-1.48) (Figure 2). Children aged <1 year with congenital anomalies in Finland had an almost two-fold increase in risk compared to reference children (RR=1.97; 95% CI 1.86-2.08). The wider CIs in older age groups in some registries indicate smaller sample sizes as there were fewer children with long follow-ups. Statistically significant heterogeneity was identified between registries at all ages ($I^2 = 94.3\%$, $p < .001$) due to the large number of reference children, but the differences were small in magnitude.

Prescriptions for inhaled corticosteroids

Children with congenital anomalies had a higher risk of receiving >1 prescription for inhaled corticosteroids than reference children. However, there was heterogeneity between age groups ($p = 0.004$). The risk was highest for children with congenital anomalies aged <1 year (RR=2.12, 95% CI 1.50-2.99) and steadily decreased to 1.35 (95% CI 1.22-1.49) in children aged 8-9 years (Figure 3). The relative risks of receiving >1 prescription for inhaled corticosteroids appeared consistently higher in Valencian Region in children <1 year of age (RR=3.24, 95% CI 2.74-3.84), children at 1 year of age (RR=2.67, 95% CI 2.30-3.10), and children at 2-5 years (RR=2.16, 95% CI 1.91-2.45) compared to children in other registries. Again, there was significant statistical heterogeneity across registries for all ages ($I^2 = 95.6\%$, $p < .001$) due to the large sample sizes.

For peer review only - http://bmjopen.bmj.com/site/about/guidelines.xhtml

Prescriptions for beta-2 agonists

The RR of receiving >1 prescription for beta-2 agonists was 71% higher in children with congenital anomalies compared to reference children (RR=1.71, 95% CI 1.60-1.83) (Figure 3). In contrast to inhaled corticosteroids, the risk of receiving >1 prescription for beta-2-agonists remained consistently high across all age groups (heterogeneity between age groups $p = 0.358$). Significant heterogeneity was identified across registries for all ages ($I^2 = 94.4\%$, $p < .001$).

Risk factors for receiving anti-asthmatic prescriptions (children up to age six years)

Children aged 0-5 years were more likely to receive >1 anti-asthmatic per year in 2005-2009 compared to 2000-2004, with this risk being higher in reference children than children with congenital anomalies (Table 2). Children with congenital anomalies born at <32 weeks GA had over twice the risk of being prescribed/dispensed anti-asthmatics (RR= 2.20, 95% CI 2.10-2.30) compared to children born at 37+ weeks. The risk was also raised for reference children (RR=1.86, 95% CI 1.82-1.90). Relative risks decreased in children born 32-36 weeks GA but remained higher in children with congenital anomalies than in reference children (RR= 1.45, 95% CI 1.40-1.49; and RR= 1.31 95% CI 1.30-1.32 respectively). Boys were more likely to be prescribed anti-asthmatics than girls, with similar risks observed for children with congenital anomalies and reference children.

Congenital anomaly subgroups (children up to eight years of age)

The RR for having >1 anti-asthmatic prescription for children with specific congenital anomalies aged 0-7 years was compared to reference children. Children with oesophageal atresia had the highest risk of being prescribed/dispensed any anti-asthmatic medication (RR=3.57, 95% CI 3.14-4.06), inhaled corticosteroids (RR=5.46, 95% CI 4.46-6.68), and beta-2 agonists (RR=5.75, 95% CI 4.86-6.81), Supplementary Table 3. Children with genetic syndromes (Di George syndrome and Noonan syndrome), chromosomal anomalies (all chromosomal anomalies and Down syndrome with and without congenital heart defects), and diaphragmatic hernia had more than double the risk of having >1 prescription for any anti-asthmatics compared to reference children. Across all 32 congenital anomaly subgroups examined, children with these anomalies were significantly more likely to be prescribed any anti-asthmatic than reference children, except for children with Tetralogy of Fallot or children with hip dislocation and/or dysplasia whose risks were slightly raised but were not statistically significant.

The relative risk for receiving >1 prescription for beta-2-agonists and for inhaled corticosteroids for the 32 congenital anomaly subgroups followed the same trends as those for all anti-asthmatic medication, but they were less likely to be statistically significant due to smaller numbers being analysed.

For peer review only - http://bmjopen.bmj.com/site/about/guidelines.xhtml

	Children with congenital anomalies (RR; 95% CI)	Reference children (RR; 95% CI)
**Birth Cohort**		
2000-2004	Reference	
2005-2009	1.27 (1.23-1.31)	1.60 (1.59-1.61)
**Gestation Age**		
37+ weeks	Reference	
32-36 weeks	1.45 (1.40-1.49)	1.31 (1.30-1.32)
<32 weeks	2.20 (2.10-2.30)	1.86 (1.83-1.90)
**Sex of Child**		
Female	Reference	
Male	1.45 (1.40-1.49)	1.31 (1.30-1.32)

253

254 **Table 2.** Relative risk (RR) of >1 anti-asthmatic prescriptions in children with congenital
255 anomalies and reference children up to age six years, by risk factor
256 For the birth cohort analysis, data were analysed only from three registries: Denmark: Funen, Finland,
257 and Wales.

For peer review only

For peer review only - http://bmjopen.bmj.com/site/about/guidelines.xhtml

Sensitivity analysis

The overall RR of children with congenital anomalies receiving at least one prescription for any anti-asthmatic medication was 1.25 (95% CI 1.21-1.28) compared to reference children (Supplementary Table 4). This is compared to the RR = 1.41 (95% CI 1.35-1.48) of receiving >1 prescription. Although the risk estimates were lower for children with at least one prescription for any anti-asthmatic, the pattern of risk across registries and age groups was similar to that found for children with >1 prescription (Supplementary Figure 1).

For peer review only

Discussion

This population-based cohort study of over 1.78 million children in six European regions found that across all regions and age groups, children with congenital anomalies had a consistently higher risk of receiving prescriptions for >1 anti-asthmatic medication compared to reference children. We observed geographical variation in the use of anti-asthmatic medications with registries in Denmark, Italy and Spain having higher prevalence in young children whereas Finland and Wales had lower prevalence. By age 6-7 years, the prevalence in the individual registries started to converge for both children with and without anomalies, although it remained slightly higher for children with congenital anomalies. Children with oesophageal atresia, diaphragmatic hernia, chromosomal anomalies and specific genetic syndromes had the highest risk of being prescribed anti-asthmatic prescriptions. Male children, children born in a later cohort, and children born preterm had an increased risk of anti-asthmatic prescriptions, with children with congenital anomalies born very preterm (<32 weeks) having over double the risk of anti-asthmatic prescriptions compared to children with congenital anomalies born at term.

With the exception of Finland and Wales, our findings that anti-asthmatic prescriptions tended to be higher in younger age groups is consistent with literature indicating higher rates of wheezing and current wheeze in infants below two years of age than older children [8-9]. As beta-2 agonists and inhaled corticosteroids are recommended as treatment for infant wheeze [26], prescriptions for anti-asthmatics in young children may be indicative of wheeze and acute respiratory infections which can be common in young children [27]. However, other studies reported higher prevalence of breathing difficulties for older children in the form of parent-reported asthma [3]. It is possible that parental reports may overestimate asthma prevalence, while our study was based on administrative anti-asthmatic prescription records. We identified anti-asthmatic prevalence rates that seemed to vary from other regional studies. For example, a study based on a sample of Welsh children (n=1,529), born 2000-2002, with a GP diagnosis of asthma and/or prescriptions for anti-asthmatics in their health records in the last 12 months reported much higher prevalence rates ranging from 13.4% for 3 year olds to 10.4% in 7 year olds [28]. These differences may relate to methodology as our study criteria was >1 anti-asthmatic prescription in a year rather than at least one. We also identified regional differences within countries as within Italy, children in Emilia Romagna had a higher prevalence of >1 prescription for asthma medications than children in Tuscany. A study investigating the use of anti-asthmatics in women before, during and after pregnancy also reported this regional difference between the two Italian registries [29].

Children with congenital anomalies consistently had higher rates of anti-asthmatic medication than reference children which may be due to the fact that anti-asthmatic medications are widely used to treat respiratory complications of some anomalies. For example, congenital anomalies such as diaphragmatic hernia and Down syndrome are associated with pulmonary hypoplasia [30-33], requiring anti-asthmatics to manage wheezing episodes. Children with diaphragmatic hernia commonly require mechanical ventilation for days or weeks after birth which can lead to bronchopulmonary dysplasia [31;

For peer review only - http://bmjopen.bmj.com/site/about/guidelines.xhtml

34]. Beta-2 agonists are prescribed to children with bronchopulmonary dysplasia to reduce pulmonary resistance [35]. Studies have shown that patients with diaphragmatic hernia were over six times more likely to have been diagnosed with asthma than the general population [34]. Congenital anomalies may be comorbid with respiratory conditions, such as the associations between oesophageal atresia, Down syndrome and Di George syndrome with respiratory tract disease [36-38]. In addition, children with congenital anomalies requiring surgery and follow-up in the first year of life or those requiring routine medical care throughout their life span will have multiple interactions with the medical care team, hence there is greater opportunity for a diagnosis of respiratory difficulties to be made and anti-asthmatics to be dispensed. Indeed, an earlier EUROlinkCAT study found that children with congenital anomalies had significantly more hospital stays than children without congenital anomalies, particularly in the first year of life [39].

We did not find an increased risk for prescription of beta-2 agonists for children with Tetralogy of Fallot. Beta-2-agonists should not be used in children with infundibular pulmonary stenosis as beta-blockers may be prescribed to prevent hypoxic spells in these children while awaiting surgery [40]. While over 50% of children in hospitals are administered “unlicensed” or “off label” medicines [41-42), a Spanish study reported that 16% of anti-asthmatics dispensed by community pharmacies for use by children were “off label” and that the seasonal variations suggested that these were prescribed to treat respiratory infections in young children rather than asthma [43]. We do not have information about off label use in our study but the indications and age limits for prescribing off label anti-asthmatics are likely to differ between countries.

The finding that boys were more likely to be prescribed/dispensed anti-asthmatics is consistent with previous literature [10-15]. Both children with and without congenital anomalies had more anti-asthmatic prescriptions in 2005-2009 compared to 2000-2004. While the magnitude of risk was higher in reference children than for children with congenital anomalies, the comparisons were not between children with congenital anomalies and reference children, but within the two groups of children. Indeed, children with congenital anomalies had a slightly higher proportion of >1 anti-asthmatic prescription (24%) compared to reference children (22%). While self-reported anti-asthmatic use has increased in cohort studies of young adults between 1990-2007 [44], our findings support an increasing trend of anti-asthmatic prescriptions over time in children using administrative prescription data. Children born preterm at <32 weeks GA were twice as likely to be prescribed/dispensed anti-asthmatics than children born at term, although the risk was reduced for children born 32-36 weeks. The higher risk in children born <32 weeks GA may be due to respiratory complications such as respiratory distress syndrome or bronchopulmonary dysplasia arising from mechanical ventilation in preterm children [45-46]

A major strength of this study is that we have standardised data on congenital anomalies and a Common Data Model which enabled us to map and standardise the prescription/pharmacy dispensing records, as well as standardising demographic information on children. This is important as there are diverse coding classification systems, languages, and healthcare systems in Europe. Furthermore, this is the first population-

For peer review only - http://bmjopen.bmj.com/site/about/guidelines.xhtml

based multi-centre study exploring anti-asthmatic prescriptions in children with and without congenital anomalies which included data from 60,662 children with congenital anomalies across Europe, overcoming limitations of previous research based on small sizes. The use of reference children for comparison in each geographical region is also a major strength, as some differences in prescription rates across Europe may be explained by methodological issues (such as frequency of prescriptions issued at the pharmacy) and different level of indication for prescribing asthma medications to small children.

However, there were a number of limitations to this study. Firstly, bias may arise as approximately 5% of children across registries were unable to be linked to the prescription database due to invalid identifications numbers as was the case in Tuscany, or because their records were not sent to the national/vital statistics to be linked. In Wales, the children were registered in a GP practice that was not part of the SAIL databank, therefore they could not be linked. There is evidence suggesting that low socioeconomic status (SES) is associated with asthma [47]. Wales compared the SES of children registered in SAIL versus children not registered in SAIL and concluded that there were no differences in SES between linked/unlinked children. Similarly, Tuscany found no differences in gestational age, maternal age, sex and survival between children with congenital anomalies who were linked versus those who were not linked. Secondly, only three participating registries (Funen, Finland and Wales) could provide data for children up to and including nine years of age. Our list of anti-asthmatic medications did not include oral corticosteroids used to treat acute symptoms, but for young children this treatment may mainly be done during hospital stays, and we were unable to differentiate between long-acting and short-acting beta-2-agonists. While we explored medications prescribed and dispensed, we were unable to gauge whether these medications were actually taken.

In summary, this study demonstrates that valid information on the prevalence of anti-asthmatic prescriptions for children can be obtained through data linkage studies in order to monitor regional differences across age groups and over time. In general, children with congenital anomalies had a higher prevalence and risk of anti-asthmatic prescriptions than reference children. To evaluate the additional burden of disease that children with congenital anomalies face, it is essential to compare these children to those without congenital anomalies in the same geographic areas as prescription patterns of anti-asthmatic medications vary across Europe. The findings from this study are also beneficial to clinicians for identifying which congenital anomalies are associated with a higher risk of prescriptions for respiratory symptoms and breathing difficulties.

For peer review only

For peer review only - http://bmjopen.bmj.com/site/about/guidelines.xhtml

Contributorship Statement: ND prepared the manuscript and revised it following feedback and comments from all authors. ND analysed the pooled data from each registry. JG and ML produced the standardisation scripts. JT and JKM produced the central analysis scripts. GA, EB, LBB, CC, AC, MG, AH, SJ, AP, IS, SKU extracted and analysed local registry data. ML, EG and JKM secured funding, conceptualised and designed the study, and provided frequent feedback on manuscript drafts. JKM and ML provided data analysis guidance and syntax for meta-analyses. ML executed data analysis syntax relating to protected data.

Competing Interests: None

Funding Statement: This work was supported by the European Union's Horizon 2020 research and innovation programme under grant agreement No. 733001. The views presented here are those of the authors only, and the European Commission is not responsible for any use that may be made of the information presented here.

Data Sharing Statement: We are legally not allowed to share the third-party administrative data used in this study as it belongs to the data providers in each of the regions i.e. the regional or national statistical organisations. All our documentation is available on the EUROlinkCAT website (http://www.EUROlinkCAT.eu/wp2-buildingresultsrepository). and we encourage any interested parties to apply to the EUROlinkCAT management team to assist them in obtaining approval from the data providers in each region/ country to use the aggregated data for an approved study http://www.EUROlinkCAT.eu/contactinformationanddatarequests

Ethics Approval Statement: Ethical approval for this study was obtained from Ulster University's Nursing and Health Research Ethics Filter Committee (Reference Number: FCNUR-21-060). This study is based on secondary data analysis of data held in routine administrative databases in Europe. The individual records of children linked to electronic prescription databases remain within the regional/national statistical organisations. Only aggregated results were sent to the research team for analysis, hence informed consent was not required. Each participating registry obtained local approvals for their data to be used in the EUROlinkCAT project as described in a paper accepted for publication by the BMJ Open: Claridge H et al "Ethics and legal requirements for data linkage in 14 European countries".

For peer review only - http://bmjopen.bmj.com/site/about/guidelines.xhtml

References

1. World Health Organisation. Asthma [Internet]. World Health Organisation; 2021 [cited 2021 Aug 18]. Available from: https://www.who.int/news-room/fact-sheets/detail/asthma
2. Akinbami LJ, Simon AE, Rossen LM. Changing trends in asthma prevalence among children. Pediatrics. 2016 Jan 1;137(1).
3. Wirl C, Puklova V. Prevalence of asthma and allergies in children. *European Environment and Health Information System Fact Sheet, World Health Organization. 2007*; Fact Sheet No 3.1 (May).
4. Pearce N, Aït-Khaled N, Beasley R, Mallol J, Keil U, Mitchell E, Robertson C. Worldwide trends in the prevalence of asthma symptoms: phase III of the International Study of Asthma and Allergies in Childhood (ISAAC). Thorax. 2007 Sep 1;62(9):758-66.
5. Uphoff EP, Bird PK, Antó JM, Basterrechea M, Von Berg A, Bergström A, Bousquet J, Chatzi L, Fantini MP, Ferrero A, Gehring U. Variations in the prevalence of childhood asthma and wheeze in MeDALL cohorts in Europe. ERJ open research. 2017 Jul 1;3(3).
6. Bloom CI, Saglani S, Feary J, Jarvis D, Quint JK. Changing prevalence of current asthma and inhaled corticosteroid treatment in the UK: population-based cohort 2006–2016. European Respiratory Journal. 2019 Apr 1;53(4).
7. Sestini P, De Sario M, Bugiani M, Bisanti L, Giannella G, Kaisermann D, Frasca G, Lombardi E, Petronio MG, Dell'Orco V, Indinnimeo L. Frequency of asthma and allergies in Italian children and adolescents: results from SIDRIA-2. Epidemiologia e prevenzione. 2005 Mar 1;29(2 Suppl):24-31
8. Alvarez-Alvarez I, Niu H, Guillen-Grima F, Aguinaga-Ontoso I. Meta-analysis of prevalence of wheezing and recurrent wheezing in infants. Allergologia et immunopathologia. 2018 May 1;46(3):210-7.
9. Lai CK, Beasley R, Crane J, Foliaki S, Shah J, Weiland S, ISAAC Phase Three Study Group. Global variation in the prevalence and severity of asthma symptoms: phase three of the International Study of Asthma and Allergies in Childhood (ISAAC). Thorax. 2009 Jun 1;64(6):476-83.
10. Duggan EM, Sturley J, Fitzgerald AP, Perry IJ, Hourihane JO. The 2002–2007 trends of prevalence of asthma, allergic rhinitis and eczema in Irish schoolchildren. Pediatric allergy and immunology. 2012 Aug;23(5):464-71.
11. Ciccone G, Camarlengo A, Bugiani M, Dalmasso P, Faggiano F, Volante T, Magnani C, Natale P, Piccioni P, Bisanti L, Gianelle V. Asthma and respiratory symptoms in 6-7 yr old Italian children: gender, latitude, urbanization and socioeconomic factors. Eur Respir J. 1997 Aug 1;10:1780-6.
12. Linneberg A, Simonsen JB, Petersen J, Stensballe LG, Benn CS. Differential effects of risk factors on infant wheeze and atopic dermatitis emphasize a different etiology. Journal of Allergy and Clinical Immunology. 2006 Jan 1;117(1):184-9.
13. Henriksen L, Simonsen J, Haerskjold A, Linder M, Kieler H, Thomsen SF, Stensballe LG. Incidence rates of atopic dermatitis, asthma, and allergic rhinoconjunctivitis in

For peer review only

For peer review only - http://bmjopen.bmj.com/site/about/guidelines.xhtml

Danish and Swedish children. Journal of Allergy and Clinical Immunology. 2015 Aug 1;136(2):360-6.

14. Hoffmann F, Glaeske G. Prescriptions as a proxy for asthma in children: a good choice?. European journal of clinical pharmacology. 2010 Mar;66(3):307-13.
15. Stock S, Redaelli M, Luengen M, Wendland G, Civello D, Lauterbach KW. Asthma: prevalence and cost of illness. European Respiratory Journal. 2005 Jan 1;25(1):47-53.
16. Dik N, Tate RB, Manfreda J, Anthonisen NR. Risk of physician-diagnosed asthma in the first 6 years of life. Chest. 2004 Oct 1;126(4):1147-53.
17. Benoist G, Mokhtari M, Deschildre A, Khen-Dunlop N, Storme L, Benachi A, Delacourt C. Risk of readmission for wheezing during infancy in children with congenital diaphragmatic hernia. PloS one. 2016 May 12;11(5):e0155556
18. Nassif M, van Steenwijk RP, Hogenhout JM, Lu H, de Bruin-Bon RH, Hirsch A, Sterk PJ, Bouma BJ, Straver B, Tijssen JG, Mulder BJ. Atrial septal defect in adults is associated with airway hyperresponsiveness. Congenital heart disease. 2018 Nov;13(6):959-66.
19. Agrawal L, Beardsmore CS, MacFadyen UM. Respiratory function in childhood following repair of oesophageal atresia and tracheoesophageal fistula. Archives of disease in childhood. 1999 Nov 1;81(5):404-8.
20. Boyd PA, Haeusler M, Barisic I, Loane M, Garne E, Dolk H. Paper 1: The EUROCAT network—organization and processes. *Birth Defects Research Part A, Clinical and Molecular Teratology.* 2011;91(S1):S2-S15.
21. Morris JK, Garne E, Loane M, Barisic I, Densem J, Latos-Bieleńska A, et al. EUROlinkCAT protocol for a European population-based data linkage study investigating the survival, morbidity and education of children with congenital anomalies. BMJ Open. 2021;11(6):e047859.
22. Loane M, Given JE, Tan J, Astolfi G, Barisic I, Carbonell CC, et al. (2021) Creating a population-based cohort of children born with and without congenital anomalies using birth data linked to hospital discharge databases in 11 European regions: assessment of linkage success and data quality. PLoS ONE (submitted)
23. EUROCAT (2020). EUROCAT Guide 1.4: instruction for the registration of congenital anomalies. EUROCAT Central Registry, Ulster University
24. DerSimonian R, Laird N. Meta-analysis in clinical trials. Control Clin Trials. 1986; 7(3):177–88. https://doi.org/10.1016/0197-2456(86)90046-2.
25. StataCorp. 2021. *Stata Statistical Software: Release 17*. College Station, TX: StataCorp LLC.
26. Brand PL, Baraldi E, Bisgaard H, Boner AL, Castro-Rodriguez JA, Custovic A, de Blic J, De Jongste JC, Eber E, Everard ML, Frey U. Definition, assessment and treatment of wheezing disorders in preschool children: an evidence-based approach. European Respiratory Journal. 2008 Oct 1;32(4):1096-110.
27. Nair H, Nokes DJ, Gessner BD, Dherani M, Madhi SA, Singleton RJ, O'Brien KL, Roca A, Wright PF, Bruce N, Chandran A. Global burden of acute lower respiratory infections due to respiratory syncytial virus in young children: a systematic review and meta-analysis. The Lancet. 2010 May 1;375(9725):1545-55.

For peer review only - http://bmjopen.bmj.com/site/about/guidelines.xhtml

28. Griffiths LJ, Lyons RA, Bandyopadhyay A, et al. Childhood asthma prevalence: cross-sectional record linkage study comparing parent-reported wheeze with general practitioner-recorded asthma diagnoses from primary care electronic health records in Wales. BMJ Open Resp Res 2018;5:e000260. doi:10.1136/ bmjresp-2017-000260
29. Charlton RA, Pierini A, Klungsøyr K, Neville AJ, Jordan S, Thayer D, Bos HJ, Puccini A, Hansen AV, Gini R, Engeland A. Asthma medication prescribing before, during and after pregnancy: a study in seven European regions. BMJ open. 2016 Jan 1;6(1):e009237.
30. Cooney TP, Thurlbeck WM. Pulmonary hypoplasia in Down's syndrome. New England Journal of Medicine. 1982 Nov 4;307(19):1170-3.
31. Ijsselstijn H, Tibboel D, Hop WJ, Molenaar JC, de Jongste JC. Long-term pulmonary sequelae in children with congenital diaphragmatic hernia. Am J Respir Crit Care Med 1997;155:174–180.
32. Logan JW, Rice HE, Goldberg RN, Cotten CM. Congenital diaphragmatic hernia: a systematic review and summary of best-evidence practice strategies. Journal of Perinatology. 2007 Sep;27(9):535-49.
33. Wang J, Liu H, Gao L, Liu X. Impaired FGF10 signaling and epithelial development in experimental lung hypoplasia with esophageal atresia. Frontiers in pediatrics. 2018 Apr 20;6:109.
34. Spoel M, van der Cammen-van Zijp MH, Hop WC, Tibboel D, de Jongste JC, IJsselstijn H. Lung function in young adults with congenital diaphragmatic hernia; a longitudinal evaluation. Pediatric pulmonology. 2013 Feb;48(2):130-7.
35. Pfenninger J, Aebi C. Respiratory response to salbutamol (albuterol) in ventilator-dependent infants with chronic lung disease: pressurized aerosol delivery versus intravenous injection. Intensive care medicine. 1993 May;19(5):251-5.
36. Usui N, Kamata S, Ishikawa S, Sawai T, Okuyama H, Imura K, Okada A. Anomalies of the tracheobronchial tree in patients with esophageal atresia. Journal of pediatric surgery. 1996 Feb 1;31(2):258-62.
37. Verheij E, Speleman L, van der Molen AB, Thomeer HG. Congenital respiratory tract disorders in 22q11. 2 deletion syndrome. International journal of pediatric otorhinolaryngology. 2018 Jan 1;104:1-4.
38. Colvin KL, Yeager ME. What people with Down Syndrome can teach us about cardiopulmonary disease. European Respiratory Review. 2017 Mar 31;26(143).
39. Urhoj SK, Tan J, Morris JK, Given J, Astolfi G, et al... (2022) Hospital length of stay among children with and without congenital anomalies across 11 European regions—A population-based data linkage study. PLoS ONE 17(7): https://doi.org/10.1371/journal.pone.0269874
40. Conroy S, Choonara I, Impicciatore P, Mohn A, Arnell H, et al. Survey of unlicensed and off label drug use in paediatric wards in European countries. European Network for Drug Investigation in Children. BMJ. 2000 Jan 8;320(7227):79-82. doi: 10.1136/bmj.320.7227.79.
41. Gade C, Trolle S, Mørk ML, Lewis A, Andersen PF, et al. Massive presence of off-label medicines in Danish neonatal departments: A nationwide survey using national hospital purchase data. Pharmacol Res Perspect. 2023 Feb;11(1):e01037. doi: 10.1002/prp2.1037.Casares Alonso I, Cano Garcinuño A, Blanco Quirós A, Pérez

For peer review only - http://bmjopen.bmj.com/site/about/guidelines.xhtml

García I. Off-label prescription of anti-asthmatic agents in Primary Care. Rev Pediatr Aten Primaria. 2015;17:237-46

42. Casares Alonso I, Cano Garcinuño A, Blanco Quirós A, Pérez García I. Off-label prescription of anti-asthmatic agents in Primary Care. Rev Pediatr Aten Primaria. 2015;17:237-46.
43. Fanous E, Mogyorósy G. Does the prophylactic and therapeutic use of beta-blockers in preoperative patients with tetralogy of Fallot significantly prevent and treat the occurrence of cyanotic spells? Interactive cardiovascular and thoracic surgery. 2017 Oct 1;25(4):647-50.
44. Sigurkarlsson S, Clausen M, Gislason T, Gislason D. Prevalence of respiratory symptoms and use of asthma drugs are increasing among young adult Icelanders. Laeknabladid. 2011 Sep 1;97(9):463-7.
45. Fraser J, Walls M, McGuire W. Respiratory complications of preterm birth. Bmj. 2004 Oct 21;329(7472):962-5.
46. Xu YP. Bronchopulmonary dysplasia in preterm infants born at less than 32 weeks gestation. Global pediatric health. 2016 Sep 13;3:2333794X16668773.
47. Uphoff E, Cabieses B, Pinart M, et al. A systematic review of socioeconomic position in relation to asthma and allergic diseases. Eur Respir J 2015; 46: 364–374

For peer review only

For peer review only - http://bmjopen.bmj.com/site/about/guidelines.xhtml

Legends

Figure 1. Prevalence of >1 prescription for any anti-asthmatic per 100 children in each age group, by registry

Figure 2. Relative risk (RR) of >1 prescription for any anti-asthmatic (AA) in children with congenital anomalies (CAs) compared to reference children, by registry
CI=Confidence intervals,
DL=DerSimonian-Laird

Figure 3. Relative risk (RR) of >1 beta-2 agonist (B2A) prescription and >1 inhaled corticosteroid (ICS) prescription in children with congenital anomalies (CAs) compared to reference children, by registry
CI=Confidence interval
DL=DerSimonian-Laird

For peer review only

For peer review only - http://bmjopen.bmj.com/site/about/guidelines.xhtml

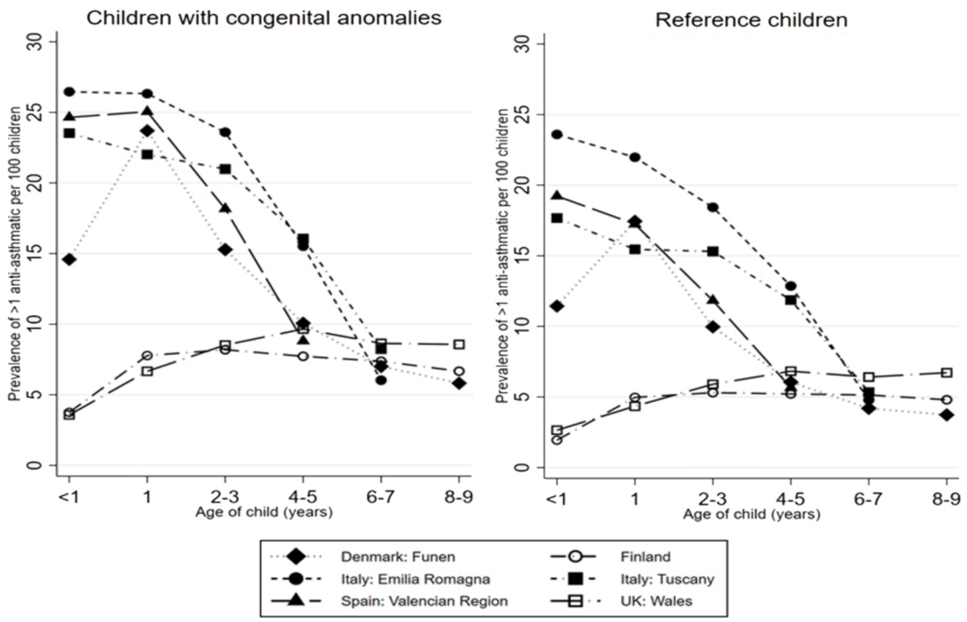

Figure 1. Prevalence of >1 prescription for any anti-asthmatic per 100 children in each age group, by registry

582x366mm (72 x 72 DPI)

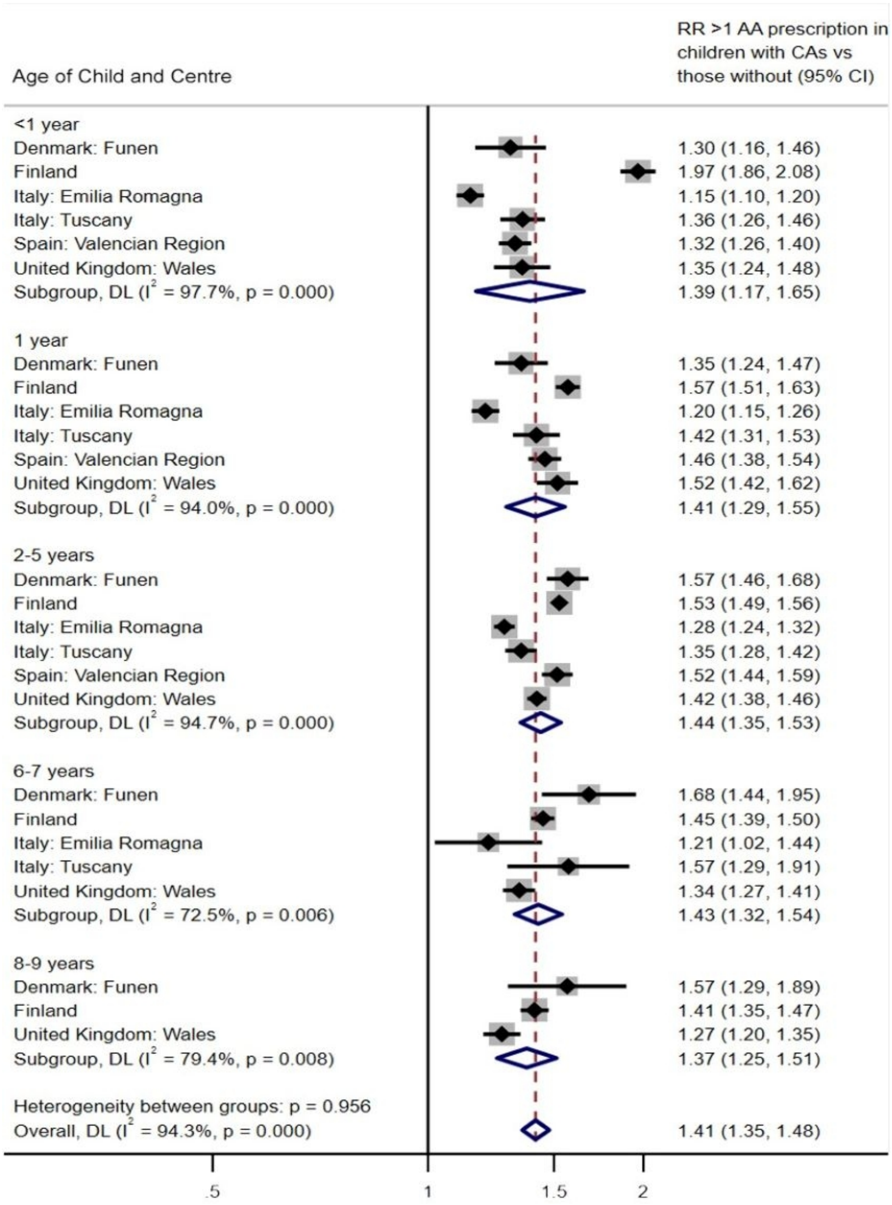

Figure 2. Relative risk (RR) of >1 prescription for any anti-asthmatic (AA) in children with congenital anomalies (CAs) compared to reference children, by registry
CI=Confidence intervals,
DL=DerSimonian-Laird

300x405mm (72 x 72 DPI)

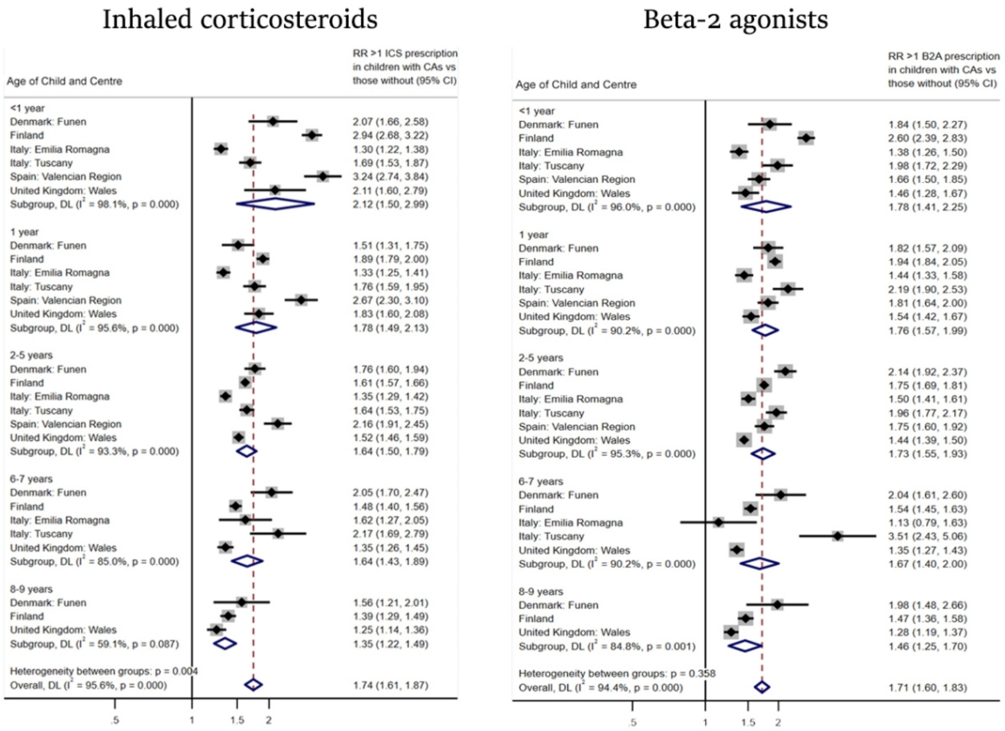

Figure 3. Relative risk (RR) of >1 beta-2 agonist (B2A) prescription and >1 inhaled corticosteroid (ICS) prescription in children with congenital anomalies (CAs) compared to reference children, by registry
CI=Confidence interval
DL=DerSimonian-Laird

426x318mm (72 x 72 DPI)

Registry	Birth years included	Average follow-up years (years)	Total number of children in population		Number of children included in the study		Number of children not linked*	
			Children with congenital anomalies	Reference children	Children with congenital anomalies (%)	Reference children (%)	Children with congenital anomalies (%)	Reference children (%)
**Denmark: Funen**	2000-2014	7.3	1,813	72,382	1,789 (98.7)	72,290 (99.9)	24 (1.3)	92 (0.1)
**Finland**	2000-2014	7.2	33,177	762,989	32,926 (99.2)	755,923 (99.1)	251 (0.8)	7,066 (0.9)
**Italy: Emilia Romagna**	2008-2014	4.6	5,801	263,574	5,499 (94.8)	250,829 (95.2)	302 (5.2)	12,745 (4.8)
**Italy: Tuscany**	2008-2014	4.4	3,445	16,844	3,048 (88.5)	16,844 (100)	397 (11.5)	0
**Spain: Valencian Region**	2010-2014	3.6	4,308	223,760	4,281 (99.4)	223,760 (100)	27 (0.6)	0
**UK: Wales**	2000-2014	6.7	15,367	471,882	13,119 (85.4)	403,266 (85.5)	2,248 (14.6)	68,616 (14.5)
**Total**			63,911	1,811,431	60,662 (94.9)	1,722,912 (95.1)	3,249 (5.1)	88,519 (4.9)

*These children were excluded from the study

Supplementary Table 1. Number and percentage of children included in the study by registry

For peer review only

Type of Anomaly*	Congenital Anomaly	ICD10-BPA Code	ICD9 Code
Isolated*	Spina bifida	Q05	741
	Hydrocephalus	Q03	7423
	Anomalies of the corpus callosum	Q040	74221
	Severe microcephaly	Q02	7421
	Congenital heart defects (CHD)**	Q20-Q26	745, 746, 7470-7474
	Severe CHD***	Q200, Q201, Q203, Q204, Q212, Q213, Q220, Q224, Q225, Q226, Q230, Q232, Q233, Q234, Q251, Q252, Q262	74500, 74510, 7452, 7453, 7456, 7461, 7462, 74600, 7463, 7465, 7466, 7467, 7471, 74720, 74742
	Transposition of great arteries	Q203	74510
	Ventricular septal defect	Q210	7454
	Atrial septal defect	Q211	7455
	Tetralogy of Fallot	Q213	7452
	Coarctation of the aorta	Q251	7471
	Patent ductus arteriosus	Q250	7470
	Cleft lip (with or without cleft palate)	Q36, Q37	7491, 7492
	Cleft palate	Q35	7490
	Oesophageal atresia	Q390-Q391	75030-75031
	Anorectal atresia	Q420-Q423	75121-75124
	Diaphragmatic hernia	Q790	75661
	Gastroschisis	Q793	75671
	Multicystic renal dysplasia	Q6140, Q6141	75316
	Congenital hydronephrosis	Q620	75320
	Hypospadias	Q54	75260
	Limb reduction defects	Q71-Q73	7552-7554
	Club foot	Q660	75450
	Hip dislocation and/or dysplasia	Q650-Q652, Q6580, Q6581	75430
	Craniosynostosis	Q750	75600
Chromosomal	Down syndrome	Q90	7580
	Down syndrome without CHD	Q90 excluding codes Q20-Q26	7580 excluding codes 745, 746, 7470-7474

For peer review only

	Down syndrome with CHD	Q90 including codes Q20-Q26	7580 including codes 745, 746, 7470-7474
	Turner syndrome	Q96	75860, 75861, 75862, 75869
Genetic syndromes	Di George syndrome	D821	27910
	Noonan syndrome	Q8714	759896

Supplementary Table 2. Congenital anomalies and relevant International Classification of Diseases (ICD)-British Paediatric Association (BPA) codes

*Based on the EUROCAT algorithm [13]

**CHD includes all the anomalies listed under severe CHD, see below, as well as atrial septal defect, ventricular septal defect, pulmonary valve stenosis and patent ductus arteriosus in term infants

***Severe CHD included the following CHD subgroups: common arterial truncus, double outlet right ventricle, transposition of great vessels, single ventricle, atrioventricular septal defect, tetralogy of Fallot, pulmonary valve atresia, tricuspid atresia and stenosis, Ebstein anomaly, hypoplastic right heart, aortic valve atresia/stenosis, mitral valve anomalies, hypoplastic left heart, coarctation of aorta, aortic atresia/interrupted aortic arch, total anomalous pulmonary venous return.

For peer review only

Isolated Anomaly	Any Anti-Asthmatic (RR, 95% CI)	Beta-2 Agonists (RR, 95% CI)	Inhaled Corticosteroids (RR, 95% CI)
**Spina bifida** n = 154	1.68 (1.35-2.09)	2.53 (1.89-3.38)	2.52 (1.97-3.23)
**Hydrocephalus** n = 299	1.59 (1.41-1.80)	2.24 (1.86-2.69)	2.04 (1.71-2.45)
**Severe microcephaly** n = 278	1.53 (1.30-1.79)	2.15 (1.63-2.84)	1.82 (1.44-2.31)
**Anomalies of corpus callosum** n = 471	1.94 (1.70-2.22)	3.44 (2.90-4.08)	2.90 (2.53-3.33)
**Congenital heart defects (CHD)** n = 19,643	1.31 (1.27-1.36)	1.47 (1.40-1.54)	1.48 (1.39-1.57)
**Severe CHD** n = 3,368	1.42 (1.33-1.52)	1.60 (1.47-1.74)	1.83 (1.65-2.02)
**Transposition of great arteries** n = 488	1.46 (1.28-1.66)	1.91 (1.62-2.24)	2.10 (1.81-2.43)
**Ventricular septal defect** n = 11,826	1.21 (1.18-1.24)	1.35 (1.30-1.41)	1.26 (1.20-1.31)
**Atrial septal defect** n = 2,679	1.43 (1.33-1.54)	1.51 (1.37-1.67)	1.53 (1.37-1.70)
**Tetralogy of Fallot** n = 426	1.10 (0.97-1.25)	1.19 (0.94-1.49)	1.52 (1.26-1.84)
**Coarctation of aorta** n = 1,021	1.72 (1.56-1.90)	2.09 (1.85-2.36)	2.12 (1.88-2.38)
**Patent ductus arteriosus** n = 385	1.57 (1.40-1.76)	2.24 (1.81-2.77)	2.23 (1.84-2.71)
**Cleft lip (with or without cleft palate)** n = 1,299	1.27 (1.19-1.36)	1.62 (1.47-1.80)	1.31 (1.18-1.45)
**Cleft palate** n = 1,164	1.49 (1.35-1.65)	1.79 (1.49-2.15)	1.71 (1.49-1.96)
**Oesophageal atresia** n = 239	3.57 (3.14-4.06)	5.75 (4.86-6.81)	5.46 (4.46-6.68)
**Anorectal atresia** n = 236	1.40 (1.20-1.64)	1.92 (1.48-2.49)	1.82 (1.45-2.28)
**Diaphragmatic hernia** n = 211	2.28 (1.72-3.03)	3.70 (2.85-4.81)	3.34 (2.48-4.50)
**Gastroschisis** n = 376	1.45 (1.27-1.67)	1.85 (1.52-2.25)	1.44 (1.15-1.80)

**Multicystic renal dysplasia** n = 621	1.31 (1.18-1.46)	1.83 (1.58-2.11)	1.56 (1.33-1.83)
**Congenital hydronephrosis** n = 3,026	1.21 (1.15-1.28)	1.33 (1.20-1.46)	1.27 (1.18-1.38)
**Hypospadias** n = 2,526	1.30 (1.22-1.39)	1.52 (1.37-1.69)	1.44 (1.32-1.57)
**Limb reduction defects** n = 445	1.16 (1.03-1.32)	1.40 (1.14-1.72)	1.32 (1.06-1.64)
**Club foot** n = 1,997	1.31 (1.24-1.38)	1.46 (1.34-1.59)	1.42 (1.31-1.54)
**Hip dislocation and/or dysplasia** n = 1,026	1.05 (0.96-1.15)	1.03 (0.91-1.17)	1.22 (1.07-1.39)
**Craniosynostosis** n = 765	1.37 (1.26-1.49)	1.70 (1.50-1.93)	1.46 (1.27-1.67)
**All chromosomal** n = 3,608	2.19 (2.00-2.40)	2.86 (2.47-3.31)	2.94 (2.58-3.34)
**Down syndrome** n = 1,993	2.19 (2.00-2.40)	2.77 (2.40-3.20)	2.88 (2.54-3.28)
**Down syndrome (including CHD)** n = 1,131	2.33 (2.09-2.61)	2.93 (2.47-3.48)	3.12 (2.70-3.61)
**Down syndrome (excluding CHD)** n = 862	2.11 (1.93-2.31)	2.76 (2.39-3.19)	2.73 (2.31-3.23)
**Turner syndrome** n = 143	1.48 (1.21-1.82)	2.50 (1.91-3.28)	2.15 (1.64-2.82)
**Di George syndrome** n = 172	3.47 (3.04-3.95)	5.11 (4.35-6.01)	4.88 (4.01-5.94)
**Noonan syndrome** n = 120	2.33 (1.99-2.72)	2.76 (2.09-3.64)	3.68 (2.82-4.82)

Supplementary Table 3. Combined relative risks (RR) across registries for >1 anti-asthmatic prescriptions up to age eight years, by anomaly

	RR of receiving ≥1 prescription for any anti-asthmatic compared to reference children	RR of receiving >1 prescription for any anti-asthmatic compared to reference children
**<1 year**	1.17 (1.10-1.25)	1.39 (1.17-1.65)
**1 year**	1.22 (1.17-1.27)	1.41 (1.29-1.55)
**2-5 years**	1.27 (1.19-1.35)	1.44 (1.35-1.53)
**6-7 years**	1.33 (1.26-1.41)	1.43 (1.32-1.54)
**8-9 years**	1.30 (1.19-1.41)	1.37 (1.25-1.51)
**All years combined**	1.25 (1.21-1.28)	1.41 (1.35-1.48)

Supplementary Table 4. Relative risk (RR) of receiving ≥1 prescription versus receiving >1 prescription for any anti-asthmatic in children with congenital anomalies compared to reference children

For peer review only

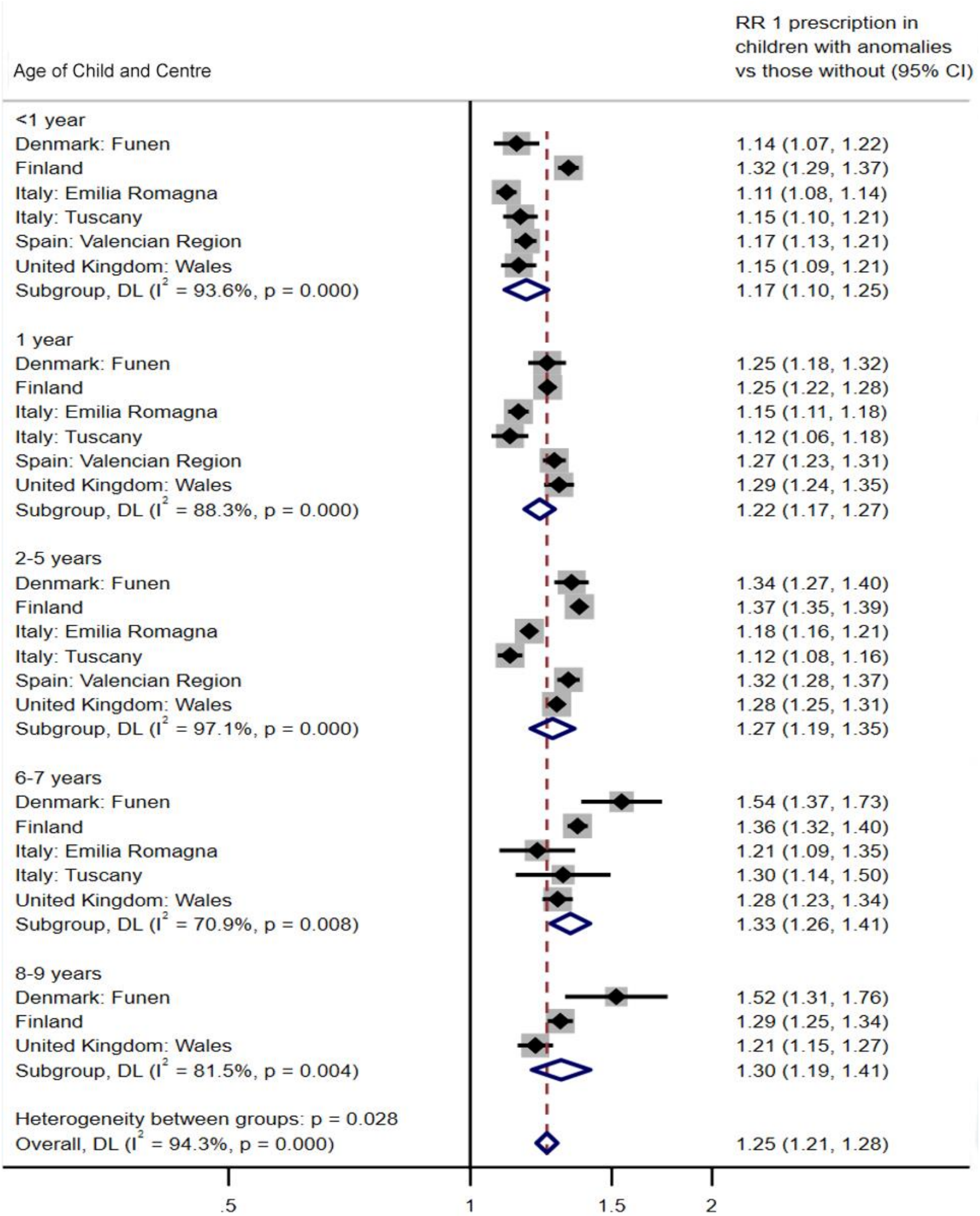

Supplementary Figure 1. Relative risk (RR) of at least 1 prescription for any anti-asthmatic in children with congenital anomalies (CAs) compared to reference children, by registry
CI=Confidence interval,
DL=DerSimonian-Laird

Reporting checklist for cohort study.

Based on the STROBE cohort guidelines.

Instructions to authors

Complete this checklist by entering the page numbers from your manuscript where readers will find each of the items listed below.

Your article may not currently address all the items on the checklist. Please modify your text to include the missing information. If you are certain that an item does not apply, please write "n/a" and provide a short explanation.

Upload your completed checklist as an extra file when you submit to a journal.

In your methods section, say that you used the STROBE cohortreporting guidelines, and cite them as:

von Elm E, Altman DG, Egger M, Pocock SJ, Gotzsche PC, Vandenbroucke JP. The Strengthening the Reporting of Observational Studies in Epidemiology (STROBE) Statement: guidelines for reporting observational studies.

For peer review only

		Reporting Item	Page Number
**Title and abstract**			
Title	#1a	Indicate the study’s design with a commonly used term in the title or the abstract	1
Abstract	#1b	Provide in the abstract an informative and balanced summary of what was done and what was found	1
**Introduction**			
Background / rationale	#2	Explain the scientific background and rationale for the investigation being reported	3
Objectives	#3	State specific objectives, including any prespecified hypotheses	3
**Methods**			
Study design	#4	Present key elements of study design early in the paper	4
Setting	#5	Describe the setting, locations, and relevant dates, including periods	4

		of recruitment, exposure, follow-up, and data collection	
Eligibility criteria	#6a	Give the eligibility criteria, and the sources and methods of selection of participants. Describe methods of follow-up.	4
Eligibility criteria	#6b	For matched studies, give matching criteria and number of exposed and unexposed	4
Variables	#7	Clearly define all outcomes, exposures, predictors, potential confounders, and effect modifiers. Give diagnostic criteria, if applicable	4
Data sources / measurement	#8	For each variable of interest give sources of data and details of methods of assessment (measurement). Describe comparability of assessment methods if there is more than one group. Give information separately for for exposed and unexposed groups if applicable.	4-5
Bias	#9	Describe any efforts to address potential sources of bias	10
Study size	#10	Explain how the study size was arrived at	6
Quantitative variables	#11	Explain how quantitative variables were handled in the analyses. If applicable, describe which groupings were chosen, and why	5
Statistical methods	#12a	Describe all statistical methods, including those used to control for confounding	
5			
Statistical methods	#12b	Describe any methods used to examine subgroups and interactions	5
Statistical methods	#12c	Explain how missing data were addressed	6
Statistical methods	#12d	If applicable, explain how loss to follow-up was addressed	N/A
Statistical methods	#12e	Describe any sensitivity analyses	
8			
**Results**			
Participants	#13a	Report numbers of individuals at each stage of study—eg numbers potentially eligible, examined for eligibility, confirmed eligible,	6

For peer review only

		included in the study, completing follow-up, and analysed. Give information separately for for exposed and unexposed groups if applicable.	
Participants	#13b	Give reasons for non-participation at each stage	6
Participants	#13c	Consider use of a flow diagram	
N/A			
Descriptive data	#14a	Give characteristics of study participants (eg demographic, clinical, social) and information on exposures and potential confounders. Give information separately for exposed and unexposed groups if applicable.	6
Descriptive data	#14b	Indicate number of participants with missing data for each variable of interest	
6, Supplementary Table 1			
Descriptive data	#14c	Summarise follow-up time (eg, average and total amount)	
17			
Outcome data	#15	Report numbers of outcome events or summary measures over time. Give information separately for exposed and unexposed groups if applicable.	
17			
Main results	#16a	Give unadjusted estimates and, if applicable, confounder-adjusted estimates and their precision (eg, 95% confidence interval). Make clear which confounders were adjusted for and why they were included	6-7
Main results	#16b	Report category boundaries when continuous variables were categorized	N/A
Main results	#16c	If relevant, consider translating estimates of relative risk into absolute risk for a meaningful time period	
N/A			
Other analyses	#17	Report other analyses done—eg analyses of subgroups and interactions, and sensitivity analyses	8

For peer review only

Discussion

Key results	#18	Summarise key results with reference to study objectives	9-10
Limitations	#19	Discuss limitations of the study, taking into account sources of potential bias or imprecision. Discuss both direction and magnitude of any potential bias.	10
Interpretation	#20	Give a cautious overall interpretation considering objectives, limitations, multiplicity of analyses, results from similar studies, and other relevant evidence.	10-11
Generalisability	#21	Discuss the generalisability (external validity) of the study results	10-11

Other Information

Funding	#22	Give the source of funding and the role of the funders for the present study and, if applicable, for the original study on which the present article is based	1

Notes:

- 14b: 6, Supplementary Table 1 The STROBE checklist is distributed under the terms of the Creative Commons Attribution License CC-BY. This checklist was completed on 03. October 2022 using https://www.goodreports.org/, a tool made by the EQUATOR Network in collaboration with Penelope.ai

For peer review only
